# A multiphysics computational model of focused ultrasound-enhanced drug delivery using temperature-sensitive liposomes

**DOI:** 10.1007/s10237-026-02075-5

**Published:** 2026-06-03

**Authors:** Marina Koutsi, Fotios Mpekris, Triantafyllos Stylianopoulos

**Affiliations:** 1 Cancer Biophysics Laboratory, Department of Mechanical and Manufacturing Engineering, University of Cyprus, Nicosia, Cyprus; 2 Cancer Genetics, Therapeutics and Ultrastructural Pathology Department, The Cyprus Institute of Neurology and Genetics, Nicosia, Cyprus

**Keywords:** Mathematical model, Ultrasound hyperthermia, Thermosensitive liposomes, Controlled drug release, Tumor microenvironment

## Abstract

The efficacy of conventional chemotherapy in solid tumors remains limited due to tumor microenvironment barriers that impede efficient drug transport and compromise therapeutic outcomes. Thermosensitive liposomes (TSLs) combined with focused ultrasound-induced hyperthermia offer a promising strategy for localized, temperature-triggered drug release. Despite experimental progress, a quantitative understanding of the coupled physical and biological mechanisms underlying this therapy is yet to be fully elucidated. Here, a three-dimensional multiphysics computational model was developed to investigate the interplay between focused ultrasound-induced hyperthermia and temperature-sensitive liposome-mediated drug delivery in solid tumors, integrating acoustic propagation, tissue heating and temperature-dependent drug release. Model predictions were validated against published experimental data, demonstrating good agreement with tumor growth and intratumoral drug concentration data. Sensitivity analysis showed that focused ultrasound parameters and liposome properties strongly influence treatment efficacy. Prolonged focused ultrasound exposure (20–30 min) produced greater tumor reduction than frequency variations (2–5 MHz). Treatment timing was also critical: For highly proliferating tumors, early therapy yielded markedly improved outcomes. Faster drug release kinetics enhanced intracellular drug accumulation and tumor regression. Intermediate-sized TSLs (**~ **50 nm in radius) achieved optimal efficacy under moderate vascular permeability conditions, while larger liposomes (~ 65 nm in radius) were more effective in tumors with highly permeable vessels due to increased extravasation. This work provides a computational framework with predictive potential for optimizing the combined focused ultrasound-thermosensitive liposomes therapy and assisting to the design of thermally triggered nanocarriers.

**Supplementary Information:**

The online version contains supplementary material available at 10.1007/s10237-026-02075-5.

## Introduction

The efficacy of conventional chemotherapy is often limited by pathophysiological barriers that hinder drug penetration into solid tumors. Solid tumors consist not only of malignant cells but also of stromal cells and a dense extracellular matrix (ECM), forming the complex tumor microenvironment (TME) (Jain 2008, 2013; Vakoc et al. 2009; Erkan et al. 2012; Stylianopoulos et al. 2018). Tumor progression might be accompanied by extensive fibrosis, characterized by excessive deposition of ECM components, mainly collagen and hyaluronan, which markedly increase tissue stiffness and alter the tumor’s mechanical properties (Mpekris et al. 2024, Angeli et al. 2025, Neophytou et al. 2025b). The uncontrolled proliferation of cancer cells within the confined host tissue, combined with the high density of stromal components, and extracellular matrix elements, generates solid stress, compressing intratumoral vessels, reducing microvascular density and often causing vascular collapse (Jain et al. 2014; Stylianopoulos 2017). The remaining vessels are typically leaky, leading to elevated interstitial fluid pressure (IFP) (Stylianopoulos et al. 2018). These vascular dysfunctions result in hypoperfusion, which limits the delivery of oxygen to the tumors, causing hypoxia (Padera et al. 2004; Stylianopoulos et al. 2012, 2013). They also impede effective delivery of drugs and promote tumor progression and chemoresistance (Jain 2014; Kalli et al. 2024, Mosoane et al. 2025).

The limited effectiveness and significant adverse effects of conventional chemotherapeutic agents have spurred the emergence of nanomedicine, which uses drug-loaded nanocarriers to deliver therapeutics directly to target sites (Bhatia et al. 2022; Kashkooli et al. 2022). Various nanosized drug delivery systems, including liposomes, micelles and macromolecular carriers, enable controlled, stimuli-responsive drug release (e.g., triggered by temperature or pH), enhancing local drug accumulation while minimizing systemic toxicity (Bhatia et al. 2022; Kashkooli et al. 2022; Moradi Kashkooli et al. 2023a, b; Tehrani et al. 2024). Among these, thermosensitive liposomes (TSLs) aim at enhancing therapeutic efficacy when combined with mild local hyperthermia (HT), releasing most of their drug payload at temperatures within 40–43 °C, undergoing a phase transition from a solid-ordered to a liquid-disordered state and thus making release kinetics highly dependent on local tissue temperature (Kong et al. 2000, Needham and Dewhirst 2001, Li et al. 2010, Willerding et al. 2016, Lokerse et al. 2018, Zhan and Wang 2018, Rezaeian et al. 2019, Namakshenas and Mojra 2023). High-intensity focused ultrasound (HIFU) is a precise, controllable and noninvasive therapeutic modality that converts absorbed acoustic energy into heat, enabling localized and well-defined temperature elevation within targeted tissues (Ter Haar and Coussios 2007; Staruch et al. 2011; Tempany et al. 2011; Rezaeian et al. 2019). It has been successfully applied across a wide range of treatments, including tumor ablation, thrombolysis, kidney stone fragmentation and drug delivery via thermosensitive nanosized drug delivery systems (Dubinsky et al. 2008; Sadeghi-Goughari et al. 2020). When operated in a nondestructive regime, focused ultrasound (FUS) can induce mild HT rather than ablation, allowing selective heating of tumor tissue without harming surrounding healthy regions (Kwan and Coussios 2017). The temperature in the focal region can be precisely controlled to reach approximately 42 °C (Moradi Kashkooli et al. 2023a, b), a range sufficient to trigger the release of encapsulated drugs from TSLs. This temperature-mediated release enhances intratumoral drug accumulation while minimizing systemic toxicity and improving bioavailability (Kwan and Coussios 2017). The FUS-triggered TSL drug delivery approach has shown significant therapeutic potential for treating solid tumors (Dromi et al. 2007; Schroeder et al. 2009; Grüll and Langereis 2012; Ranjan et al. 2012) and has been successfully demonstrated in clinical settings for various cancers, including those of the liver, prostate, breast and kidney (Haar et al. 1989; Madersbacher et al. 1995; Gianfelice et al. 2003a, b; Blana et al. 2004; Kennedy et al. 2004; Illing et al. 2005; Kennedy 2005; Suomi et al. 2018; Sadeghi-Goughari et al. 2020).

Numerous preclinical and clinical studies have demonstrated the effectiveness of combining FUS with TSLs. Preclinical in vivo studies have demonstrated the safety and feasibility of the HIFU + TSL system in treating pancreatic cancer and shown that HIFU-induced mild hyperthermia effectively triggers TSLs to release their payloads, thereby enhancing intratumoral drug accumulation while reducing systemic toxicity (Frenkel et al. 2006; Ponce et al. 2006; Dromi et al. 2007; Treat et al. 2007, 2012; Hagtvet et al. 2011; Staruch et al. 2012; Kheirolomoom et al. 2013; Hijnen et al. 2014, 2017; Centelles et al. 2018; Farr et al. 2018). Furthermore, the safety and efficacy of the combined HIFU + TSL therapeutic modality have been validated in recent clinical trials, which demonstrated enhanced intratumoral drug delivery for targeted treatment of liver tumors (Lyon et al. 2017, 2018, 2019; Gray et al. 2019). Despite promising progress, the mechanisms governing drug transport and tumor uptake in TSL systems under FUS-induced heating remain poorly understood (Moradi Kashkooli et al. 2023a, b). This knowledge gap underscores the need for comprehensive investigations supported by mathematical modeling.

Modeling enables the analysis of heat transfer, drug transport and interactions among the tumor TME, TSLs and chemotherapeutic agents, providing valuable insight for optimizing treatment outcomes. Previous studies have examined various aspects of TSL-based delivery, including the effects of blood temperature (El-Kareh and Secomb 2003), comparisons with conventional chemotherapy and stealth liposomes (Gasselhuber et al. 2012a, b), key parameters affecting intracellular drug concentration (Liu and Xu 2015) and drug distribution within two-dimensional tumor models (Zhang et al. 2009; Gasselhuber et al. 2012a, b). Other works have emphasized the timing between hyperthermia onset and drug administration (Tehrani et al. 2022) and optimized TSL-mediated delivery using experimental cytotoxicity data (Ramajayam et al. 2022). More recently, studies have explored the temperature dependence of parameters related to tumor tissue and therapeutic agents, including TSLs and chemotherapeutic compounds, for specific applications (Zhan and Wang 2018; Rezaeian et al. 2019; Zhan et al. 2019; Kim et al. 2021; Löke et al. 2021; Moradi Kashkooli et al. 2021a, b, c; Soltani et al. 2021; Moradi Kashkooli et al. 2023a, b; Namakshenas and Mojra 2023). However, these mathematical models lack a comprehensive computational framework that accounts for the complexity of the heterogeneous TME, including structural and functional characteristics of the tumor vasculature and stroma components, which are essential for accurately predicting and optimizing drug delivery systems. They also lack a mechanistic integration of the ultrasound-induced drug release kinetics with drug transport and delivery.

To address this gap, our study develops a mathematical framework to investigate the combined effects of nanosized TSL-mediated drug delivery and FUS-induced hyperthermia in solid tumors. The model captures the coupled thermal, mechanical and biological processes governing treatment efficacy. Building on our previous work (Mpekris et al. 2015, Mpekris et al. 2017b, a, Mpekris, Voutouri et al. 2018, Zhao et al. 2019, Mpekris et al. 2020, Koutsi et al. 2025a, b, Koutsi et al. 2025a, b), it incorporates (i) FUS acoustic field, modeled via the Helmholtz wave equation; (ii) tissue temperature distribution using the bio-heat transfer equation (BHTE); and (iii) TSLs’ drug release and transport mechanisms that account for heat-dependent tissue and drug properties. In addition, the model explicitly incorporates the structural and cellular characteristics of the TME, accounting for the heterogeneous distribution of vascular and stromal components, solid stress-induced vessel compression and elevated IFP. More specifically, it captures the complex interactions among tumor and immune cells, as well as tumor vasculature components. Together, these features enable a realistic representation of drug transport barriers and fluid dynamics within the tumor, providing a more physiologically relevant prediction of therapeutic outcomes. Model predictions are validated against published experimental data (Dromi et al. 2007; Hagtvet et al. 2011), confirming the accuracy and reliability of this work. A sensitivity analysis is conducted to identify key parameters that influence treatment performance. These include factors related to FUS application, such as ultrasound frequency and exposure duration, as well as parameters associated with TSLs, including liposome size and drug release rate constant. The analysis highlights how these critical factors affect drug delivery efficiency and guides the optimization of FUS-induced hyperthermia and TSL-mediated therapy. Overall, the novelty of this work lies in the development and validation of a coupled 3D mechanistic tissue-level model that explicitly links FUS-mediated hyperthermia to changes in TME transport properties, such as vascular permeability/porosity and interstitial hydraulic conductivity and quantifies their impact on transvascular transport, intratumoral drug distribution and therapeutic outcome. This framework therefore provides a predictive tool for assessing and optimizing FUS-driven tumor microenvironment modulation of drug delivery and consequently therapeutic efficacy.

## Materials and methods

### Model formulation and description

Tumor growth within the host tissue is modeled in a continuum mechanics framework, employing the multiplicative decomposition of the deformation gradient tensor. The mathematical model is deterministic, and the quasi-static linear momentum balance is solved to determine the equilibrium configuration of the tumor at each time step (Voutouri and Stylianopoulos 2014, Mpekris et al. 2015, Mpekris et al. 2017b, a, Angeli et al. 2018, Mpekris et al. 2018, Zhao et al. 2019, Mpekris et al. 2020). A detailed description of the governing equations, underlying assumptions and theoretical foundations is provided in Supplementary Information (SI) Appendix.

The model includes the complex interactions within the TME among multiple components that collectively contribute to the intricate process of tumor progression. These include diverse populations of: *(i) tumor cells*: non-stem-like cancer cells (*CCs*), stem-like cancer cells (*SCCs*) and treatment-induced cancer cells (*ICCs*) (Mpekris et al. 2020); *(ii) immune cells:* natural killer (*NK*) cells, *CD8*^*+*^* T-cells*, *CD4*^*+*^* T-cells*, regulatory T-cells (*Tregs*) and tumor-associated macrophages (*TAMs*); and *(iii) vascular components:* endothelial cells (*ECs*), angiopoietins (*Ang*) and vascular endothelial growth factor (*VEGF*). The model also incorporates factors related to tumor perfusion, oxygenation and drug delivery, including nanosized TSLs.

**Fig. 1:**
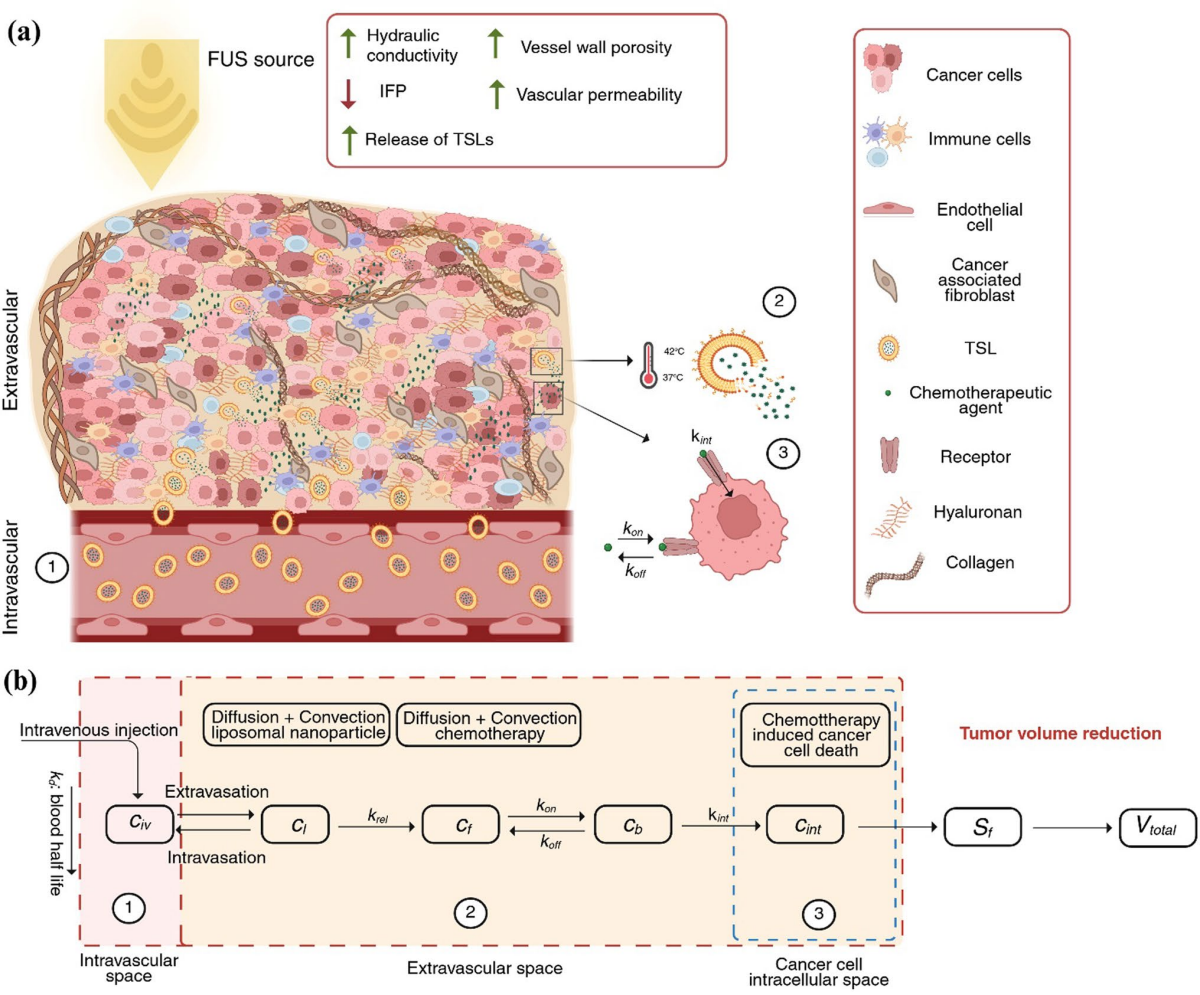
Schematic representation of TSL drug transport from the vascular to the extravascular space of tumors induced by FUS-triggered hyperthermia. **a** Under the propagation of acoustic waves generated by a FUS source, the temperature of the tumor tissue rises. The application of FUS also enhances transvascular flux**,** leading to greater accumulation of TSLs within the tumor tissue. In the model, this increase in the flux arises from two FUS-dependent mechanisms**:** (1) hyperthermia accelerates drug release from nanosized TSLs, elevating the concentration of free drug available in the extracellular space; and (2) FUS enhances hydraulic conductivity, lowers IFP and increases vessel wall porosity and vascular permeability, thereby improving functional vascular density and perfusion. These effects collectively facilitate the transport of both TSLs and released drug into the TME. In the hyperthermic region, the lipid membrane of the TSLs becomes destabilized, triggering the release of the encapsulated chemotherapy. The released drug molecules then bind to receptors on the cancer cell surface and, if not detached, penetrate the cell membrane to enter the cancer cells. **b** In the present computational multicompartment model, drug transport is described across the vascular, extracellular and cancer cells’ intracellular spaces, along with their interactions. Each biophysical region is treated as a distinct compartment governed by convection and diffusion transport mechanisms and coupled with its adjacent compartments (*c*_iv_: vascular concentration of the administered drug, *c*_l_: TSL concentration, c_f_: free drug concentration, *c*_b_: bound drug concentration, *c*_int_: internalized drug, *Q*_sta_: transport of the nanocarrier across the tumor vessel wall and *k*_rel_: drug release rate from the nanocarrier in response to temperature, *k*_on_: binding (association) rate constant, *k*_off_: unbinding (dissociation) rate constant, *k*_int_: internalization rate constant). The internalized drug concentration, *c*_int_, determines the cancer cell survival fraction, *S*_f_ which is linked to total tumor reduction, *V*_total_ through the mass balance equations of cancer cells. TSLs are not shown to scale and are presented for visualization purposes. Created with BioRender.com

The application of FUS increases the effective porosity of the vessel wall, thereby enhancing vascular permeability (Arvanitis et al. 2018). In the fluid phase, FUS exposure leads to a substantial increase in tumor hydraulic conductivity (Arvanitis et al. 2018). These FUS-induced changes in vascular and interstitial transport properties are incorporated as mechanisms in the model. A schematic representation of these mechanisms is provided in Fig. 1a.

In the model, the application of FUS increases the functional vascular density**,** which directly enhances the intratumoral delivery of nanosized TSLs. This increased drug availability leads to more effective killing of cancer cells (Mpekris et al. 2024). The model also incorporates the effect of improved vascular function on oxygen supply, where higher oxygenation levels promote both tumor and immune cell activity. Oxygen concentration additionally modulates the polarization of *TAMs,* shifting from the immunosuppressive *M2* to the immunostimulatory *M1* phenotype (de Pillis et al. 2005; Mpekris et al. 2017b, a; Mahlbacher et al. 2018). However, rapid tumor cell proliferation contributes to solid stress accumulation, which compresses nearby vessels in the model, reducing perfusion and leading to immune cell inactivation within the TME (Griffon-Etienne et al. 1999; Padera et al. 2004). In contrast, immune cell proliferation enhances the tumoricidal response, as *M1-like TAMs* exert cytotoxic effects on tumor cells, whereas *M2-like TAMs* suppress immune effector functions and sustain an immunosuppressive microenvironment (Goel et al. 2011, 2012). The tumor vasculature evolves through angiogenesis, driven by endothelial cell proliferation, which plays a crucial role in maintaining tumor perfusion (Mpekris et al. 2020). Vascular growth is modulated by *VEGF*, which increases *EC *proliferation and promotes *M2-like TAM* accumulation (Carmeliet and Jain 2011). The model also includes the opposing actions of *Ang1* and *Ang2*, where high *Ang2* concentrations destabilize existing vessels by impairing endothelial formation, whereas *Ang1* counteracts this effect by promoting vascular stabilization and *ECs’* support (Holash et al. 1999; Lobov et al. 2002). Fig. S1, SI Appendix presents a schematic diagram illustrating the components of the mathematical model and their interactions, as described above.

### Drug delivery

#### Mechanistic modeling of TSL transport

The spatiotemporal behavior of the thermosensitive liposomal drug delivery system within the tumor is modeled through a set of coupled partial differential equations (PDEs). These equations capture the mechanisms of TSL transport and drug release under FUS-induced hyperthermia. Drug transport across the vascular, extracellular and intracellular spaces is represented through distinct, interacting compartments governed by convection, diffusion and reaction processes. The convection–diffusion–reaction (CDR) equations are solved to quantify the temporal and spatial distribution of drug concentrations in each compartment and can be expressed as (Mpekris et al. 2015; Stylianopoulos et al. 2015, 2018; Moradi Kashkooli et al. 2021a, b, c; Souri et al. 2022; Moradi Kashkooli et al. 2025):

1 $$\underbrace {{\frac{{\partial c_\mathrm{l} }}{{\partial t}}}}_{{{\mathrm{rate}}\,{\mathrm{of}}\,{\mathrm{change}}}} = \,\,\,\underbrace {{D_\mathrm{l} \cdot \nabla ^{2} c_{{\mathrm{l}}} }}_{{{\mathrm{diffusion}}}} - \underbrace {{\nabla \cdot \,(c_\mathrm{l} \mathbf{v}^{f} )}}_{{{\mathrm{convection}}}} - \underbrace {{k_{{{\mathrm{rel}}}} c_\mathrm{l} }}_{{{\mathrm{drug}}\,{\mathrm{release}}}} + \underbrace {{Q_{{{\mathrm{sta}}}} }}_{{{\mathrm{transvascular}}\,{\mathrm{transport}}}}$$

2 $$\underbrace {{\frac{{\partial \mathrm{c}_{\mathrm{f}} }}{{\partial t}}}}_{{{\mathrm{rate}}\,{\text{of change}}}} = \,\,\,\underbrace {{D_{{\mathrm{f}}} \cdot \nabla ^{2} c_{{\mathrm{f}}} }}_{{{\mathrm{diffusion}}}}\;-\;\underbrace {{ \nabla \cdot \,(c_{{\mathrm{f}}} \mathbf{v}^{f} )}}_{{{\mathrm{convection}}}} + \underbrace {{\alpha k_{{{\mathrm{rel}}}} c_{{\mathrm{l}}} }}_{{{\mathrm{drug}}\,{\mathrm{release}}}}\,\, - \underbrace {{\frac{1}{\varphi }k_{{{\mathrm{on}}}} c_{{{\mathrm{rec}}}} \mathrm{c}_{\mathrm{f}} }}_{{{\mathrm{drug}}\,{\mathrm{binding}}\,{\mathrm{to}}\,{\mathrm{receptors}}}} + \underbrace {{k_{{{\mathrm{off}}}} c_{{\mathrm{b}}} }}_{{{\mathrm{drug}}\,{\mathrm{seperating}}\,{\mathrm{from}}\,{\mathrm{receptors}}}}$$

3 $$\underbrace {{\frac{{\partial c_{{\mathrm{b}}} }}{\partial t}}}_{{{\mathrm{rate}}\,{\mathrm{of}}\,{\mathrm{change}}}} = \,\,\,\underbrace {{\frac{1}{\varphi }k_{{{\mathrm{on}}}} c_{{{\mathrm{rec}}}} c_{{\mathrm{f}}} }}_{\begin{subarray}{l} {\mathrm{drug}}\,{\mathrm{binding}}\,{\mathrm{to}} \\ \,\,\,\,\,\,{\mathrm{receptors}} \end{subarray} }\,\,\, - \underbrace {{k_{{{\mathrm{off}}}} c_{{\mathrm{b}}} }}_{\begin{subarray}{l} \,\,\,\,\,\,{\mathrm{drug}}\,{\mathrm{separating}}\, \\ \,\,\,\,\,\,\,{\mathrm{from}}\,{\mathrm{receptors}} \end{subarray} } - \underbrace {{k_{{{\mathrm{int}}}} c_{{\mathrm{b}}} }}_{{\,\,\,{\mathrm{drug}}\,\,{\mathrm{internalization}}}}$$

4 $$\underbrace {{\frac{{\partial c_{{{\mathrm{int}}}} }}{\partial t}}}_{{{\mathrm{rate}}\,{\mathrm{of}}\,{\mathrm{change}}}} = \underbrace {{k_{{{\mathrm{int}}}} c_{{\mathrm{b}}} }}_{{{\mathrm{drug}}\,{\mathrm{internalization}}}}$$

##### Rate of change

Variation of the TSL or chemotherapy concentration over time (*t*). Here, *c*_l_, *c*_f_, *c*_b_ and *c*_int_ denote the concentrations of TSLs, free chemotherapy drug, bound drug and internalized drug, respectively.

##### Diffusion

Diffusive transport of TSLs and chemotherapy. *D*_l_ and *D*_f_ denote the effective diffusion coefficients of the TSL and the free chemotherapeutic drug within the tumor interstitial space, respectively. The diffusion terms describe the rates at which these species are transported through the tumor tissue under the influence of concentration gradients, and are characterized as macroscopic parameters that account for the size of the TSL system and the microarchitecture of the TME. Diffusion coefficients were taken from experimental measurements in the literature (Pluen et al. 2001).

##### Convection

Convective transport of TSLs and chemotherapy within the interstitial fluid flowing through the pores of the TME. It is directly dependent on the interstitial fluid velocity ***v***^*f*^, which is driven by pressure gradients within the tissue. Further details regarding its formulation are provided in SI Appendix, Eqs. (S19–S24). In many solid tumors, the elevated IFP and the dense ECM can substantially hinder or even suppress convective transport (Moradi Kashkooli et al. 2025).

##### Reaction

Physiological and physicochemical processes that contribute to either an increase or a decrease in the concentration of the drug within the tumor tissue. The main processes include transvascular transport (*Q*_sta_); drug release dynamics characterized by the release rate from the nanocarrier in response to temperature (*k*_rel_) (Table S3, SI Appendix) and the number of chemotherapy molecules contained in the nanocarrier (*α*); drug binding to cell surface receptors characterized by the volume fraction of tumor accessible to drugs (*φ*), the binding (association) rate (*c*_rec_); drug detachment from receptors governed by the unbinding (dissociation) rate constant (*k*_off_); and cellular internalization, represented by the internalization rate constant (*k*_int_).

Release rates of the drug from the TSLs as a function of the temperature were modeled using an empirical exponential function of the local tissue temperature (Tagami et al. 2012; Tehrani et al. 2024).

5 $$k_{{{\mathrm{rel}}}} (T_{t} ) = \,4 \times 10^{ - 73} {\mathrm{exp}}(0.5204 \times T_{t} )$$

The experimentally valid range of Eq. (5) is 37–42 °C, and it is therefore applied within this range. The release rate constant *k*_rel_ (s^−1^) at different temperatures is given in Table S1, SI Appendix.

The term *Q*_*sta*_ on the right-hand side of Eq. (1) represents the transport of the TSL nanocarrier across the tumor vessel wall, and it is defined by Starling’s approximation as (Baxter and Jain 1988, 1989; Stylianopoulos and Jain 2013):

6 $$Q_{{{\mathrm{sta}}}} = \,\underbrace {{P_{{{\mathrm{er}}}} S_{{\mathrm{v}}} (c_{{{\mathrm{iv}}}} - c_{{\mathrm{l}}} )}}_{{{\mathrm{diffusion}}}} + \underbrace {{L_{{\mathrm{p}}} S_{{\mathrm{v}}} (p_{{\mathrm{v}}} - p)(1 - \sigma _{{\mathrm{f}}} )\,c_{{{\mathrm{iv}}}} }}_{{{\mathrm{convection}}}}$$

where *P*_er_ is the vascular permeability, *S*_v_ is the vascular density, *p*_v_ is the vascular pressure and* p* is the interstitial fluid pressure. Here, *L*_p_ denotes the hydraulic conductivity of the vessel wall and *c*_iv_ = exp(− (*t *− *t*_0_)/*k*_d_) describes the vascular concentration following a bolus injection, where *t*_0_ is the injection time and *k*_d_ represents the rate of blood circulation decay. The reflection coefficient is denoted by *σ*_f_. The vascular conductivity *L*_p_ depends on the vessel wall pore radius, while the parameters *P*_er_ and *σ*_f_ vary with the ratio of the drug radius to the vessel wall pore radius (Deen 1987). The right-hand side of Eq. (6) captures the two primary mechanisms of transvascular transport: The first term, *P*_er_* S*_v_ (*c*_iv_ – *c*_l_), represents transvascular diffusion, driven by concentration gradients across the vessel wall, and the second term, *L*_p_* S*_v_ (*p*_v_ − *p*)(1 − *σ*_f_) *c*_iv_, represents transvascular convection, driven by pressure differences between the vasculature and the interstitial space. Together, these mechanisms describe the bidirectional exchange of nanocarriers across the blood vessel wall, encompassing transport from the vasculature into the surrounding tissue as well as from the tissue back into the circulation. A schematic representation of the equations governing the aforementioned drug transport is given in Fig. 1(b).

According to the theory of particle transport through cylindrical pores, the hydraulic conductivity (*L*_p_) of the vessel walls, vascular permeability (*P*_er_) and reflection coefficient (*σ*_f_) can be determined by the size of the pores or the relative size of the particle to the size of the pores, using the following relations (Deen 1987):

7 $$L_{{\mathrm{p}}} = \frac{{\gamma r_{0}^{2} }}{{8\eta L_{{{\mathrm{vw}}}} }}$$

8 $$P_{{{\mathrm{er}}}} = \frac{{\gamma HD_{0} }}{{L_{{{\mathrm{vw}}}} }}$$

9 $$\sigma_{\mathrm{f}} = 1 - W$$

in which *γ* denotes the proportion of the surface area that is occupied by pores (vessel wall effective porosity), *r*_0_ represents the pore radius, *η* refers to the viscosity of water at 310 K and *L*_vw_ indicates the thickness of the vessel wall. *D*_0_ is recognized as the diffusion coefficient of a particle in a free solution at 310 K, which can be calculated using the Stokes–Einstein equation as:

10 $$D_{0} = \frac{{K_{{\mathrm{b}}} T_{{{\mathrm{emp}}}} }}{{6\pi \eta r_{\mathrm{s}} }}$$

where *K*_b_ represents the Boltzmann constant, $T_{{{\mathrm{emp}}}}$ denotes the temperature and $r_{\mathrm{s}}$ refers to the radius of the diffusing particle. The parameters *H* and *W* account for hydrodynamic and steric interactions, respectively. For dilute solutions, they can be calculated by (Deen 1987; Stylianopoulos et al. 2015):

11 $$H = \frac{6\pi F}{{K_{t} }}$$

12 $$W = \frac{{F(2 - F)K_{{\mathrm{s}}} }}{{2K_{t} }}$$

*F* is defined as the partition coefficient (Deen 1987):

13 $$F = 1 - \lambda^{2}$$

where *λ* denotes the ratio of the drug particle size to the vessel wall pore size, *λ* = *r*_s_/*r*_0_. The coefficients ${K}_{{s}}$ and ${K}_{{\mathrm{t}}}$ as presented in Eqs. (11–12) are determined by (Deen 1987):

14 $$\left( {\begin{array}{*{20}c} {K_{t} } \\ {K_{{\mathrm{s}}} } \\ \end{array} } \right) = \frac{9}{4}\pi^{2} \sqrt 2 \left( {1 - \lambda } \right)^{ - 5/2} \left[ {1 + \sum\limits_{n = 1}^{2} {\left( {\begin{array}{*{20}c} {\alpha_{n} } \\ {b_{n} } \\ \end{array} } \right)\left( {1 - \lambda } \right)^{n} } } \right] + \sum\limits_{n = 0}^{4} {\left( {\begin{array}{*{20}c} {\alpha_{n + 3} } \\ {b_{n + 3} } \\ \end{array} } \right)\,\,\lambda^{n} }$$

For oxygen, both *γ* and *Η* in Eq. (8) were set to unity, assuming that oxygen diffuses freely through the vessel wall without being impeded by hydrodynamic interactions.

##### Cancer cell killing

Cancer cells are the only model compartments affected by the drug. The fraction of cancer cells (*CCs*, *SCCs* and *ICCs*) surviving after drug exposure is modeled as a function of the internalized drug concentration, c_int_, and is given in Eq. (S13), SI Appendix, along with the corresponding parameter values reported in Table S2, SI Appendix (Liu et al. 2006; Eikenberry 2009). The internalized concentration modulates cytotoxicity through the cancer cell survival fraction (*S*_f_), which enters the cancer cell population balance equations (Eqs. (S9-S11), SI Appendix), reducing net proliferation and thereby producing tumor growth inhibition and regression.

### Modeling the propagation of FUS acoustic waves inducing controlled hyperthermia

The linear propagation of ultrasound waves in a medium during FUS irradiation is analyzed using the time-independent Helmholtz equation, which is solved in the frequency domain (Namakshenas and Mojra 2023; Moradi Kashkooli et al. 2024). This approach is appropriate for a steady-state harmonic acoustic field at a single operating frequency and avoids explicitly resolving the MHz time domain oscillations, while directly providing the complex pressure amplitude required to compute time-averaged acoustic energy deposition (Moradi Kashkooli et al. 2023a, b). In our simulations, the acoustic frequency is *f* = 1 MHz.

15 $$\frac{{(\kappa^{*} )^{2} }}{{\rho_{\mathrm{t0}} }}p_{{1}} +\triangledown \cdot \left[ {\frac{{1}}{{\rho_{t0} }}\left( {\triangledown p_{{1}} } \right)} \right] = \,{0}$$

In this formulation, *p*_1_, *ρ*_t0_ and (κ*)^2^ denote the acoustic pressure, tissue density and squared wave number, respectively. The wave number is defined as κ* = ω*/*c*_0_, where ω* = 2π*f* is the angular frequency, *f* is the operating acoustic frequency and *c*_0_ is the speed of ultrasound in the medium.

The model does not account explicitly for the transducer geometry, but the incident ultrasound field is prescribed analytically using the background pressure field feature (Dey and Shirron 2006; Wei and Weavers 2016; Tiong et al. 2025). This implements a scattered field formulation in which the total acoustic pressure is decomposed into an incident (background) field and a scattered field:

16 $$p_{t} = \,p_{{\mathrm{b}}} + p_{{\mathrm{s}}}$$

where *p*_b_​ is the prescribed background (incident) pressure and *p*_*s*​_ is the computed scattered pressure (dependent variable *p*_1_).

In this study, the acoustic field is specified as a spherical-wave background pressure field (effective point source representation) with source location (***x***_0_*,****y***_0_*,****z***_0_) = (0.12, 0.12, 0.12) m, positioned outside the computational domain. The source strength is defined by the pressure amplitude *p*_0_ = 5.9 × 10^3^ Pa, specified at the reference distance *r*_ref_ = 1 m from the point source (Comsol 2024). For a spherical wave, the pressure amplitude decreases with distance from the source. Therefore, specifying only *p*_0​_ is not sufficient unless the distance at which *p*_0​_ is defined is also stated. The choice of *r*_ref_ = 1 m is a standard convention used to define the source strength. The value of *p*_0​_ was selected (calibrated) to achieve the target hyperthermia temperature in the tumor region (e.g., peak ≈ 41 °C) under the specified exposure duration and thermal boundary conditions. This approach creates an analytic incident wave into the domain without requiring explicit transducer meshing and is used here to model absorption-driven hyperthermia for subsequent thermal-drug coupling.

For a spherical-wave background field, the prescribed incident pressure takes the form (Wei and Weavers 2016; Comsol 2024):

17 $$p_{\mathrm{b}} = \,p_{0} \frac{r_{\mathrm{ref}}}{r_{\mathrm{s}}}e^{ - {\mathrm{ik}}^{*} r_{\mathrm{s}}} \,r_{\mathrm{ref}} = 1\;{\mathrm{m}}\;\;\;\;\;r_{s} = \vert {x} \, - \, {x_{0}}  \vert $$

where *p*_0_ is the amplitude given at the reference distance of 1 m, *r*_s_ is the distance from the source, ***x***_*0*_ is the source location of the spherical wave and ***x*** denotes any point inside the computational domain where the background pressure is evaluated.

In order to couple the acoustic pressure field with the temperature field, it is necessary to estimate the thermal energy deposition resulting from the absorption of ultrasonic waves. The following equation (Nyborg 1986) quantifies the ultrasonic power deposition per unit volume (Zhan et al. 2019):

18 $$Q_{{{\mathrm{ex}}}} = \,2\alpha_{t} I = 2\alpha_{t} \left( {\frac{{p_{1}^{2} }}{{2\rho_{t0} c_{0} }}} \right) = \alpha_{t} \left( {\frac{{p_{1}^{2} }}{{\rho_{t0} c_{0} }}} \right)$$

where *α*_t_ is a frequency-dependent acoustic absorption coefficient and *I* is the ultrasound acoustic intensity.

The acoustic intensity is computed from the complex pressure amplitude as:

19 $$I = \frac{{\left| {p_{1} } \right|\,^{2} }}{{2\rho_{t0} c_{0} }}$$

The resulting volumetric power deposition *Q*_ex_ is used as the thermal source term in the bio-heat equation.

The absorption coefficient increases by the frequency enhancement and could be calculated by (Solovchuk et al. 2013; Zhan 2014; Namakshenas and Mojra 2019; Moradi Kashkooli et al. 2023a, b):

20 $$\alpha_{t} = \alpha_{0} \left( {\frac{f}{{f_\mathrm{0} }}} \right)^{{\eta \,^{*} }}$$

where *α*_0_ is the absorption coefficient at *f*_0_ = 1 MHz. In biological soft tissues, the absorption coefficient varies linearly with frequency (Solovchuk et al. 2013), leading to a coefficient value of η* = 1.

FUS propagation also influences several parameters of the TME, such as the effective porosity of the vessel wall (*γ*) and the hydraulic conductivity (*k*_th_) of the tumor tissue (Arvanitis et al. 2018). Specifically, the hydraulic conductivity increases approximately 5 times, while the effective vessel wall porosity increases by a factor of 1.5 (Arvanitis et al. 2018). In the present model, these FUS-induced effects are incorporated by applying the corresponding changes to the relevant parameters, which are assumed to persist for 24 h following sonication (Moradi Kashkooli et al. 2023a, b), after which the parameters return to their baseline values.

Mathematically, for a parameter *par* ∈ {*γ*, *k*_th_}, we apply a steplike post-FUS modification:

21 $${\mathrm{par}}\,(t) = \left\{ {\begin{array}{*{20}c} {{\mathrm{par}}_{0} ,\,\,\,\,t < t_{{{\mathrm{FUS}}}} \,\,} \\ {a_{\mathrm{p}} \,{\mathrm{par}}_{0} ,\,\,\,t_{{{\mathrm{FUS}}}} \le \,t < t_{{{\mathrm{FUS}}}} + 24\,{\mathrm{h}}\,\,} \\ {{\mathrm{par}}_{0} ,\,\,\,\,t \ge t_{{{\mathrm{FUS}}}} + 24\,{\mathrm{h}}} \\ \end{array} } \right.$$

Here, *par*_0_ denotes the baseline (pre-FUS) value (i.e., *k*_th_,_0_ for *k*_th_ and *γ*_0_ for *γ*), *t*_FUS_ is the sonication start time and *α*_p_ = 5 and 1.5 are the multiplicative factors for hydraulic conductivity and vessel wall porosity, respectively.

### Temperature distribution predicted by the bio-heat transfer equation (BHTE)

The spatial distribution of temperature within the tumor and surrounding normal tissues is governed by the bio-heat transfer equation (BHTE) (Bird 2015, Moradi Kashkooli et al. 2023a, b, Tehrani et al. 2024):

22 $$\underbrace {{\rho_{t} c_{t} \frac{{\partial T_{t} }}{\partial t}}}_{{{\mathrm{rate}}\,{\mathrm{of}}\,{\mathrm{change}}}} = \,\underbrace {{k_{t} \nabla^{2} T_{t} }}_{{{\mathrm{conduction}}}}\,\, + \underbrace {{Q_{m} }}_{\begin{subarray}{l} {\mathrm{heat}}\,{\mathrm{generated}}\,{\mathrm{by}}\,{\mathrm{metabolism}}\, \\ {\mathrm{at}}\,{\mathrm{the}}\,{\mathrm{reference}}\,{\mathrm{temperature}} \end{subarray} } + \underbrace {{Q_{{{\mathrm{ex}}}} }}_{\begin{subarray}{l} {\mathrm{external}}\,{\mathrm{power}} \\ \,\,\,\,\,\,\,{\mathrm{deposition}} \end{subarray} }$$

where *T*_t_ is the absolute temperature. The parameters *ρ*_t0_, *c*_t0_ and *k*_t0_ are the density, the specific heat capacity and the thermal conductivity of the tumor and normal tissue. The parameter *Q*_m_ represents the value of metabolic heat in the tumor and host tissue, which can be neglected compared to the heat generated by hyperthermia. *Q*_ex_ is the source term of heat absorbed from external power applied to warm the tissue and describes how much of the acoustic energy is converted into heat per unit volume. It is important to note that heating triggers drug release (*k*_rel_) as a result of the tissue’s response to elevated temperature (Moradi Kashkooli et al. 2023a, b; Moradi Kashkooli et al. 2024; Bhandari et al. 2025). The thermosensitive liposomal nanoparticle drug delivery system is designed to remain stable at normal body temperature and to release its payload when the local temperature, calculated using Pennes’ BHTE, reaches the pre-designed threshold of 42–43 °C (Haemmerich et al. 2023; Moradi Kashkooli et al. 2024).

In the model, the temperature increase induced by ultrasound not only triggers the release of TSLs but also influences the physical properties considered in this framework, including the density**,** specific heat capacity and thermal conductivity of the tissue (Tan et al. 2018, Zou et al. 2020, Moradi Kashkooli et al. 2023a, b). Equations (19–21) describe this phenomenon for each parameter:

Density (*ρ*_t_)

23 $$\begin{aligned} & \rho_{t0} - 2.97434 \cdot T_{t} + 0.0042 \cdot T_{t}^{2} + 0.00293 \cdot T_{t}^{3} \\ & \quad - 6.14447e - 5 \cdot T_{t}^{4} + 3.33019e - 7 \cdot T_{t}^{5} \\ \end{aligned}$$

Specific heat capacity (*c*_t_)

24 $$\begin{aligned} & c_{t0} + 53.55552 \cdot T_{t} - 3.96009 \cdot T_{t}^{2} + 0.10084 \cdot T_{t}^{3} \\ & \quad - 0.00106 \cdot T_{t}^{4} + 4.01666e - 6 \cdot T_{t}^{5} \\ \end{aligned}$$

Thermal conductivity (*k*_t_)

25 $$\begin{aligned} & k_{t0} - 0.02094 \cdot T_{t} + 3.89971e - 4 \cdot T_{t}^{2} - 5.47541e - 7 \cdot T_{t}^{3} \\ & \quad - 4.14455e - 8 \cdot T_{t}^{4} + 2.97188e - 10 \cdot T_{t}^{5} \\ \end{aligned}$$

The detailed description of the model and pertinent equations can be found in Eqs. (S1-S35), SI Appendix. The values of all model parameters, including their units and references, are summarized in Tables S2-S3. The initial conditions of the variables at *t* = 0 day are provided in Table S4, SI Appendix.

### Solution strategy

To simplify the simulations, the tumor is assumed to possess a spherical geometry embedded within a healthy host tissue of cubic shape. The cubic host medium, which defines the spatial framework for tumor expansion, is configured to be two orders of magnitude larger than the tumor to minimize boundary-induced effects that could influence tumor evolution. Making the assumption of symmetry to reduce computational demands, only one-eighth of the domain is considered for numerical analysis (Kim et al. 2011; Mpekris et al. 2017b, a). All boundary conditions for this mathematical framework are provided in Eqs. (S36-S56), SI Appendix, while Fig. S2, SI Appendix illustrates the computational domain geometry and indicates the locations where the boundary conditions are applied.

The model equations were solved using the commercial finite element software COMSOL Multiphysics (COMSOL, Inc., Burlington, MA, USA). The computational mesh consisted of 6,015 free tetrahedral finite elements and 51,628 degrees of freedom. The finite element formulation employed quadratic Lagrange shape functions. Time integration was carried out via the backward differentiation formula (BDF) scheme using the PARDISO solver and setting a time step of 0.25 d. The time-stepping approach was configured as Free*,* enabling the solver to automatically adjust the time increments within this limit. To ensure timescale consistency**,** we adopted a piecewise multirate strategy**:** Tumor growth and long-term transport properties were integrated on a day-scale frame, whereas the application of sonication and drug administration were resolved on a dedicated treatment window**.** Specifically, during FUS application (which occurs over a few minutes), the time step size was reduced to 9 s, allowing the solver to take smaller steps while preventing overly coarse increments during the heating and temperature-triggered release phase. Following the treatment window, the simulation returned to the day-scale time integration up to the end of the study. Mesh and time-step independence were assessed by repeating the simulations on a refined spatial discretization and with a reduced time step size. The procedure and corresponding results are provided in Supporting Information and are summarized in Table S5, SI Appendix. In addition, a treatment window adjustment was also conducted by reducing the time step size during sonication from 9 to 4 s (30 min sonication). Results are reported in Supporting Information and summarized in Table S6, indicating negligible changes in peak temperature and released drug.

## Results

### Validation of the computational model

**Fig. 2:**
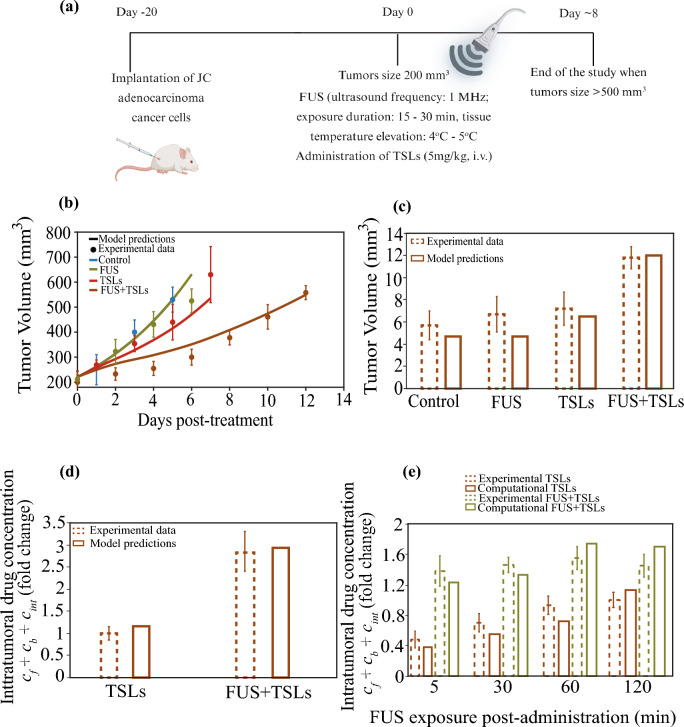
Comparison between model predictions and experimental data for tumor growth and intratumoral drug concentration (*c*_f_ + *c*_b_ + *c*_int_) in JC adenocarcinoma tumors (Dromi et al. 2007). Drug concentrations are presented as fold change relative to the TSL group. **a** Experimental treatment protocol applied to JC adenocarcinoma tumors and replicated in the model simulations. Created with BioRender.com. **b** Tumor growth of murine mammary JC adenocarcinoma cells (dots) compared with model predictions (solid lines) for each treatment group. Model performance was evaluated using the coefficient of determination (*R*^2^) and the Root Mean Squared Error (RMSE), with nRMSE reported as RMSE normalized by the mean experimental tumor volume (expressed as a percentage). Control: *R*^2^ = 0.98, RMSE = 17.93 mm^3^, nRMSE = 5.20%; FUS: *R*^2^ = 0.80, RMSE = 54.66 mm^3^, nRMSE = 14.78%; TSLs: *R*^2^ = 0.90, RMSE = 46.15 mm^3^, nRMSE = 12.19%; FUS + TSLs: *R*^2^ = 0.92, RMSE = 34.67 mm^3^, nRMSE = 10.12%. **c** Bar graph showing the number of days post-treatment required for tumors to reach a volume of 500 mm^3^ under the four treatment protocols, with brown and green bars representing experimental data and model predictions, respectively. **d** Intratumoral drug concentration for TSLs with and without FUS under the no-delay condition (FUS initiated immediately after TSL administration). **e** Intratumoral drug concentration as a function of injection to exposure lag time (5–120 min) for TSL and FUS + TSL groups. Error bars denote standard error of the mean (SE) for experimental data (*n* = 5). For simulations, (*c*_f_ + *c*_b_ + *c*_int_) in the FUS + TSL group was evaluated at the end of the FUS exposure, while TSL group values were evaluated at matched post-injection times

The model’s predictive performance and parameter values’ robustness were evaluated by comparing model predictions with measurements from well-designed published, in vivo data (Dromi et al. 2007; Hagtvet et al. 2011). An in vivo study using murine mammary JC adenocarcinoma tumors demonstrated that FUS exposures in combination with TSLs led to a more rapid release and significantly higher intratumoral accumulation of chemotherapy doxorubicin compared to TSLs alone (Dromi et al. 2007). Moreover, the combination of FUS + TSLs resulted in a significantly greater inhibition of tumor growth relative to all other treatment groups (Dromi et al. 2007). We set up the model to replicate the experimental protocol employed in the experimental study (Fig. 2a). In the experiments, mice were randomized into four groups: control, FUS (frequency: 1 MHz; exposure duration: 15–30 min; tissue temperature elevation: 4–5 °C), TSLs and FUS + TSLs. The treatment regimen began once the tumor volume reached ~ 200 mm^3^, at which point TSLs were administered intravenously, considered as day 0, and pulsed FUS was applied immediately after. Mice were euthanized when tumor volumes reached 500 mm^3^, and the number of days required to reach this size was recorded and presented as the group mean in Fig. 2(b, c).

Model predictions show good agreement with experimental measurements (Dromi et al. 2007) of intratumoral drug concentration as shown in Figs. 2(d–e). In our model, the intratumoral drug concentration is defined as the sum of the free, bound and internalized drug within the tumor (*c*_f_ + *c*_b_ + *c*_int_). To facilitate direct comparison between simulations and in vivo measurements, drug concentrations are reported as fold change relative to the TSL treatment group. Fig. 2d summarizes the effect of combining FUS + TSLs under the condition of no intentional delay between TSL administration and FUS exposure. In both the experimental data and model predictions, the combined FUS + TSL treatment yields a marked enhancement in intratumoral drug delivery, with approximately a 3–3.5-fold increase in intratumoral drug concentration relative to TSLs alone. Fig. 2e presents intratumoral drug concentration ​ as a function of the injection to exposure lag time (5–120 min) for both treatment groups. For the TSL group the intratumoral drug concentration increases with time, consistent with continued accumulation of drug in the tumor. In contrast, for the FUS + TSL group, varying the lag time over 5–120 min produces only modest changes in intratumoral drug concentration​​, indicating that the overall enhancement achieved by FUS-triggered delivery is comparatively insensitive to the precise exposure initiation time within this window. In the simulations, to model the experimental protocol, drug concentration for the FUS + TSL group was evaluated at the end of the FUS exposure, while for the TSL group values were evaluated at matched post-injection times.

**Fig. 3:**
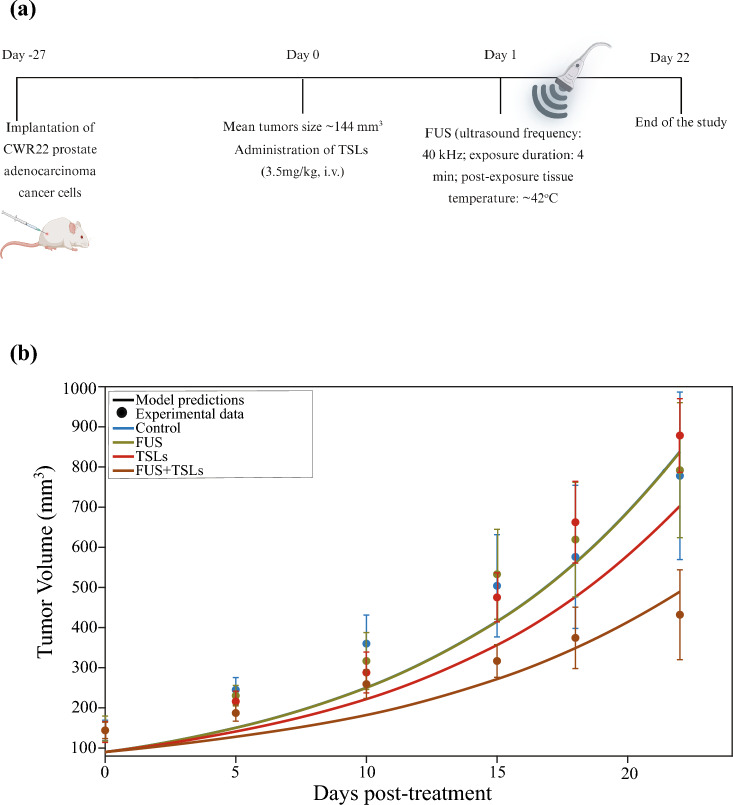
Comparison between model predictions and experimental data for tumor growth in CWR22 prostate adenocarcinoma tumors (Hagtvet et al. 2011). **a** Experimental treatment protocol applied to CWR22 prostate adenocarcinoma tumors and simulated by the model. Created with BioRender.com. **b** Tumor growth curves of murine CWR22 prostate adenocarcinoma tumors (dots) compared with mathematical model predictions (solid lines) for each treatment group. For all cases (control, FUS, TSLs and FUS + TSLs), model performance was evaluated using the coefficient of determination (*R*^2^) and the Root Mean Squared Error (RMSE), with nRMSE reported as RMSE normalized by the mean experimental tumor volume (expressed as a percentage). Control: *R*^2^ = 0.87, RMSE = 76.90 mm^3^, nRMSE** = **17.70%; FUS: *R*^2^ = 0.89, RMSE = 74.28 mm^3^, nRMSE = 16.91%; TSLs: *R*^2^ = 0.77, RMSE = 124.15 mm^3^, nRMSE** = **27.96%; FUS + TSLs: *R*^2^ = 0.70, RMSE = 55.29 mm^3^, nRMSE = 19.36%

Additionally, data from another in vivo study using CWR22 prostate adenocarcinoma tumors were utilized to validate the model (Hagtvet et al. 2011). The results demonstrated that the TSL formulation alone exhibited no inhibitory effect on tumor growth. However, when combined with FUS, a significant inhibition of tumor growth was observed (Hagtvet et al. 2011). We formulated the model to simulate this experimental protocol and compare its predictions with these experimental data **(**Fig. 3a**)**. In the experimental setup, mice were randomized into four groups: control, FUS (frequency: 40 kHz; exposure duration: 4 min; post-exposure tissue temperature: 42 °C), TSLs (3.5 mg/kg, intravenous) and FUS + TSLs. The treatment regimen began once the tumor volume reached ~ 144 mm^3^, at which point TSLs were administered intravenously. FUS exposure was applied 24 h post-injection, coinciding with the peak intratumoral TSL concentration. (Hagtvet et al. 2011).

To enable a direct comparison between the model predictions and experimental observations, all model parameters were assigned baseline values according to the literature (Tables S2-S3, SI Appendix). The only parameter adjusted to align the model predictions with experimental results was the parameter denoted as *k*_*1*_, which quantifies the relationship between cancer cell proliferation and oxygen concentration (SI Appendix, Equation S8). The value of *k*_*1*_ was determined by fitting the tumor growth data of the untreated (control) group and was subsequently held constant across all treatments within the same study. Specifically, *k*_*1*_ was estimated as 0.51 day^−1^ for JC adenocarcinoma cancer cells (Dromi et al. 2007) and 0.38 day^−1^ for CWR22 prostate adenocarcinoma cancer cells (Hagtvet et al. 2011) (Table S7, SI Appendix). An optimization algorithm (Harkos et al. 2023, Hadjigeorgiou et al. 2025b) was employed to minimize the error between experimental data and model predictions. Since each tumor cell line exhibits distinct growth dynamics, calibrating *k*_*1​*_ for each tumor type ensures that the model accurately captures the biological variability in tumor proliferation rates.

The tumor volume predictions showed strong agreement with experimental data. Monotherapies—namely control, FUS, TSLs—did not exhibit any significant antitumor effects relative to the control group. In contrast, the combination therapies (FUS + TSLs) produced a substantial reduction in tumor volume, confirming the synergistic therapeutic effect of focused ultrasound and drug delivery systems (Figs. 2b and 3b). The predictive accuracy of the mathematical model was assessed using the coefficient of determination (R^2^), which quantifies the correlation between experimental measurements and model predictions, ranging from 0 to 1, with values close to 1 indicating excellent agreement. In addition to R^2^, the Root Mean Squared Error (RMSE) was computed to quantify the typical magnitude of the deviation between model-predicted and experimentally measured tumor volumes in absolute units (mm^3^). We further report the normalized RMSE (nRMSE), which expresses RMSE as a percentage of the mean experimental tumor volume at a given time point and provides an interpretable relative measure of model error. Finally, the bar graph **(**Fig. 2c) presents the number of days required for tumors to reach a volume of 500 mm^3^ under four treatment conditions: control, FUS alone, TSLs alone and the combination of FUS + TSLs. The model agreed well with the in vivo data showing that the combination of FUS + TSLs resulted in the most significant delay in tumor progression.

### Sensitivity analysis

A sensitivity analysis was performed to investigate the influence of key parameters in the model. A comprehensive global sensitivity analysis of the principal parameters related to tumor growth modeling has been previously conducted (Sotiropoulos 2025). However, that study did not consider the effects of FUS-induced hyperthermia or the TSLs. Therefore, in the present work, a local sensitivity analysis was carried out based on the coupled FUS-induced hyperthermia and temperature-triggered TSL drug delivery simulations, to evaluate parameters specifically associated with the FUS application, such as the ultrasound frequency (*f*) and exposure time (*t*ₑₓₚ), and parameters related to the thermosensitive liposomal nanoparticles, including their release rate (*k*_rel_) and particle size (*r*_s_).

#### Effect of FUS parameters

Upon the application of FUS, there is an enhancement in vessel wall porosity and, consequently, in vascular permeability, along with a marked increase in the hydraulic conductivity of the tumor (Arvanitis et al. 2018). The frequency of ultrasound used in TSL-based drug delivery systems typically ranges from several kHz to a few MHz. In general, high-frequency modalities between 1 and 5 MHz are employed to achieve thermal ablation or localized hyperthermia (Joshi and Joshi 2019; Moradi Kashkooli et al. 2023a, b). Moreover, the exposure duration of FUS plays a critical role in optimizing therapeutic efficacy. In applications aimed at enhancing TSL-mediated drug release within the tumor microenvironment, exposure times commonly range from a few seconds to several minutes (Moradi Kashkooli et al. 2023a, b; Moradi Kashkooli et al. 2024, 2025).

**Fig. 4:**
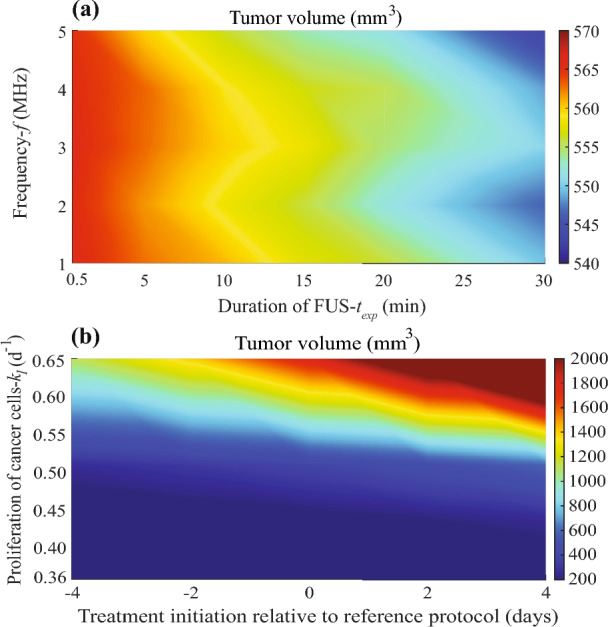
Phase diagrams illustrating the effects of key therapeutic parameters on tumor response. **a** Effect of FUS duration and frequency on tumor volume following FUS + TSL therapy. **b** Influence of the cancer cell proliferation rate constant and the timing of combined therapy (FUS + TSLs) administration on tumor volume. Both panels demonstrate how variations in treatment parameters, tumor growth kinetics and therapy scheduling affect the overall therapeutic outcome. Day 0 corresponds to the reference protocol start, and negative/positive values denote earlier/delayed initiation relative to that time; the corresponding tumor volume at initiation depends on *k*_*1*_​

The effect of key therapeutic parameters on tumor response following the combined application of FUS and TSLs is depicted in Fig. 4. In Fig. 4a, the influence of FUS frequency (*f*) and exposure duration (*t*ₑₓₚ) on the resulting tumor volume is presented. The simulation results reveal that the smallest tumor volume is achieved when the ultrasound frequency ranges between 4 and 5 MHz and the exposure duration is between 20 and 30 min. It is evident that the exposure duration exerts a more pronounced effect on tumor reduction compared to the frequency. Even at lower frequencies (approximately 2 MHz), an extended exposure duration yields a notable reduction in tumor volume. These results indicate that within the explored range and under the present model configuration, exposure duration has a stronger influence on treatment efficacy than ultrasound frequency. Accordingly, frequency appears to play a secondary role in this range of parameter values. However, because frequency can modulate heating distributions and bioeffects, its impact may be more pronounced under different operating conditions or outside the tested range. In Fig. 4b, the impact of cancer cell proliferation rate constant (*k*_*1*_) and the timing of the combined FUS + TSL treatment relative to the experimental protocol were analyzed. The horizontal axis reports treatment initiation time relative to the baseline experimental protocol: Day 0 corresponds to the standard protocol start (reference initiation time), negative offsets (− 2, − 4 days) indicate earlier initiation (2 and 4 days before the reference time; smaller tumor size/earlier growth stage) and positive offsets (+ 2, + 4 days) indicate delayed initiation (2 and 4 days after the reference time; larger tumor size/later growth stage). Because tumor growth kinetics vary with *k*_*1*_​, the absolute tumor volume at initiation differs across *k*_*1*_​ for the same offset; thus, timing is reported relative to the reference protocol start. Table S8, SI Appendix shows tumor volume at treatment initiation and normalized growth stage ratio for each proliferation rate *k*_*1*​_ and timing shift relative to the reference protocol (day 0). The results demonstrate that, at lower proliferation rates (*k*_*1*_ < 0.55 d^−1^), tumor volume reduction remains relatively stable regardless of treatment timing, indicating that slow-growing tumors are less sensitive to the schedule of the therapy. At higher proliferation rates (*k*_*1*_ > 0.55 d^−1^), early application of the combined treatment produces markedly improved outcomes, with a pronounced decrease in tumor volume when therapy is initiated prior to the reference time. This finding highlights the importance of synchronizing FUS + TSL therapy with the tumor’s proliferative dynamics. Consistent behavior is also observed in supplementary simulations (Fig. S3, SI Appendix), where, particularly for intermediate proliferation rates (0.48–0.51 d^−1^), early treatment initiation substantially enhances the therapeutic response and accelerates tumor regression.

To provide additional insights for the parametric analysis of frequency and exposure duration (*t*_exp_) in Fig. 4a, the corresponding temperature evolution and spatial heating patterns for the two extreme exposure durations are shown in Supplementary Information. Specifically, Fig. S4, SI Appendix presents intratumoral temperature profiles for ultrasound frequencies *f* = 1–5 MHz for the shortest (*t*_exp_ = 0.5 min) and longest (*t*_exp_ = 30 min) sonication times, including the post-sonication cooling phase. In both cases, increasing frequency yields higher intratumoral temperatures. For *t*_exp_ = 0.5 min (Fig. S4(a)), heating is brief and peak temperature remains limited (maximum ≈40.65 °C at *f* = 5 MHz), followed by relatively fast cooling toward the baseline value. For *t*_exp_ = 30 min (Fig. S4(b)), prolonged sonication produces higher peak temperatures (≈41.2 °C at *f* = 1 MHz up to ≈44.5 °C at *f* = 5 MHz) and a slower decay after ultrasound cessation, consistent with greater accumulated heating. Complementarily, Fig. S5, SI Appendix shows radial temperature profiles at the end of sonication for the same frequency range and exposure durations. Temperature is higher within the tumor and decreases across the tumor–host tissue interface, with a steeper gradient outside the tumor. Compared to the short-exposure case (Fig. S5(a)), the long exposure (Fig. S5(b)) results in a more pronounced temperature elevation and a broader heated region, illustrating how exposure time modulates both peak heating and spatial heat spread.

**Fig. 5:**
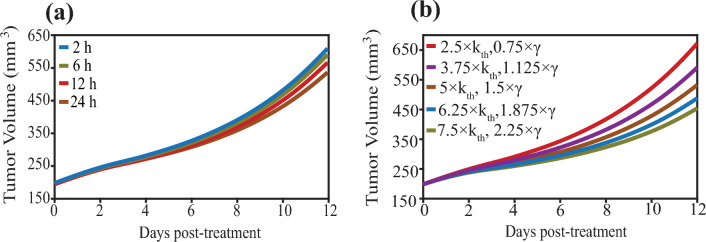
Tumor response to the duration and magnitude of FUS-induced transport changes**. a** Tumor volume response for different persistence times of the FUS-induced increases in tumor hydraulic conductivity (*k*_th_) and vessel wall effective porosity (*γ*). The magnitudes were fixed to the reference FUS-induced enhancement factors used throughout this work (*k*_th_ = 5× , *γ* = 1.5×), while the duration was varied (2, 6, 12 and 24 h post-sonication). **b** Tumor volume sensitivity to the magnitude of the FUS-induced changes in *k*_*th*_ and *γ*. The persistence time was fixed (24 h), while *k*_th_ and *γ* multipliers were varied around baseline (*k*_th_ = 5 × , γ = 1.5 ×) by ± 25% and ± 50% (i.e., *k*_th_ = 3.75 × − 6.25 × and 2.5 × − 7.5 × ; *γ* = 1.125 × -1.875 × and 0.75 × -2.25 ×)

The therapeutic response in this mathematical framework is strongly influenced by the transient FUS-mediated enhancement of vascular and interstitial transport. Therefore, we performed an additional sensitivity analysis varying the effect of FUS on tumor hydraulic conductivity (*k*_th_) and vessel wall effective porosity (*γ*), which modulate transvascular flux and interstitial transport. The magnitudes were fixed to the reference FUS-induced enhancement factors used throughout this work. FUS was assumed to increase *k*_th_ by × 5 and *γ* by × 1.5 for 24 h following sonication (Arvanitis et al. 2018; Moradi Kashkooli et al. 2023a, b). First, in Fig. 5a, we varied the persistence time of these FUS-induced changes (2, 6, 12 and 24 h) while keeping their magnitudes fixed at the referenced values. Shortening the persistence time systematically attenuated treatment efficacy: The greatest tumor volume reduction was obtained when the effects persisted for 24 h, followed by 12 h, 6 h and 2 h. Quantitatively, the final tumor volume decreased from 601.5 mm^3^ (2 h) to 532.8 mm^3^ (24 h), corresponding to an 11.4% reduction in final volume when the FUS effects persisted for 24 h compared to 2 h. Overall, the separation between these curves is small over time, indicating only a limited sensitivity to persistence duration within the 2–24 h range. Second, in Fig. 5b we examined magnitude dependence by varying the multipliers of *k*_th_ and *γ* around baseline (± 25% and ± 50%), while keeping the persistence time fixed (24 h). Tumor response exhibited a monotonic trend: Larger increases in *k*_th_ and* γ* led to greater tumor volume reduction, whereas smaller increases diminished efficacy. Specifically, the final tumor volume was 454.1 mm^3^ for the strongest enhancement (*k*_th_ = 7.5× , *γ* = 2.25×) compared with 672.2 mm^3^ for the weakest enhancement (*k*_th_** = **2.5× , γ** = **0.75×), representing a 32.4% reduction under stronger transport enhancement. Overall, this analysis demonstrates that the predicted tumor volume is sensitive to both the persistence time and the magnitude of the FUS-induced transport changes, with longer lasting and larger enhancements in *k*_*th*_ and *γ* yielding improved therapeutic efficacy.

#### Effect of TSL parameters

**Fig. 6:**
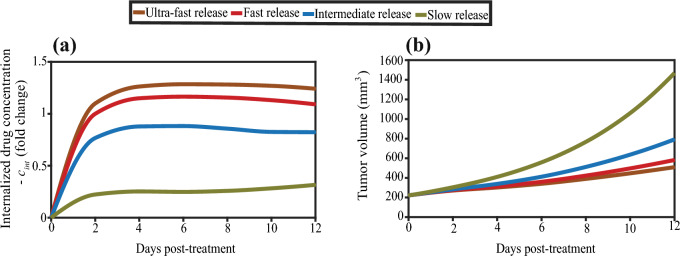
Effect of drug release rates (ultra-fast, fast, intermediate and slow) on **a** the internalized drug concentration (*c*_int_) within the tumor and **b** tumor volume. In panel **(a)**, internalized drug concentrations are reported as fold changes normalized to the value associated with the fast release formulation at day 2 post-treatment

In Fig. 6, we analyzed the impact of different TSL drug release rates on therapeutic outcomes. As reported in the literature (Tagami et al. 2012) certain TSL formulations allow gradual drug release at physiological temperature (37 °C), while exhibiting significantly faster release within mild hyperthermia (~ 42 °C) (Moradi Kashkooli et al. 2020, Haemmerich et al. 2023). The release rate constants across temperatures ranging from 37 to 42 °C are summarized in Table S9, SI Appendix, for ultra-fast, fast, intermediate and slow release formulations and the corresponding fitted curves and fitting equations are shown in Fig. S6, SI Appendix. Among these, the fast release formulation was selected as the baseline for this study (Table S1, SI Appendix) (Tagami et al. 2012, Tehrani et al. 2024). The effect of varying release rates (ultra-fast, fast, intermediate and slow) on the internalized drug concentration (*c*_int_) within the tumor is demonstrated in Fig. 6a. The results suggest that ultra-fast release rates yield the highest internalized drug concentration, followed by fast, intermediate and slow release formulations. Internalized drug concentrations are expressed as fold changes relative to the value of the fast release rate at day 2 post-treatment. Figure 6b presents the corresponding evolution of tumor volume under each release condition. The results clearly show that faster release kinetics lead to improved therapeutic outcomes, as indicated by smaller tumor volumes. Specifically, tumors treated with ultra-fast releasing TSLs exhibit the greatest reduction in volume, followed by the fast, intermediate and slow release groups. These findings emphasize the critical role of release kinetics in optimizing TSL-mediated drug delivery and therapeutic efficacy.

**Fig. 7:**
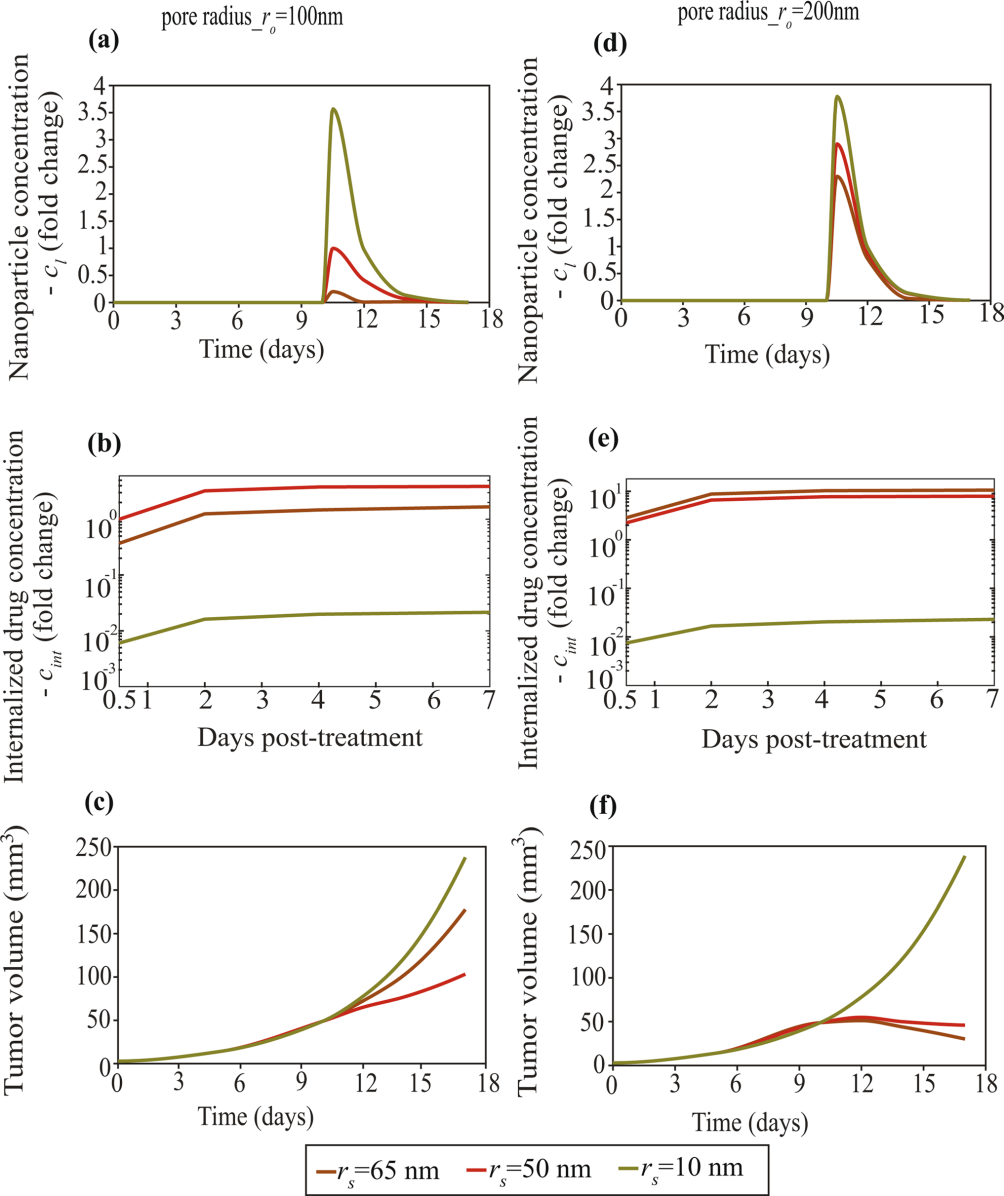
Effect of the radius of diffusing thermosensitive liposomal nanoparticles on therapeutic outcomes. Panels **a–c** illustrate the temporal evolution of the nanoparticle concentration (cₗ), internalized drug concentration (*c*_int_) and tumor volume, for a blood vessel pore radius (*r*_0_) of 100 nm, while panels **d–f** depict the corresponding results for  (*r*_0_)= 200 nm. Nanoparticle concentrations are presented as fold changes normalized to the 50 nm nanoparticle case at day 10 (day 0 post-treatment), and internalized drug concentrations are expressed as fold changes normalized to the corresponding value at day 10.5 (day 0.5 post-treatment)

Next, we set out to investigate the effect of TSLs’ size on therapeutic efficacy under different vascular permeability conditions (Fig. 7) accounting for tumors with moderately permeable and hyperpermeable vessels. Panels (a–c) show the temporal evolution of the nanoparticle concentration (cₗ), internalized drug concentration (*c*_int_) and tumor volume for vessel wall pore radius r₀ (*r*_0_) of 100 nm, whereas panels (d–f) present the corresponding results for *r₀* = 200 nm. Three nanoparticle sizes were examined with radii: *r*_s_ = 10 nm, 50 nm and 65 nm. Different-sized particles were also taken to have different diffusion coefficients and drug payloads based on the literature (Table S10, SI Appendix). The nanoparticle with *r*ₛ = 50 nm served as the baseline case.

For vessel wall pore sizes of 100 nm, the smallest nanoparticle (*rₛ* = 10 nm) exhibits the highest interstitial concentration, peaking at approximately 3.6 times higher than the baseline, owing to its small size and high diffusivity (Fig. 7a). In contrast, the largest nanoparticle ( *r*_s_= 65 nm) exhibits the lowest interstitial concentration, remaining below 0.5 times the baseline, as its large size restricts diffusion across the vessel wall. The internalized drug concentration (Fig. 7b) follows a different trend, with  *r*_s_= 50 nm yielding the highest c_int_, attributed to its optimal combination of drug payload and transvascular transport. The *r*_s_= 65 nm also achieves a high internalized concentration, though slightly lower than that of *r*_s_= 50 nm. The  *r*_s_= 10 nm case, despite higher diffusivity, exhibits a lower intracellular accumulation (**~ **10^–twofold^) due to its lower drug load. Consequently, tumor growth (Fig. 7c) mirrors these results:  *r*_s_= 50 nm produces the most pronounced regression in tumor volume, with the final tumor volume remaining below 120 mm^3^, while for *r*_s_ = 65 nm and *r*_s_= 10 nm, the final tumor volumes reach approximately 180 mm^3^ and 250 mm^3^, respectively.

The therapeutic response of the nanoparticles is affected in large part by the particle size (*r*_s_) and payload (*α*). For that reason, we repeated the simulations fixing the payload across various particle sizes to decouple transport-driven effects from drug loading effects. Specifically, for moderate vascular permeability (*r*_0_ = 100 nm), we repeated simulations for *r*_s_ = 10, 50 and 65 nm while keeping the payload constant (a = 10^4^, i.e., the baseline payload used for *r*_s_ = 50 nm in Table S10, SI Appendix). The resulting trends are shown in Fig. S7. Holding *α* constant increases c_int_ for *r*_s_ = 10 nm (Fig. S7(b)) compared to Fig. 7b, leading to stronger tumor growth inhibition and the smallest final tumor volume (Fig. S7(c)). For *r*_s_ = 65 nm, fixing *α* decreases *c*_int_ because its payload is reduced, resulting in weaker tumor control and the largest final tumor volume (Fig. S7(c)).

For larger vessel wall pores (*r*_0_ = 200 nm, Fig. 7d–f), the enhanced permeability significantly boosts extravasation of nanoparticles, resulting in higher extracellular (cₗ) and intracellular (*c*_int_) concentrations for all nanoparticle sizes compared to *r*_0_** = **100 nm pores. In Fig. 7d, the absolute peak interstitial nanoparticle concentration is highest for the smallest particle size (* r*_s_ = 10 nm). However, the largest increase relative to the *r*_0_ = 100 nm case (Fig. 7a) is observed for * r*_s_ = 65 nm, whose nanoparticle concentration rises from 0.2 to 2.3 times the baseline, indicating that larger pores facilitate the transvascular transport of the larger nanoparticles. These changes lead to a marked reduction in tumor volume (Fig. 7f), with* r*_s_ = 65 nm achieving the strongest therapeutic response, followed by *r*_s_ = 50 nm, while * r*_s_ = 10 nm remains the least effective, yielding the largest final tumor size. Nanoparticle concentrations are expressed as fold changes relative to the* r*_s_ = 50 nm value at day 10 (day 0 post-treatment), and internalized drug concentrations are expressed as fold changes relative to the * r*_s_ = 50 nm value at day 10.5 (day 0.5 post-treatment).

#### Effect of tumor mechanical stiffness

The mechanical characteristics of the TME also play a crucial role in regulating tumor progression and therapeutic response. Another parameter that affects tumor growth is the mechanical properties of the tumor with respect to the host tissue (Voutouri et al. 2014). We investigated how different values of the tumor elastic modulus (*E*) affect the key variables of the mathematical model (Figs. S8-S9, SI Appendix). Specifically, we used values of the tumor elastic modulus: *E* = 30 kPa, 40 kPa and 60 kPa (Voutouri et al. 2021b, Mpekris et al. 2023; Voutouri et al. 2023; Charalambous et al. 2024; Kalli et al. 2024; Mpekris et al. 2024, Neophytou et al. 2025a). Fig. S8 shows that as tumor stiffness increases, the bulk stress (Fig. S8(a)) becomes more pronounced, leading to greater compression of the tumor vasculature and, consequently, a reduction in the functional vascular density (Fig. S8(b)). This vascular collapse results in reduced oxygen supply, reflected in lower oxygen concentration within stiffer tumors (Fig. S8(c)). Not only oxygen supply but also drug delivery is affected by increased tumor stiffness. Therefore, a similar trend is observed in Fig. S9. In stiffer tumors (*E* = 60 kPa), the concentration of internalized drug (Fig. S9(a)) is the lowest due to impaired vascular function and restricted drug transport, leading to reduced therapeutic efficacy. Consequently, these tumors exhibit the largest final volumes (Fig. S9(b)). In contrast, tumors with lower stiffness (*E* = 30 kPa and *E* = 40 kPa) show enhanced drug internalization and greater tumor shrinkage. These findings highlight that reducing tumor stiffness improves vascular function, oxygenation and drug delivery, thereby enhancing the overall therapeutic efficacy of the combined FUS–TSL treatment.

#### Quantitative assessment of the sensitivity of key model parameters

This subsection aims to quantitatively assess how variations in key model parameters influence tumor growth predictions, thereby identifying the most critical factors governing model behavior and treatment response. In this study, we performed a single-parameter variation sensitivity analysis, in which each parameter was varied independently across three discrete levels while all other parameters were held fixed**.** In Table 2, key parameters of the mathematical model and their effects on the model outputs are analyzed using this approach. The logarithm of the total variance in tumor volume (log(Tot. Var.) > *1*) quantifies the degree to which each parameter affects the model predictions. The analysis was conducted on five parameters: the release rate of the chemotherapeutic agent from thermosensitive liposomes (*k*_rel_), the radius of the diffusing thermosensitive liposomal nanoparticles (*r*_s_), the tumor cell proliferation rate (*k*_*1*_), the frequency of focused ultrasound (*f*) and the FUS exposure duration (*t*_exp_). Each parameter was varied at three levels (Table 1) relative to its baseline value, as summarized in Tables S2-S3 of SI Appendix:

**Table 1:** Parameter variations relative to their baseline values. Each parameter is varied across the indicated multipliers to assess model sensitivity and the influence of individual parameters on system behavior

Parameter	Values (relative to baseline)
*k*_rel_	0.1 × , 1 × , 6 ×
*r*_*s*_	0.2 × , 1 × , 1.3 ×
*k*_*1*_	1 × , 1.2 × , 1.4 ×
*f*	1 × , 3 × , 5 ×
*t*_exp_	0.033 × , 1 × , 2 ×

The selected range of parameter values was chosen to reflect values commonly used in experiments and reported in the literature. Frequency and exposure duration were varied within ranges reported for localized hyperthermia applications and TSL-triggered release studies (Joshi and Joshi 2019; Moradi Kashkooli et al. 2023a, b; Moradi Kashkooli et al. 2024, 2025). The release rate multipliers reflect the spread between slow, fast and ultra-fast formulations reported experimentally across 37–42 °C (Tagami et al. 2012; Moradi Kashkooli et al. 2020, Haemmerich et al. 2023). Also, particle sizes were selected to represent typical nanoformulations with associated changes in diffusion and payload (Table S10, SI Appendix). The proliferation rate constant was varied over a narrower range because small deviations produce pronounced changes in tumor dynamics.

For each parameter, the total variance in tumor volume was computed using the following expression:

26 $${\mathrm{Tot}}{\mathrm{.Var}}{.} = \sum\limits_{i}^{3} {\,\,\frac{1}{T}} \,\,\,\int\limits_{0}^{T} {\,\,\left( {V_{i} \left( t \right) - \overline{V} \left( t \right)} \right)}^{2} \,{\mathrm{d}}t$$

where the integration is performed over time t for ith level of each parameter, *T* is the time interval of integration, *V*_*i*_ (*t*) is the tumor volume of the ith level at current time t and $\overline{V }$*(t*) represents the mean tumor volume across all levels. The summation gives the total variance of the three levels. The total variance was evaluated from Day 10 (treatment onset) to Day 17, the period during which the most significant therapeutic effects are observed in the model.

As shown in Table 2, the *k*_*1*_ parameter, representing the proliferation rate of cancer cells, exhibits the highest sensitivity. Even small variations in *k*_*1*_ result in substantial differences in tumor growth, highlighting its dominant influence on tumor dynamics. For this reason, *k*_*1*_ was not varied as extensively as the other parameters during the sensitivity analysis since minor perturbations already produce pronounced effects. In contrast, the FUS frequency (*f*) has a clear physical significance, directly related to experimental conditions. Experimental studies commonly vary the ultrasound frequency three to five times, a rationale also adopted here. Although larger relative variations were applied to *f*, they did not substantially alter the tumor evolution compared with *k*_*1*_ variations. Overall, in the present single-parameter variation sensitivity analysis, the results confirm that *k*_*1*_ exerts the strongest impact on the model, as it directly governs tumor proliferation. Parameters related to nanoparticle properties, such as *k*_rel_ and *r*_s_, also influence model behavior but to a lesser extent. Conversely, parameters associated with FUS application—specifically frequency (*f*) and exposure duration (*t*_exp_)—show lower sensitivity, indicating that the model is less dependent on the precise ultrasound operating conditions. It should be emphasized that this finding does not exclude a potentially stronger influence of frequency in other frequency windows, heating regimes or model configurations.

**Table 2:** Logarithm of total variance in tumor volume for the parameters of the single-parameter variation sensitivity analysis (log(Tot. Var.) > 1)

Parameter	log (Tot.Var.)
*k*_rel_	2.74
*r*_s_	3.41
*k*_*1*_	4.60
*f*	1.11
*t*_exp_	1.13

## Discussion

The present study introduced a comprehensive multiphysics model developed to elucidate the coupled mechanisms governing FUS-induced hyperthermia and TSL-mediated drug delivery in solid tumors. The primary objective was to establish a quantitative framework capable of predicting the interplay among acoustic, thermal and transport phenomena within the TME that collectively determine therapeutic efficacy. The model integrated acoustic propagation, bio-heat transfer and temperature-responsive drug release with key biological processes, including vascular transport, diffusion and cellular uptake. By incorporating these interactions, the model enhances the understanding of how ultrasound exposure parameters and nanocarrier design synergistically influence treatment outcomes.

Given the large number of TME components included, it is also important to clarify which modeled processes most strongly affect the reported outcomes. The framework has been developed over recent years and builds on our previously published and validated models, in which individual components were calibrated against experimental data and evidence from the literature. Specifically, the governing equations for immune cells, including *NK* cells,* CD8*^*+*^ *T-cells*, *CD4*^*+*^ *T-cells*, *Tregs* and tumor-associated macrophages of the *M1* and *M2* phenotypes, have been validated in our prior works (Mpekris et al. 2017b, a, Voutouri et al. 2018, Mpekris et al. 2020, Voutouri et al. 2021a, b, Mpekris et al. 2022, Harkos et al. 2023, Harkos and Stylianopoulos 2024, Hadjigeorgiou et al. 2025a, b). Likewise, angiogenic factors and vascular components, including *ECs*, *VEGF* and *Angs*, as well as cancer cell subpopulations, including *SCCs* and *ICCs*, have been incorporated and validated within the same integrated framework (Mpekris et al. 2017b, a, Voutouri et al. 2018, Mpekris et al. 2020, Voutouri et al. 2021a, b, Mpekris et al. 2022, Hadjigeorgiou and Stylianopoulos 2024). The stromal and vascular compartments are included to provide a realistic baseline tumor microenvironment and to preserve consistency with the validated mathematical model. In the untreated simulations, in which all the aforementioned components are included and immune cells are present, the tumor continues to grow. This indicates that tumor reduction in the treated groups is driven primarily by FUS-induced hyperthermia combined with TSL-mediated mechanisms, rather than by immune-mediated clearance. Accordingly, over the short time window examined here, the chemotherapeutic release rate from thermosensitive liposomes, changes in frequency and exposure duration are among the most immediate and influential determinants of therapeutic efficacy.

Model validation against two independent in vivo datasets (Dromi et al. 2007; Hagtvet et al. 2011) demonstrated strong predictive performance, with coefficients of determination (*R*^2^) exceeding 0.8 in most cases. In both the JC mammary and CWR22 prostate adenocarcinoma models, the combined application of FUS and TSLs produced substantially greater tumor volume reduction than either monotherapy, accurately reproducing the experimentally observed synergistic effects. The model’s ability to capture tumor growth dynamics across distinct tumor types highlights its robustness and broad applicability. Notably, the high *R*^2^ values obtained for the combination therapy groups in both cancer cell lines reflect the model’s strong predictive fidelity. In parallel with *R*^2^, model accuracy was quantified using the root mean square error (RMSE) and the normalized RMSE (nRMSE), providing absolute (mm^3^) and relative (%) measures of deviation between predicted and measured tumor volumes. Across treatment groups, RMSE and nRMSE values were generally low, indicating that the model captures not only the overall trends but also the magnitude of tumor growth dynamics within experimental variability. In addition to tumor volume, the model was also validated against experimental measurements of intratumoral drug concentration (*c*_f_ + *c*_b_ + *c*_int_) in the JC adenocarcinoma model (Dromi et al. 2007), capturing the reported ~ 3–3.5-fold increase in drug accumulation for FUS + TSLs relative to TSLs alone. Model outputs that are not directly compared with experimental data, such as intratumoral temperature rise, release kinetics and vascular permeability-related variables, are therefore treated as predictive and interpreted accordingly.

To achieve this level of agreement between simulation and experiment, only a single parameter—the cancer cell proliferation rate constant (*k*_*1*_)—was adjusted, while all others remained fixed and only for the control groups. This strategy was intended to assess whether modifying a single biological parameter could reproduce general treatment trends across different therapeutic conditions, thereby testing the model’s robustness and generalizability without case-specific calibration. Although this simplification may constrain quantitative precision, the model effectively reproduced relative behaviors and response patterns in most treatment scenarios. Overall, these results demonstrate that a unified modeling framework can yield qualitatively consistent and mechanistically meaningful predictions with minimal parameter adjustment.

The sensitivity analysis provided further insight into the relative influence of key therapeutic and biophysical parameters. Among FUS-related factors, the exposure duration (*t*_exp_) had a stronger effect on tumor reduction than frequency (*f*), suggesting that the cumulative thermal dose, rather than the acoustic frequency itself, is the primary driver of treatment efficacy. Optimal tumor control was achieved for frequencies between 2 and 5 MHz and exposure times of 20–30 min. Importantly, this finding is clinically meaningful because frequency selection is often guided by target depth: Higher frequencies (~ 3 MHz) are typically used for superficial tumors, whereas lower frequencies (~ 1 MHz) are employed for deeper targets due to improved penetration (Malinet 2023). Within the 2–5 MHz range explored here, the comparatively weaker sensitivity to frequency indicates that increasing frequency alone is unlikely to yield substantial additional benefit when thermal delivery is constrained. Instead, our results suggest that, provided safety limits on temperature rise and surrounding tissue exposure are respected, extending the FUS exposure duration may be more effective for enhancing therapeutic response than further increasing frequency. In this framework, frequency primarily serves to balance depth of energy deposition and focal localization, while exposure time governs the accumulated thermal dose that ultimately drives tumor control in the modeled setting. The timing of therapy also emerged as critical: Early administration yielded markedly improved outcomes in fast-growing tumors (*k*_*1*_ > 0.55 d^−1^), underscoring the importance of synchronizing treatment with tumor proliferative dynamics.

TSLs are engineered to rapidly release their encapsulated drug upon heating. The actual release kinetics depend on several factors, including the lipid composition, preparation method and heating temperature (Tagami et al. 2011). To ensure therapeutic efficacy, the release rate must remain minimal under physiological conditions to prevent premature leakage, while achieving rapid and complete release near the lipid phase transition temperature (39–42 °C) (Needham and Dewhirst 2001; Gasselhuber et al. 2012a, b). In this context, our modeling results further demonstrated that the efficiency of TSL-mediated therapy strongly depends on the release kinetics, which govern drug availability within the TME. Under the considered conditions, diffusion dominates over convection as the primary transport mechanism near the tumor center. A detailed transport regime characterization based on representative Péclet numbers is provided in Supporting Information (Eq. S58). Ultra-fast release formulations achieved the highest intracellular drug concentrations and the greatest tumor shrinkage, confirming that rapid and heat-triggered drug release near the lipid phase transition temperature (39–42 °C) is essential for maximizing efficacy. Similarly, nanoparticle size (r_s_) strongly influenced drug transport. At low vascular permeability (r_0_= 100 nm), intermediate-sized TSLs (r_s_ = 50 nm) provided an optimal balance between diffusivity and payload capacity, whereas at higher vascular permeability (r_0_ = 200 nm), larger nanoparticles (r_s_ = 65 nm) became more effective due to improved extravasation and higher drug loading; however, the predicted optimal TSL size is conditional on the coupled size payload (*α*) and transport assumptions, as shown by the fixed payload control analysis, where transport-driven effects dominate and alter the efficacy ranking.

Sensitivity indices confirmed that tumor cell proliferation rate (*k*_*1*_) is the most influential model parameter, followed by the drug release rate (*k*_rel_) and liposome radius (*r*_s_), while the FUS parameters (*f* and *t*_exp_) exhibited lower sensitivity. Although *k*_*1*_ ranks as the most influential parameter because it sets the baseline tumor growth timescale, it is calibrated only to the untreated (control) group for each dataset and is then held constant across all treatment groups. The improved outcomes for the combined FUS + TSL therapy therefore arise from the coupled transport mechanisms explicitly activated by FUS and hyperthermia in the model, namely temperature-triggered drug release (via *k*_rel​_) and FUS-induced changes in vascular/interstitial transport properties (e.g., vessel wall porosity/permeability and hydraulic conductivity), which together increase intratumoral drug exposure and drive tumor response, rather than from re-tuning growth kinetics. Accordingly, the conclusions are expected to hold qualitatively across tumor phenotypes, although the magnitude of response might vary with baseline growth kinetics and vascular transport properties.

Moreover, analysis of tumor mechanical stiffness (*E*) revealed that increasing stiffness leads to elevated solid stress, vascular compression and reduced oxygenation, which together impair drug transport and therapeutic response. Conversely, softer tumors exhibited enhanced perfusion, higher drug uptake and greater regression with treatment. These findings highlight the central role of the tumor’s biophysical environment in shaping therapeutic outcomes and suggest that strategies to reduce stiffness such as stroma normalization (Chauhan et al. 2013, Mpekris et al. 2017b, a, Papageorgis et al. 2017, Panagi et al. 2020, Mpekris et al. 2021, Voutouri et al. 2021b, Panagi et al. 2022, Mpekris et al. 2023, Charalambous et al. 2024, Kalli et al. 2024, Panagi et al. 2024, Dwairy et al. 2025, Neophytou et al. 2025a) could substantially improve the effectiveness of FUS + TSL therapy. Importantly, the above findings should be interpreted in the context of stiff, highly fibrotic tumors, where benefits from stiffness reduction are expected to be the most pronounced, while the impact should differ for less stiff tumors, as is often observed in clinical practice (Panagi et al. 2024).

Despite the agreement between model predictions and experimental data, certain limitations should be recognized. First, the framework assumes spatially uniform tissue properties, omits vessel-level heterogeneity and treats FUS-induced transport enhancements as spatially uniform and temporally prescribed. Introducing discrete vascular networks could improve spatial resolution and enhance the accuracy of local drug distribution predictions. Furthermore, integrating image-derived tumor geometries that reflect the heterogeneous microvascular architecture would yield a more physiologically realistic depiction of solid tumors. Such refinements would enable a deeper exploration of how vascular structures modulate the interplay between TSL delivery and hyperthermia. Image-based modeling could also pave the way for patient-specific simulations that capture individual tumor characteristics, thereby supporting the development of personalized therapeutic strategies (Moradi Kashkooli et al. 2019, Moradi Kashkooli et al. 2021a, b, c, Tehrani et al. 2024).

The current model focuses on the thermally mediated effects of FUS, which is the primary mechanism governing TSL release in this mathematical framework. Consequently, we do not include nonthermal mechanical mechanisms, such as acoustic radiation force (ARF), radiation pressure, acoustic streaming and microbubble cavitation, which can modulate vascular and cellular permeability and thereby affect drug transport (Mitragotri 2005; Moradi Kashkooli et al. 2023a, b). Nonthermal mechanical effects are most relevant in low-intensity pulsed ultrasound (LIPUS), where ARF and acoustic streaming are thought to contribute to targeted drug release from nanoparticle carriers (Hornsby et al. 2021, Hornsby et al. 2022). In contrast, cavitation is dominant at higher acoustic pressures (Zhou et al. 2014; Hornsby et al. 2023) and can be divided into stable or inertial cavitation (Plesset and Prosperetti 1977; Frenkel 2008). Both non-inertial and inertial cavitation can generate shear stresses that may alter or disrupt the nanocarrier structure, leading to drug release (Chen et al. 2019). Cavitation can also disrupt endothelial membranes and increase cell membrane permeabilization, thereby improving drug delivery (Snipstad et al. 2018). Accordingly, cavitation can enhance intratumoral drug concentrations, especially when using liposomal-loaded microbubbles (MBs) or nanobubbles (NBs) (Moradi Kashkooli et al. 2023a, b; Mpekris et al. 2024; Koutsi et al. 2025a, b; Neophytou et al. 2025a, b). Overall, ultrasound can produce both thermal and nonthermal effects and their relative contribution can be tuned by adjusting exposure parameters. Thermal effects are appropriate for TSL-based delivery, whereas cavitation effects are desirable for MB-based delivery. With TSLs, FUS is used to induce hyperthermia at a targeted site, characterized by a temperature rise to about 40–45 °C over 30 min or longer; as TSLs pass through the heated area, the increased temperature causes their destabilization and drug release from these liposomal nanoparticles (NPs) (Moradi Kashkooli et al. 2023a, b). Since our model captures only this thermal pathway, the inclusion of cavitation would be expected to further increase intratumoral drug uptake by enhancing permeability and transport across the vasculature and by promoting additional mechanically induced release from carriers (Snipstad et al. 2018; Chen et al. 2019). Furthermore, loading MBs/NBs with nanoparticles could be an appealing approach for simultaneously inducing drug release and increasing cellular permeability during cavitation, potentially minimizing off-target effects (Moradi Kashkooli et al. 2023a, b).

An additional limitation of the present framework is the use of a 1/8 domain under an idealized geometric and physical symmetry assumption, whereby all modeled components, including the FUS field, are imposed in a symmetry-consistent manner. While this formulation substantially reduces computational cost and enables efficient multiphysics simulations, it may not fully capture asymmetric spatial variations in the FUS exposure conditions. Another limitation is that the transducer geometry was not modeled explicitly. Instead, we represented the ultrasound source by prescribing an analytic incident pressure field (background pressure field) to drive wave propagation in the tissue domain. This simplification is sufficient for predicting absorption-driven hyperthermia and its downstream effects in our coupled framework (Wei and Weavers 2016; Tiong et al. 2025), but it cannot capture transducer-specific near-field features, beam shaping or focal spot characteristics that depend on aperture and curvature. Also, the model does not utilize fully three-dimensional, patient-specific tumor geometries reconstructed from magnetic resonance (MR) or other imaging modality. Incorporation of such data would allow for detailed assessment of the TME, nanocarrier properties and infusion parameters that govern liposome-based therapies. Implementing 3D, image-based geometries could therefore improve the translational and predictive value of our computational framework. In addition, the model does not account for ECM remodeling, because a clearly established biological mechanism linking FUS-induced hyperthermia to ECM alteration is still lacking in the literature. However, experimental evidence suggests that ultrasound exposure can indirectly affect ECM structure. Previous studies have shown that pulsed FUS combined with microbubbles can enhance doxorubicin uptake in pancreatic tumor models through collagen fiber disruption, while others have reported reduced collagen density and improved nanoparticle penetration in lung carcinoma following pulsed FUS exposure (Li et al. 2015; Lee et al. 2017). Although these findings indicate a potential relationship between FUS and ECM remodeling, the underlying mechanisms remain insufficiently characterized. Future developments of this model will aim to incorporate these effects once a clearer mechanistic understanding becomes available.

Finally, tumor tissue was modeled as a poroelastic medium (Roose et al. 2003; Ambrosi et al. 2017; Blanco et al. 2023), where the solid matrix is treated as purely elastic and interstitial fluid transport follows Darcy’s law. This assumption is justified by the temporal and spatial scales of interest, which focus on the slow, macroscale proliferation of cancer cells. While it is well established that tumor tissues can exhibit viscous behavior—particularly at the microscale—we have chosen to omit viscoelastic effects in the present model to avoid further complexity.

In summary, while the aforementioned limitations introduce certain simplifications, they are expected to influence the model’s outcomes primarily in a quantitative rather than qualitative manner. The fundamental mechanistic insights and predictive trends identified, namely the synergistic interplay between FUS-induced hyperthermia, thermosensitive liposome-mediated drug delivery and the tumor’s biophysical environment, remain robust. The proposed model provides a comprehensive mechanistic framework that unifies acoustic, thermal and transport processes within the tumor microenvironment, enabling quantitative prediction of therapeutic response under combined focused ultrasound and thermosensitive liposome treatment. Overall, the model represents a computational framework for analyzing and optimizing ultrasound-assisted nanotherapies, offering a coherent understanding of how physical and biological factors interact to determine therapeutic outcomes and guide the rational design of effective treatment strategies in solid tumors.

## Supplementary Information

Below is the link to the electronic supplementary material.

Supplementary file1 (DOCX 4639 kb)

## Data Availability

The datasets analyzed for this study can be found in the Zenodo. 10.5281/zenodo.18657347
