# Supplementary material for: A multiphysics computational model of focused ultrasound-enhanced drug delivery using temperature-sensitive liposomes
Source: Biomech Model Mechanobiol. 2026 Jun 3;25(3):60. doi: 10.1007/s10237-026-02075-5 (PMC13233647; doi:10.1007/s10237-026-02075-5)
Supplement: Supplementary file 1 — Supplementary file1 (DOCX 4639 kb) [file 10237_2026_2075_MOESM1_ESM.docx]

*Supplementary Information*

**A multiphysics computational model of focused ultrasound-enhanced drug delivery using temperature-sensitive liposomes**

1. Description of the mathematical model

Kinematic principles underlying tumor growth

Equations (S1-S3) and (S20-S28) establish the comprehensive framework for the biomechanical model of tumor growth, which is solved to simulate the progression of the tumor within normal/host tissue. The solutions to these equations facilitate the computation of interstitial fluid pressure, fluid velocity, and the stresses generated through mechanical interactions between the tumor and the host tissue. Interstitial fluid pressure and fluid velocity are incorporated into the drug transport equations (Equations 1-4, in the main text), while the mechanical interactions between the tumor and the host tissue influence the tumor's growth rate. Equations (S4-S17) serve to clarify the balance law concerning the various cell types within the tumor to measure the growth stretch ratio, λ*_g_*, which determines the rate of tumor proliferation. This growth stretch ratio is utilized within the general framework via Equations (S2, S3, and S25). All parameter values of this mathematical model are detailed in Tables S1 and S2. This mathematical framework employs a robust systems-biology approach, which is meticulously designed to incorporate the intricate cellular and subcellular phenomena that occur within biological systems, specifically utilizing a continuum mechanics and finite elements model to thoroughly analyze the multifaceted dynamics of tumor growth. Also, Table S5 illustrates the initial values of the variables used in the mathematical model at time t=0 day.

In order to describe the kinematic behavior of the tumor, the multiplicative decomposition of the deformation gradient tensor is utilized. The tensor $F$ is divided into two distinct components: the tumor's growth, denoted as $F_{g}$, and the elastic mechanical interaction, represented as $F_{e}$ (Rodriguez, Hoger et al. 1994, Skalak, Zargaryan et al. 1996, Ambrosi and Mollica 2002, Mpekris, Angeli et al. 2015):

| $F=F_{e} F_{g}$ | (S1) |
| --- | --- |

Henceforth, all tensor representations will be rendered in boldface to facilitate their distinction from other terms. It is important to recognize that, given the assumptions inherent in the model and the specific problem under investigation, the deformation gradient tensor can be expressed as a combination of more than two independent components, which separately encompass growth, residual stress formation, or extracellular matrix (ECM) remodeling (Skalak, Zargaryan et al. 1996, Stylianopoulos and Jain 2013, Mascheroni, Carfagna et al. 2018). The growth component, denoted as $F_{g}$, has been characterized as isotropic (its properties are the same in all directions) and homogeneous (it is uniform in composition and structure throughout the entire volume), representing a non-stress inducing deformation gradient tensor that represents the proliferation of cancer cells (Roose, Netti et al. 2003, Kim, Stolarska et al. 2011, Stylianopoulos, Martin et al. 2013, Pirentis, Polydorou et al. 2015).

|  | (S2) |
| --- | --- |

where λ*_g_* is the growth stretch ratio, which assesses the proliferation of cancer cells, while **I** represents the second-order identity tensor, indicating that the proliferation associated with tumor growth occurs uniformly and isotropically. The elastic component, $F_{e}$, accounts for the stress-inducing elastic interactions both within the tumor and with the surrounding host tissue. The elastic component of $F$ was derived directly from Equation (S1) as:

|  | (S3) |
| --- | --- |

**Calculation of the growth stretch ratio**

The rate of tumor growth is measured by taking into account both the concentration of oxygen and the proliferation of cancer cells (Roose, Netti et al. 2003, Kim, Stolarska et al. 2011, MacLaurin, Chapman et al. 2012, Voutouri and Stylianopoulos 2014, Mpekris, Voutouri et al. 2018). The equation utilized is:

| $\frac{d \lambda_{g}}{d t}=\frac{1}{3}(\frac{T_{CCs}}{T_{tot}}S_{CCs}+\frac{T_{SCCs}}{T_{tot}}S_{SCCs}+\frac{T_{ICCs}}{T_{tot}}S_{ICCs}) \lambda_{g}$ | (S4) |
| --- | --- |

where $T_{CCs}$ represents the population of non-stem-like cancer cells (CCs), $T_{SCCs}$denotes the stem-like cancer cells (SCCs) population, $T_{ICCs}$signifies the treatment-induced cancer cells (ICCs) population, $T_{tot}$constitutes the total density of tumor cells as determined by the summation of the aforementioned three populations. The $S_{CCs}$, $S_{SCCs}$and $S_{ICCs}$are the respective rates of proliferation and degradation for the CCs, SCCs and ICCs, as specified subsequently in Equations (S9-S11).

**Components of the tumor microenvironment**

As outlined in the current mathematical framework, we incorporate the interactions that occur among cancer cells, immune system cells, and tumor-associated macrophages, which will be elaborated upon subsequently.

**Cancer cells**

The dynamics of the populations of non-stem-like cancer cells (CCs), stem-like cancer cells (SCCs), and treatment-induced cancer cells (ICCs) are delineated by Equations (S5-S7). SCCs exhibit resistance to therapeutic agents, hypoxic conditions, and the immune response, whereas ICCs, upon the administration of therapy, adopt a more stem-like phenotype.

| $\frac{\partial T_{CCs}}{\partial t}=\nabla\cdot(D_{CCs}\nabla T_{CCs})+GS_{f} T_{CCs}-cNT_{CCs}-D+p_{CT} T_{SCCs}+p_{IT} T_{ICCs}-\left(p_{TC}+p_{TI}\right)T_{CCs}-\lambda_{M1} M_{1} T_{CCs}$ | (S5) |
| --- | --- |
|  | (S6) |
| $\frac{\partial T_{ICCs}}{\partial t}=\nabla\cdot(\mathrm{D}_{ICCs}\nabla T_{ICCs})+\alpha_{ICCs}GS_{f_{ICCs}}T_{ICCs}-c_{ICCs} N T_{ICCs}-D_{ICCs}+p_{TI} T_{CCs}+p_{CI} T_{SCCs}-\left(p_{IT}+p_{IC}\right)T_{ICCs}$ | (S7) |

In this context, *N* represents the Natural Killer (NK) cells, M*_1_* corresponds to the M*_1_* tumor-associated macrophages (TAMs), while D*_CCs_*, D*_SCCs_*, and D*_ICCs_* denote the diffusion coefficients of non-stem-like, stem-like, and treatment-induced cancer cells, respectively. To incorporate the influence of drug delivery on growth, the surviving fractions of cancer cells, denoted as *S_f_*, $S_{f_{SCCs}}$ and $S_{f_{ICCs}}$, are integrated into Equations (S5), (S6), and (S7), respectively. The variables *c*,$c_{SCCs}$, $c_{ICCs}$ and *D*, *D_SCCs_*, *D_ICCs_* quantify the respective proportions of tumor cells that are eliminated by NK and CD8^+^ T-cells. Especially, for the parameters $c_{SCCs}$, *D_SCCs_*, $c_{ICCs}$ and *D_ICCs_*, which characterize the cytotoxic potential of immune cells against SCCs and ICCs, we argue that these cells display heightened resistance during interactions with immune entities. Based on empirical evidence (Todaro, D'Asaro et al. 2009), the cytotoxic efficacy of CD8^+^ T-cells against stem cancer cells (SCCs) is considered to be seven times lower than that against CCs.  Therefore, the parameters that govern the elimination of SCCs by immune cells are assumed to be the same as for CCs but multiplied by a factor of 0.14.

*G* characterizes the proliferation of CCs, SCCs, and ICCs as a function of oxygen availability.  Regarding the coefficients of the proliferation rates for SCCs and ICCs, namely $\alpha_{SCCs}$ and $\alpha_{ICCs}$, we claim that under normal oxygen conditions, these values are equivalent to one, thereby ensuring that all cancer cell types exhibit proliferation rates consistent with those of CCs. Conversely, under hypoxic conditions, the proliferation rates of cancer cells exhibiting a stem-like phenotype are observed to increase. Consequently, we hypothesize that their proliferation rates are inversely proportional to the oxygen concentration, such that as the oxygen concentration approaches zero, the proliferation rates double compared to those observed under normal oxygen levels (Conley, Gheordunescu et al. 2012). The transition rates of cancer cells from type *i* to type *j* are denoted by p*_ij_* (Goldman, Majumder et al. 2015).  Moreover, the parameter *λ_M1_* signifies the tumoricidal activity of M1-like TAMs against cancer cells (Mahlbacher, Curtis et al. 2018).

The relationship between tumor cells proliferation and the local oxygen concentration, *G*, is asserted to follow Michaelis-Menten kinetics, taking the form presented in Equation (S8) (Casciari, Sotirchos et al. 1992, Casciari, Sotirchos et al. 1992):

| $G=\frac{k_{1} c_{ox}}{k_{2}+c_{ox}}$ | (S8) |
| --- | --- |

where *k_1_* and *k_2_* are parameters indicative of the growth rate, and $c_{ox}$ represents the concentration of oxygen.

The mechanism underlying the creation and degradation of the solid phases, *S_CCs_*, *S_SCCs_* and *S_ICCs_* is delineated as follows Equations (S9-S11):

| $S_{CCs}=GS_{f}T_{CCs}-cNT_{CCs}-D+p_{CT}T_{SCCs}+p_{IT}T_{ICCs}-\left(p_{TC}+p_{TI}\right)T_{CCs}-\lambda_{M1}M_{1}T_{CCs}$ | (S9) |
| --- | --- |
| $S_{SCCs}=\alpha_{SCCs}GS_{f_{SCCs}}T_{SCCs}-c_{SCCs}NC_{SCCs}-D_{SCCs}+p_{TC}T_{CCs}+p_{IC}T_{ICCs}-\left(p_{CT}+p_{CI}\right)T_{SCCs}$ | (S10) |
| $S_{ICCs}=\alpha_{ICCs}GS_{f_{ICCs}}T_{ICCs}-c_{ICCs}NT_{ICCs}-D_{ICCs}+p_{TI}T_{CCs}+p_{CI}T_{SCCs}-\left(p_{IT}+p_{IC}\right)T_{ICCs}$ | (S11) |

To evaluate the effect of drug delivery on tumor growth, the surviving cell fraction, denoted as *S_f_*, is incorporated into the cancer cell equation (Eq. (S5)). In the absence of pharmacological treatment, *S_f_* =1. The relationship between the surviving cell fraction and the internalized drug concentration (*c_int_*) has been experimentally determined for anti-cancer agents, and can be represented by an exponential model (Kerr, Kerr et al. 1986, Eikenberry 2009):

| $S_{urf}=\exp(-\omega c_{int})$ | (S12) |
| --- | --- |

According to this formulation, if immune cell effects are neglected and anti-cancer therapy eliminates *CCs*, *SCCs*, and *ICCs*, the terms *S_CCs_*, *S_CCSs_* and *S_ICCs_*  in Eq. (S9), (S10) and (S11) respectively, would converge to zero, causing the growth stretch ratio in Eq. (S4) to become constant. However, since the growth stretch ratio is expected to decrease following the cessation of *CCs*, *SCCs*, and *ICCs* proliferation, Eq. (15) was accordingly modified as follows:

| $S_{f}=2\cdot(\exp(\omega c_{int})-0.5)$ | (S13) |
| --- | --- |

Given that stem-like cancer cells (*SCCs*) and treatment-induced cancer cells (*ICCS*) exhibit chemoresistance, the equation is calibrated to align with empirical observations (Liu, Yuan et al. 2006) in order to determine the fitting parameter *ω* (*ω_SCCs_* and *ω*_ICCs_) pertinent to this context (*S_fSCCs_* and *S_fICCs_*).

**Immune cells**

For the immune system, this model considers four principal categories of immune cells: natural killer (NK) cells, CD8^+^ T-cells, CD4^+^ T-cells and regulatory T-cell (Treg) subset. Utilizing relevant research work (de Pillis, Radunskaya et al. 2005, Fouchet and Regoes 2008, Burroughs, Oliveira et al. 2011), the system of equations incorporates the recruitment rates of these immune cells, their inactivation by tumor cells, the suppressive function of Tregs and M2 tumor-associated macrophages (TAMs), as well as their mortality rate and interactions with cancer cells. The following Equations (S12-S15) illustrate the interactions that taking place among the cellular elements of the immune system:

| $\frac{\partial N}{\partial t}=\sigma_{nk}-f_{NK}N+\frac{g_{NK}T_{CCs}^{2}}{h+T_{CCs}^{2}}N-p_{im}NT_{CCs}-\lambda_{reg}T_{reg}N-\lambda_{M2}M_{2}N$ | (S14) |
| --- | --- |
| $\frac{\partial L}{\partial t}=\sigma_{T8}-m_{T8}L+\frac{j_{T8}D^{2}}{k_{im}+D^{2}}L-qLT_{CCs}+(r_{N}N+r_{Cd4}C_{d4})T_{CCs}-\lambda_{reg}T_{reg}L-\lambda_{M2}M_{2}L$ | (S15) |
| $\frac{\partial C_{d4}}{\partial t}=s_{CD4}+re_{Cd4}C_{d4}\left(1-\frac{C_{d4}}{C_{d4,max}}\right)-\mu_{C_{d4}}C_{d4}$ | (S16) |
| $\frac{\partial T_{reg}}{\partial t}=g_{reg}T_{reg}-m_{reg}T_{reg}$ | (S17) |

where *N* is the population of NK cells, $L$ of CD8^+^ T-cells, *C_d4_* denotes the population of CD4^+^ T-cells and *T_reg_* represents the population of regulatory T-cells. Additionally, $f_{NK}$, $m_{T8}$, and $m_{reg}$ signify the mortality rates of NK cells, CD8^+^ T-cells, and Treg cells, respectively, while $g_{NK}$, $j_{T8}$, and $g_{reg}$ denote the recruitment rates of immune cells, and $p_{im}$ and *q* represent the inactivation rates of immune cells by stem-like cancer cells (CCs). The constants $\sigma_{nk}$ and $\sigma_{T8}$ denote the stable sources of NK and CD8^+^ T-cells, respectively, $r_{N}$ indicates the rate at which tumor-specific CD8^+^ T-cells are stimulated for production due to tumor cells being eliminated by NK cells, and $\lambda_{reg}$ serves as the inhibition term affecting NK cells and CD8^+^ T-cells as influenced by Treg cells. Under conditions of hypoxia, the minimum activity levels for NK and CD8^+^ T-cells, were employed, which subsequently increased linearly to the maximum observed levels under normoxic conditions (de Pillis, Radunskaya et al. 2005). The values of $f_{NK}$ and $m_{T8}$ were adjusted to reflect variations in oxygen levels. In accordance with experimental data (Barsoum, Smallwood et al. 2014), a 40-fold reduction in oxygen concentration (from 20% to 0.5%) resulted in a twofold increase in the apoptotic rate of immune cells. Furthermore, $s_{CD4}$ serves as the source of CD4^+^ T-cells, $\mu_{C_{d4}}$ represents the natural mortality rate of CD4^+^ T-cells, ${re}_{Cd4}$ indicates the growth rate of CD4^+^ T-cells, and $C_{d4,max}$ signifies the maximum population of CD4^+^ T-cells (Perelson, Kirschner et al. 1993, Culshaw and Ruan 2000). The stimulation rate of CD8^+^ T-cells by CD4^+^ T-cells, as previously noted (de Pillis 2013, De Palma and Jain 2017, Tian, Goldstein et al. 2017), is represented by $r_{Cd4}$. The source term for CD4^+^ T-cells, $s_{CD4}$, is reliant on the concentration of oxygen, as previous studies have indicated an eightfold decrease under hypoxic conditions (Wang, Liu et al. 2010). Moreover, a reduction in M2 TAMs led to an increase in the populations of CD8^+^ T-cells and NK cells, while CD4^+^ T-cells remained unaffected, according to experimental findings (Rolny, Mazzone et al. 2011), and these dynamics are articulated by the parameter $\lambda_{M2}$. The equations that describe the cell populations are normalized by transforming them dimensionless through the division of the cell count per finite element node by the initial quantity of cancer cells, T0=5×10² cells. The initial cancer cell population was established as follows: 98% CCs, 1% SCCs, and 1% ICCs (Hermann, Huber et al. 2007).

Additionally, the variable *D* represents the fractional cell kill of tumor cells by CD8^+^ T-cells and is defined by Equation (S16) (de Pillis, Radunskaya et al. 2005, Milberg, Gong et al. 2019):

| $D=d_{im}\frac{\left(\frac{L}{T_{CCs}}\right)^{\lambda_{im}}}{s+\left(\frac{L}{T_{CCs}}\right)^{\lambda_{im}}}T_{CCs}$, | (S18) |
| --- | --- |

In this expression, $d_{im}$ signifies the saturation threshold of fractional tumor cell eradication by CD8^+^ T-cells, *s* reflects the steepness coefficient associated with the competition between the tumor and CD8^+^ T-cells, and $\lambda_{im}$ denotes the exponent corresponding to fractional tumor cell kill by CD8^+^ T-cells.

**Tumor associated macrophages (TAMs)**

In this mathematical model, two distinct classifications of TAMs are recognized, specifically *M_1_* and *M_2_*:

|  | (S19) |
| --- | --- |

The parameters $g_{m1}$ and $g_{m2}$ signify the production rates for *M_1_* and *M_2_* TAMs, which are influenced by oxygen concentrations, as indicated by prior investigations (Huang, Snuderl et al. 2011, Rolny, Mazzone et al. 2011, Huang, Stylianopoulos et al. 2013) showing that a decline in hypoxia leads to a reorientation in TAM polarization from the *M_2_*-like to the *M_1_*-like phenotype. Previous investigations have established a correlation between TAMs and the expression of VEGF (Stockmann, Doedens et al. 2008, Rolny, Mazzone et al. 2011, Linde, Lederle et al. 2012). In particular, the overexpression of VEGF-A has been linked to an increased presence of *M_2_*-like TAMs $(r_{Cvegf,M2})$.

**Implementation of biphasic theory for understanding the mechanical behavior of the tumor**

The conservation principles of both the solid and fluid phases within the tumor system are expressed through the subsequent mass balance equations: (Roose, Netti et al. 2003, Voutouri and Stylianopoulos 2014)

| $\frac{\partial\Phi^{c}}{\partial t}+\nabla\cdot(v^{s}\Phi^{c})=\frac{T_{CCs}}{T_{tot}}S_{CCs}+\frac{T_{SCCs}}{T_{tot}}S_{SCCs}+\frac{T_{ICCs}}{T_{tot}}S_{ICCs}$ | (S20) |
| --- | --- |
| $\frac{\partial\Phi^{f}}{\partial t}+\nabla\cdot(v^{f}\Phi^{f})=Q$ | (S21) |

In this context, *Φ^c^* and *Φ^f^* represent the volume fractions of the solid and fluid phases, respectively, while ***v****^s^*and ***v****^f^* denote their associated velocities.

The variable *Q* in Equation (S20) signifies the fluid flux entering the tumor from blood vessels as well as from the adjacent normal tissue, subtracted by the fluid flux exiting via lymphatic vessels, and is expressed as (Stylianopoulos, Martin et al. 2013):

| $Q=L_{p}S_{v}(p_{v}-p_{i})-L_{pl}S_{vl}(p_{i}-p_{l})$ | (S22) |
| --- | --- |

where *L_p_*, *S_v_*, and *p_v_* are indicative of the hydraulic conductivity, vascular density, and vascular pressure, respectively; *L_pl_*, *S_vl_*, and *p_l_* are the analogous parameters for lymphatic vessels; and *p_i_* corresponds to the interstitial fluid pressure. In this particular model configuration and taking into account the principle of mass conservation within the tissue, the total of the solid and fluid volume fractions is established to consistently amount to one, in accordance with Equation (S21).

| $\Phi^{f}=1-\Phi^{c}$ | (S23) |
| --- | --- |

Moreover, summing Equations (S18) and (S19), the mass balance can be expressed as follows:

| $\nabla\cdot\left(\Phi^{c}v^{s}+\Phi^{f}v^{f}\right)=Q+\frac{T_{CCs}}{T_{tot}}S_{CCs}+\frac{T_{SCCs}}{T_{tot}}S_{SCCs}+\frac{T_{ICCs}}{T_{tot}}S_{ICCs}$ | (S24) |
| --- | --- |

where the fluid velocity $v^{f}$ is defined according to Darcy’s law (Byrne and Preziosi 2003)

|  | (S25) |
| --- | --- |

with *k_th_* denoting the hydraulic conductivity of the interstitial medium (Stylianopoulos, Yeckel et al. 2008).

In accordance with the biphasic theory applicable to soft tissues (Mow, Kuei et al. 1980), the overall stress tensor ***σ****_tot_* is constituted by the fluid phase stress tensor $\sigma^{f}=-p_{i}I$ and the solid phase stress tensor **σ**^s^. Consequently, the stress balance can be expressed as:

| $\nabla\cdot\sigma_{tot}=0 \Rightarrow\nabla\cdot(\sigma^{s}-p_{i}I)=0$ | (S26) |
| --- | --- |

where the Cauchy stress tensor associated with the solid phase **σ**^s^ is delineated by: (Taber 2008)

| $\sigma^{s}=J_{e}^{-1}F_{e}\frac{\partial W}{\partial F_{e}^{T}}$, | (S27) |
| --- | --- |

The mechanical properties of the tumor have been defined as incompressible and neo-Hookean, with the strain energy density represented by: (Xu, Bayly et al. 2009, Xu, Kemp et al. 2010, Ciarletta 2013, Voutouri, Mpekris et al. 2014)

| $W=\frac{1}{2}\mu\left(-3+II_{1}\right)-p\left(J_{e}-I_{1}\right)-\left(\frac{p^{2}}{2k}\right)$ | (S28) |
| --- | --- |

where *μ* and $k$ denote the shear and bulk modulus of the material, respectively; $J_{e}$ signifies the determinant of the elastic deformation gradient tensor $F_{e}$; $II_{1}=I_{1}J_{e}^{-2/3}$ with $I_{1}=trC_{e}$being the first invariant of the elastic Cauchy-Green deformation tensor $C_{e}=F_{e}^{T}F_{e}$, and *p* is a penalty variable introduced for materials that exhibit near incompressibility that regularizes the constraint involved in the second term (Holzapfel, Gasser et al. 2000). Values of shear modulus are based on experimental findings from our previous studies (Mpekris, Panagi et al. 2024, Panagi, Mpekris et al. 2024). The adjacent normal tissue is presumed to be compressible and neo-Hookean, characterized by a Poisson ratio of 0.2 and tumor tissue with a Poisson ratio of 0.45.

**Functional vascular density**

To assess the functional vascular density, it is claimed that is impacted by the decrease in the diameter of blood vessels (d/d*_0_*) linked to the increase of solid stress (Mpekris, Angeli et al. 2015). Furthermore, the functional vascular density is dependent on the permeability of the tumor vascular wall (Stylianopoulos and Jain 2013), as hyper-permeable vessels diminish both perfusion and overall functionality of the vessels.

The functional vascular density can be expressed as:

| $S_{v}=\frac{d}{d_{0}}S_{v}^{0}\rho_{v}^{EC}$, | (S29) |
| --- | --- |

where $S_{v}^{0}$ will be determined by the dimensions of the pores in the vascular wall (i.e., its permeability) and $\rho_{v}^{EC}$ is related to the density of endothelial cells.

**Transport of oxygen**

The variation in oxygen levels within the tumor tissue is considered to be based on both its transport mechanisms, namely convection and diffusion, alongside the rate of oxygen utilized by the cells, as well as the influx of oxygen from the surrounding blood vessels (Roose, Netti et al. 2003, Kim, Stolarska et al. 2011), specifically described by the equation:

| $\frac{\partial c_{ox}}{\partial t}+\nabla\cdot\left(c_{ox}v^{f}\right)=D_{ox}\nabla^{2}c_{ox}-\frac{A_{ox}c_{ox}}{c_{ox}+k_{ox}}T_{tot}+P_{er}S_{V}\left(C_{iox}-c_{ox}\right)$, | (S30) |
| --- | --- |

where *c_ox_* represents the oxygen concentration, *D_ox_* denotes the diffusion coefficient for oxygen in the interstitial region, *A_ox_* and *k_ox_* are parameters  associated with oxygen uptake, *P_er_* indicates the vascular permeability of oxygen, which governs the diffusion across the walls of tumor vessels, and *C_iox_* is signifies the oxygen concentration present in the vascular system. The transport of oxygen across the vascular barrier is primarily governed by diffusion, as the contribution of convection is minimal in comparison to diffusion (Popel 1989). In view of the uniform reduction of interstitial fluid pressure observed in tumors, the pressure differentials both within the tissue and across the tumor vessel wall are minimal (Chauhan, Stylianopoulos et al. 2011), leading to an expectation of low Péclet numbers.

**Components of tumor vasculature**

In accordance with our mathematical model, we integrate the constituents of tumor vasculature, which include the endothelial cells, the vascular endothelial growth factor (VEGF), as well as Angiopoietin 1 and Angiopoietin 2.

T**ransport equation of endothelial cells**

The flux of endothelial cells is represented by the Equation (S29): (Schugart, Friedman et al. 2008)

|  | (S31) |
| --- | --- |

The proliferation of endothelial cells is influenced by the concentration of VEGF, as well as the density of endothelial cells. is the dimensionless density of endothelial cells. and $C_{vegf}^{0}$ are dimensionless and refer to VEGF concentrations. The diffusion coefficient of endothelial cells depends on Ang1 and Ang2, described by , where $\alpha$ and $b$are set to unity (Plank, Sleeman et al. 2004), *x_n_* denotes a chemotactic factor. The dimensionless concentration of endothelial cells is derived by normalizing with the reference concentration, . The constants $\lambda_{2}$ and $\lambda_{4}$ are positive parameters.

T**ransport equation of VEGF**

The concentration of Vascular Endothelial Growth Factor (VEGF) is influenced by various factors including diffusion, synthesis from cancer cells in hypoxic conditions, and interaction with endothelial cell receptors (Schugart, Friedman et al. 2008). The concentration of VEGF is represented by the following Equation (S30):

|  | (S32) |
| --- | --- |

where denotes the dimensionless VEGF concentration achieved by normalizing with a reference value and represents the dimensionless oxygen concentration normalized as: .

It is assumed that VEGF is exclusively synthesized by tumor cells, with its production being intensified in response to hypoxic conditions, as characterized by the oxygen tension parameter *G_α_* (Schugart, Friedman et al. 2008).

|  | (S33) |
| --- | --- |

VEGF becomes unavailable through its binding to receptors on endothelial cells, and it is also capable of diffusing within the tumor characterized by a diffusion coefficient $D_{VEGF}$. $\lambda_{10}$, $\lambda_{11}$ and $\lambda_{13}$ are defined as positive constants. Furthermore, the removal of CD4^+^ T-cells led to a notable upregulation of VEGF () without marked alterations in Ang1-Ang2 levels (Tian, Goldstein et al. 2017).

T**ransport equation of angiopoietin 1 (Ang1) and angiopoietin 2 (Ang2)**

The synthesis of the Angiopoietin 1 (Ang1) and Angiopoietin 2 (Ang2) is augmented under hypoxic conditions, which correlate with levels of vascular endothelial growth factor (VEGF) (Plank, Sleeman et al. 2004). Both angiopoietin 1 (Ang1, *α_1_*) and angiopoietin 2 (Ang2, *α_2_*) exhibit up-regulation in response to hypoxia and are secreted by endothelial cells.

| $\frac{\partial\widehat{\alpha}_{1}}{\partial t}=\frac{b_{1}}{\alpha_{1}^{0}}p_{c}+\mu_{1}(1-\widehat{\alpha}_{1})$ | (S34) |
| --- | --- |
| $\frac{\partial\hat{\alpha}_{2}}{\partial t}=\frac{b_{2}}{\alpha_{2}^{0}}G_{\alpha}(\hat{c}_{ox})\hat{e}e_{0}-\mu_{2}\hat{\alpha}_{2}$ | (S35) |

In this context, *b_1_*, *b_2_*, *μ_1_* and *μ_2_* are defined as positive constants. The dimensionless forms of Ang1 and Ang2 are derived through normalization with respect to a reference concentration, $\hat{\alpha}_{1}=\frac{\alpha_{1}}{\alpha_{1}^{0}}$ and . The term for oxygen tension, denoted as G*_α_*, corresponds to the parameters utilized for VEGF. To simplify the equations, we omit considerations of the diffusion processes associated with Ang1 and Ang2, as well as their interactions with specific Tie receptors (Gevertz and Torquato 2006, Billy, Ribba et al. 2009).

**Boundary Conditions**

**Host and tumor tissue boundary conditions**

To model spherical growth, we assume symmetry and simulate only one-eighth of the full tumor domain (**Fig. S2**). Symmetry is enforced by imposing zero normal displacement (**u**) on each of the three symmetry planes.

| $n\cdot u=0$ | (S36) |
| --- | --- |

where n is the outward unit normal.

Consistent with symmetry, zero normal flux boundary conditions are applied on the same planes for oxygen (*c_ox_*).

| $-n\cdot N_{i}=0\to-n\cdot\left(-D_{_{ox}}\nabla c_{ox}+v^{f}c_{ox}\right)=0$ | (S37) |
| --- | --- |

and for interstitial fluid pressure (no normal flow), based on Darcy’s law and the solid velocity **v***^s^*.

| $-n\cdot\left(-k_{th}\nabla p_{i}+v^{s}\right)$ | (S38) |
| --- | --- |

Similarly, no flux boundary conditions are imposed on the symmetry planes for TSLs (*c_l_*), free chemotherapy drug (*c_f_*,), bound drug (*c_b_*) and internalized drug (*c_int_*).

| $-n\cdot N_{i}=0\to-n\cdot\left(-D_{_{l}}\nabla c_{l}+v^{f}c_{l}\right)=0$ | (S39) |
| --- | --- |
| $-n\cdot N_{i}=0\to-n\cdot\left(-D_{f}\nabla c_{f}+v^{f}c_{f}\right)=0$ | (S40) |
| $-n\cdot N_{i}=0\to-n\cdot\left(-D_{_{b}}\nabla c_{b}+v^{f}c_{b}\right)=0$ | (S41) |
| $-n\cdot N_{i}=0\to-n\cdot\left(-D_{_{int}}\nabla c_{int}+v^{f}c_{int}\right)=0$ | (S42) |

For the acoustic field, a symmetry boundary condition is applied such that the normal component of acceleration (and thus velocity) is zero on the symmetry planes.

| $-n\cdot\left(-\frac{1}{\rho_{t0}}\nabla p_{1}\right)=0$ | (S43) |
| --- | --- |

For bioheat transfer, a zero heat flux boundary condition is applied on the symmetry planes, i.e., the normal heat flux is set to zero.

| $-n\cdot q=0\to-n\cdot\left(-k_{t}T_{t}\right)=0$ | (S44) |
| --- | --- |

**Host tissue boundary conditions**

On the external boundary of the host tissue domain, oxygen is prescribed to a constant physiological value representative of normal tissue.

| $c_{ox}=0.2 mol\cdot m^{3}$ | (S45) |
| --- | --- |

A Dirichlet boundary condition is imposed for interstitial fluid pressure on the outer boundaries of the host tissue, so that the pressure takes the value of the fluid pressure in normal tissues.

| $p_{i}=0 Pa$ | (S46) |
| --- | --- |

For drug transport, zero concentration is imposed at the outer boundaries for the liposomal, free, bound, and internalized drug species.

| $c_{0,c_{l}}=0 mol\cdot m^{3}$ | (S47) |
| --- | --- |
| $c_{0,c_{f}}=0 mol\cdot m^{3}$ | (S48) |
| $c_{0,c_{b}}=0 mol\cdot m^{3}$ | (S49) |
| $c_{0,c_{\mathrm{int}}}=0 mol\cdot m^{3}$ | (S50) |

For the acoustic pressure, we apply an impedance boundary condition at the outer boundary to capture the effective surface impedance of the skin and subcutaneous tissue. This approach incorporates the boundary response through an equivalent impedance, avoiding explicit geometric modeling of these layers.

| $-n\cdot\left(-\frac{1}{\rho_{t0}}\nabla p_{1}\right)=\frac{i\omega p_{1}}{Z_{i}}$ | (S51) |
| --- | --- |

where *Z_i_* is the boundary impedance, *ρ_t0_* is the tissue density, and *ω* is the angular frequency.

The impedance *Z_i_*, is modeled using simple serial *RLC (Resistor- Inductor -Capacitor)* circuit applied on a transducer area of *A_t_*=1.5 cm^2^,

| $Z_{i}=\frac{R_{s}}{A_{t}}+\frac{i\omega L_{s}}{A_{t}}+\frac{1}{i\omega C_{s}A_{t}}$ | (S52) |
| --- | --- |

with experimentally determined parameters *R_s_*=9.0 N∙s∙m^-1^, *L_s_*=0.53∙10^-3^ N∙s^2^∙m^-1^ and *C_s_*=5.3∙10^-6^ m∙N^-1^, which reflect the mechanical response of the skin and subcutaneous soft tissue (Håkansson, Carlsson et al. 1986).

Finally, a constant temperature boundary condition is imposed at the outer host tissue boundaries.

| $T_{0}=310.15 K$ | (S53) |
| --- | --- |

**Tumor-host tissue interface conditions**

At the tumor-host interface, continuity conditions are imposed. This implies continuity of the cell mass flux across the interface: the normal flux leaving one side must equal the normal flux entering the other side. Accordingly, for the tumor (***t***) and host (***h***) tissue, we impose continuity of the normal mass flux for:

(i) **tumor cell populations, including** non-stem-like cancer cells (*CCs*), stem-like cancer cells (*SCCs*) and treatment-induced cancer cells (*ICCs*). For each tumor cell type ***i***, we apply:

| $(-n)\cdot\left(-D_{i}\nabla_{i}+v^{s}c_{i}\right)_{t}=n\cdot\left(-D_{i}\nabla_{i}+v^{s}c_{i}\right)_{h}$ | (S54) |
| --- | --- |

(ii) i**mmune cell populations, including** natural killer (*NK*) cells, *CD8⁺ T-cells*, *CD4⁺ T-cells*, regulatory T-cells (*Tregs*), and tumor-associated macrophages (*TAMs*) ***M1-like TAMs*** and ***M2-like TAMs*.** For each immune cell type ***j***, we apply:

| $(-n)\cdot\left(-D_{j}\nabla_{j}+v^{s}c_{j}\right)_{t}=n\cdot\left(-D_{j}\nabla_{j}+v^{s}c_{j}\right)_{h}$ | (S55) |
| --- | --- |

(iii) **vascular components**, including endothelial cells (*ECs*), angiopoietins (*Ang*) and vascular endothelial growth factor (*VEGF*). For each vascular component ***k***, we apply:

| $(-n)\cdot\left(-D_{k}\nabla_{k}+v^{s}c_{k}\right)_{t}=n\cdot\left(-D_{k}\nabla_{k}+v^{s}c_{k}\right)_{h}$ | (S56) |
| --- | --- |

Here, **n** denotes the outward unit normal vector on the interface (with opposite directions on the two sides).

Mesh and time step independence assessment

To evaluate the sensitivity of the numerical predictions to spatial and temporal discretization, we performed a **mesh refinement** study and a **time-step refinement** study. Key outcome measures were compared at the **last computational day (Day 33)**. The **baseline mesh** consisted of **6,015** free tetrahedral finite elements and **51,628 degrees of freedom (DOF)**, whereas the **refined mesh** consisted of **17,955** elements and **139,361 DOF**. The quantities of interest were the **tumor volume** (mm³) and the **intratumoral drug concentration**, *c_int_*​ (mol/mm³). For the time-step analysis, simulations were repeated using identical model settings while reducing the time step size from *t* = 0.25 d to *t* ​= 0.125 d. For each metric *M*, the relative difference (percent change) between a test setting and the baseline setting was computed as:

| $\%\Delta=100\times\frac{\left\vert M_{test}-M_{baseline}\right\vert}{\left\vert M_{test}\right\vert}$ | (S57) |
| --- | --- |

where *M_test_* denotes the value obtained with the refined mesh or the reduced *t_max_*. The results are summarized in **Table S5**. Under mesh refinement (DOF: 51,628 → 139,361), the Day 33 tumor volume and intratumoral drug concentration *c_int_* changed by 0.24% and 0.11%, respectively. Under time step refinement (*t* = 0.25 d → *t* = 0.125 d), the corresponding Day 33 changes were 0.19% for tumor volume and 0.04 % for *c_int_*, confirming negligible sensitivity to temporal discretization within the tested range (**Table S5**). Overall, these small percent differences indicate that the reported predictions are effectively independent of both mesh and time-step refinements within the tested ranges.

**Time step refinement study for the sonication treatment window**

To evaluate the sensitivity of the predicted short-time thermal transient and temperature-triggered drug release to time discretization during treatment, we performed a time-step refinement study focused on the sonication/treatment window. Simulations were repeated using identical model settings while reducing the time step size from *t* **=** 9 s to *t* =4 s during the ultrasound-induced hyperthermia interval, which in the examined cases lasted 30 min. The quantities of interest were the peak temperature during sonication, *T_peak_* (°C), and the released drug, *c_f_* mol/m^3^ at the end of sonication. For each metric *M*, the relative difference (percent change) between the refined and baseline temporal settings was computed using **Eq. (S57)**. The results are summarized in **Table S6**. Under time-step refinement (*t* = 9 s → 4 s), the percent differences in *T_peak_*​ and *c_f_* were 0.12% and 0.02%, respectively, indicating negligible sensitivity to temporal discretization within the tested range (**Table S6**). Overall, these small percent differences support that the reported treatment window predictions are effectively independent of the tested time-step refinement.

Transport regime characterization using the Péclet number

In solid tumors the interstitial fluid pressure (IFP) is typically elevated and relatively uniform in the center, which reduces pressure gradients and leads to negligible interstitial velocities (Moradi Kashkooli, Bhandari et al. 2025). Consequently, transport in the tumor interior is expected to be diffusion dominated. Specifically, to characterize whether interstitial transport is dominated by convection or diffusion, we evaluated the Péclet number using the standard advection-diffusion ratio:

| $Pe=\frac{\left\vert u\right\vert L}{D_{j}}$ | (S58) |
| --- | --- |

where |**u**| is the magnitude of the interstitial fluid velocity, *L* is a small characteristic length scale close to the tumor center (here L=10^−4^ m) and *D_j_* is the effective diffusion coefficient in the interstitium ( *D_l_* for TSLs and *D_f_* for free drug). Using the interstitial velocity field from the flow solution and evaluating at therapy onset, we found ***Pe*** to be well below unity for both species in the tumor center. In particular, for TSLs we obtained ***Pe*** ≈ 0.2, indicating diffusion-dominated transport for the nanocarriers under these conditions. For the free drug, **Pe** ≈ 1.5×10^-3^, further confirming that diffusion is the dominant transport mechanism in the tumor interior.

2 Supplementary figures and tables

**2.1 Supplementary figures**

**
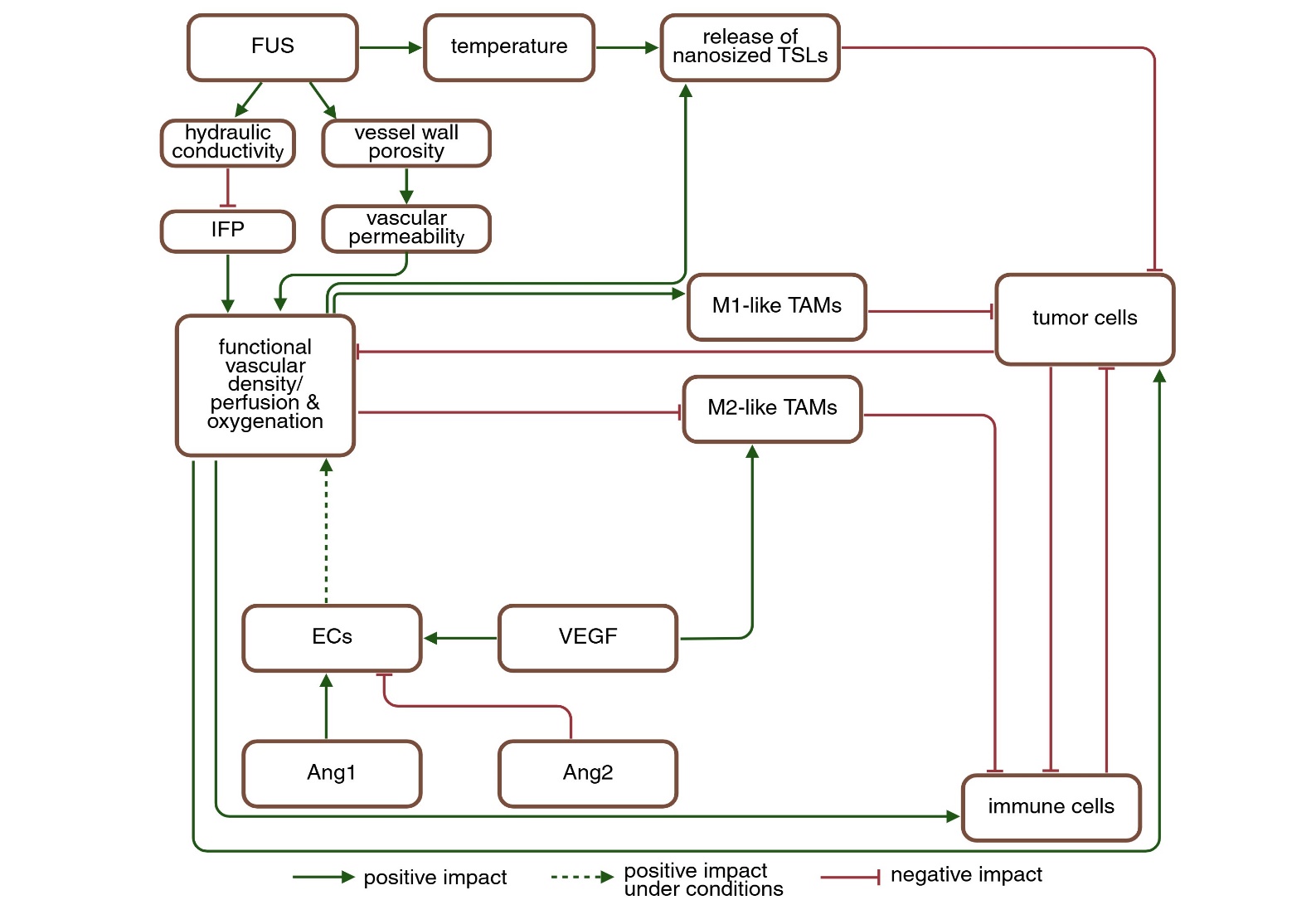
**

**Fig. S1**. Schematic representation of the mathematical model components and their interconnections. The model includes distinct cell populations and tumor angiogenic factors, each influencing the tumor microenvironment in specific ways. The diagram illustrates how individual components, and their combinations interact to affect functional vascular density, perfusion, and oxygenation within the system. These interactions can produce positive, negative, or condition-dependent effects.

**
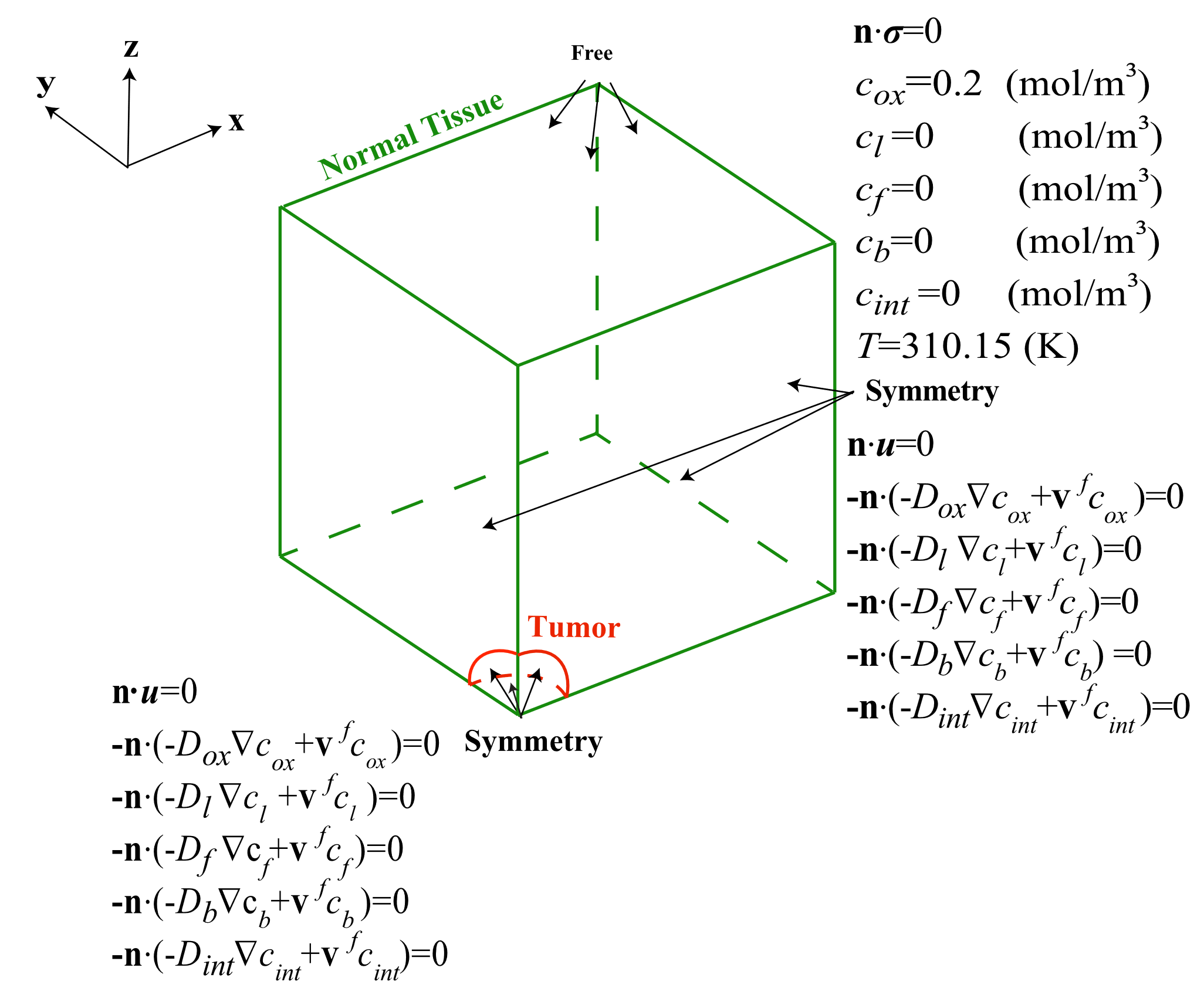
**

**Fig. S2.** The computational domain along with the specified boundary conditions that have been utilized for the current analysis of stress (***σ***), displacement (***u***), the concentration of oxygen (*c_ox_*), and the concentrations associated with the nanotherapy using thermosensitive liposomes, specifically denoted as $c_{l}$, $c_{f}$, $c_{b}$ and $c_{int}$.

**
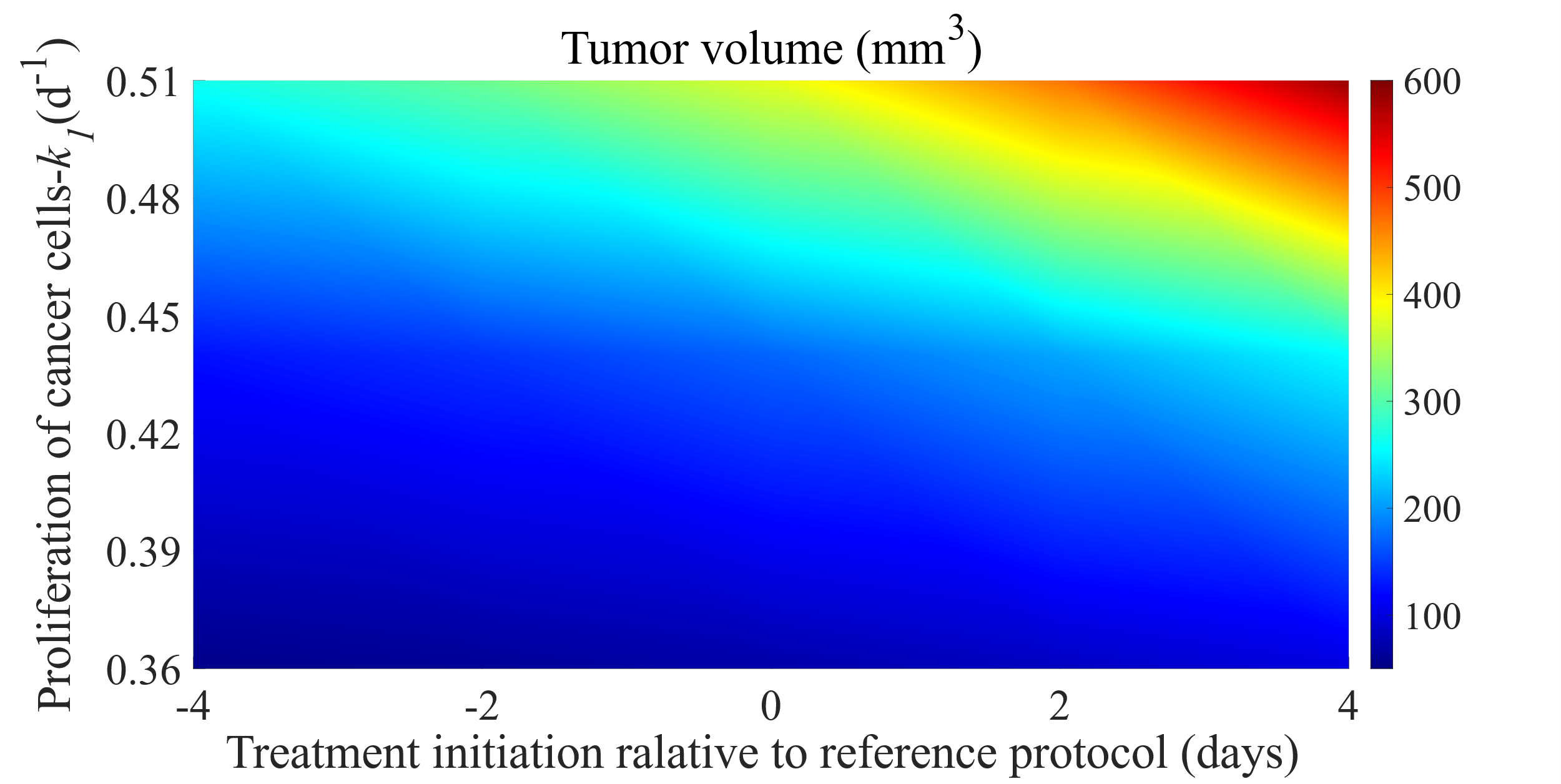
**

**Fig. S3.** Phase diagram depicting the influence of the cancer cell proliferation rate constant and the timing of combined therapy (FUS+TSLs) administration on the simulated tumor volume.

**
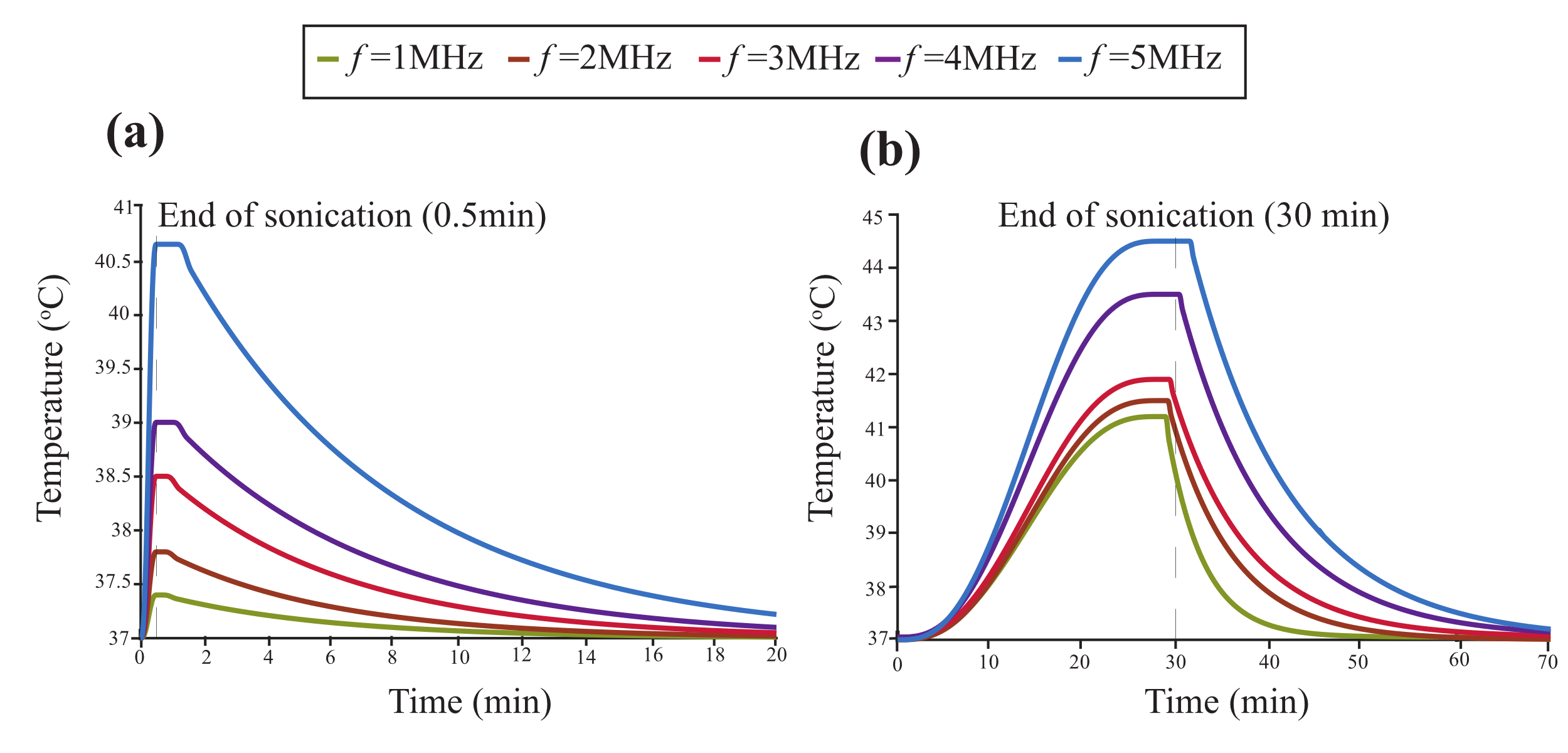
**

**Fig. S4.** Time-temperature curves at the tumor center during FUS sonication and subsequent cooling for ultrasound frequencies *f*=1-5 MHz. **(a)** *t*_exp_=0.5 min: the dashed vertical line denotes the end of sonication; temperature rises briefly and then decreases toward baseline during cooling. **(b)** *t*_exp_​ =30 min: prolonged sonication produces higher peak temperatures and a slower cooling-down phase after ultrasound termination (dashed vertical line).

**
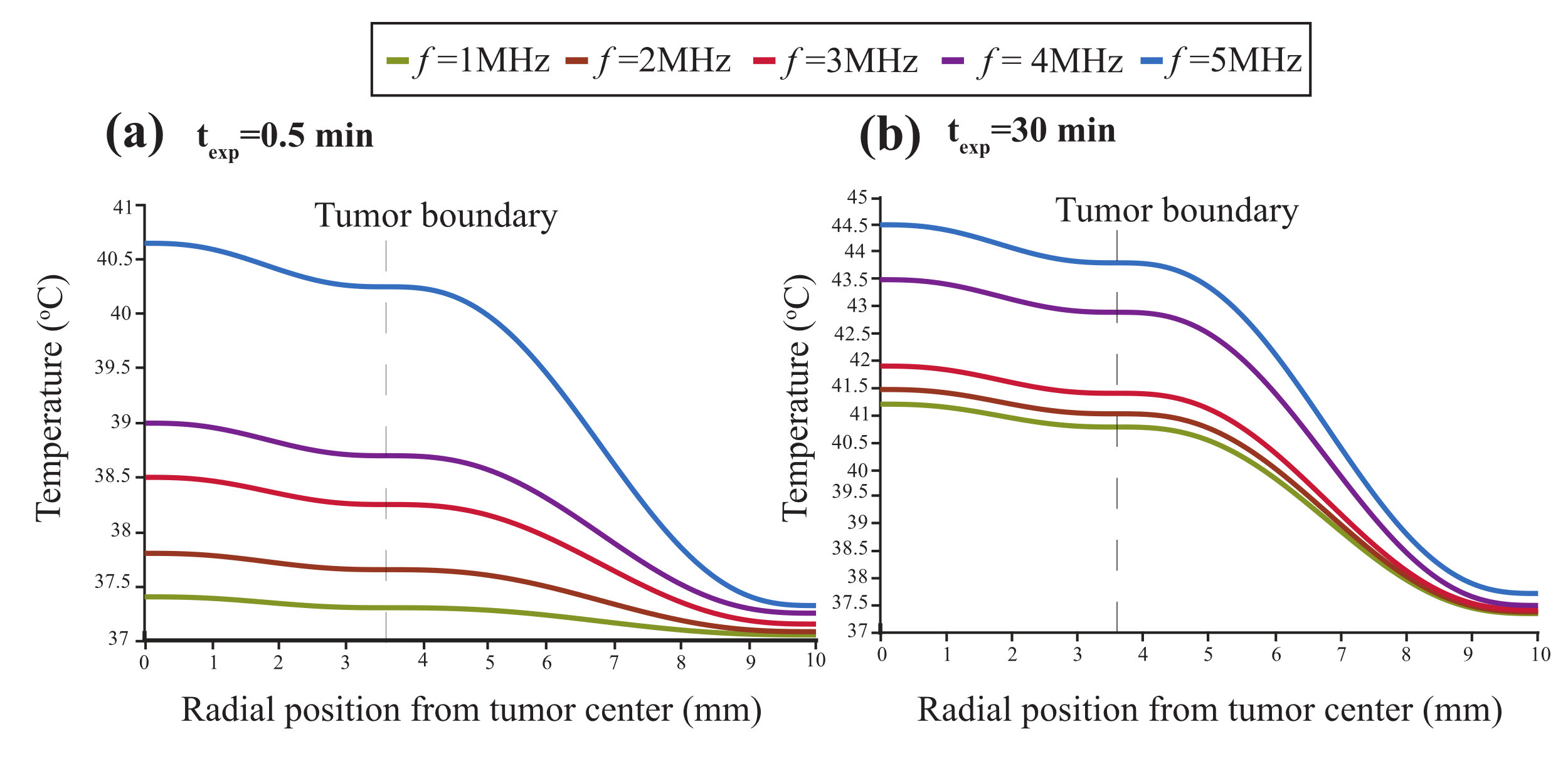
**

**Fig. S5.** Radial temperature profiles at the end of sonication for ultrasound frequencies *f*=1-5 MHz, plotted as a function of radial position from the tumor center. **(a)** *t*_exp_ =0.5 min. **(b)** *t*_exp_ = 30 min. The vertical dashed line indicates the tumor boundary (tumor-host tissue interface); temperatures decrease from the tumor interior into the surrounding host tissue.


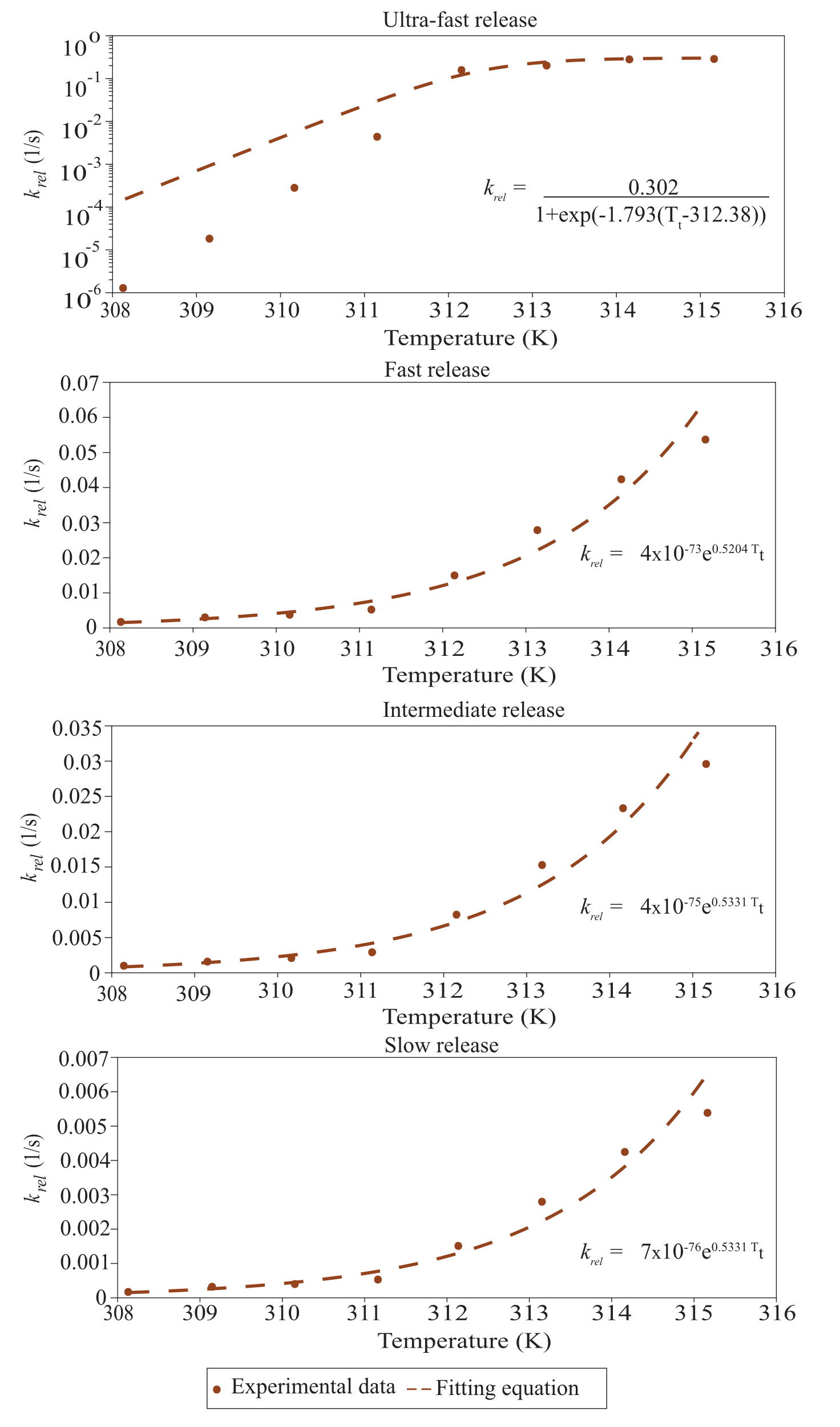


**Fig. S6.** Experimental release rate constants *k_rel_* (1/s) for the TSLs at different temperatures, along with their corresponding fitted curves and fitting equations for the **ultra-fast**, **fast**, **intermediate**, and **slow** release formulations. The *k_rel​_* values used for fitting are summarized in **Table S8**.


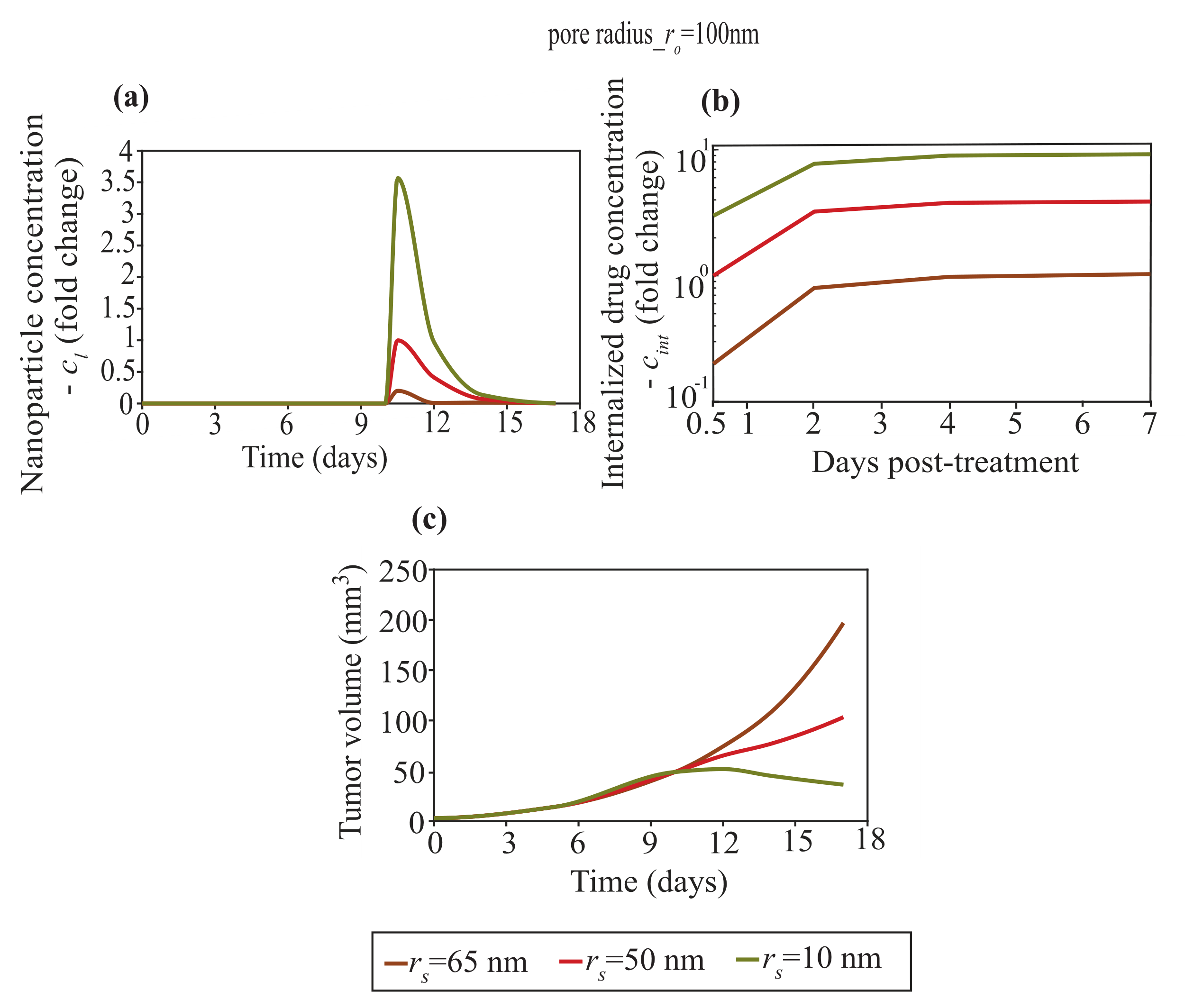


**Fig. S7.** Simulation results for three different nanoparticle sizes with fixed drug payload and for moderate vascular permeability: vessel wall pore radius *r_0_*​=100 nm, drug payload per carrier *α*=10^4^. Three particle radii were simulated (*r_s_*=10, 50, and 65 nm), while size-dependent diffusion coefficients *D_l_* were retained as in **Table S10**. **(a)** Temporal evolution of nanoparticle concentration *c_l​_*, **(b)** internalized drug concentration *c_int_* and **(c)** tumor volume. Nanoparticle concentrations are reported as fold change relative to the *r_s_*​=50 nm case at day 10 (day 0 post-treatment) and internalized drug concentrations are reported as fold change relative to the *r_s_*​=50 nm case at day 10.5 (day 0.5 post-treatment).

**
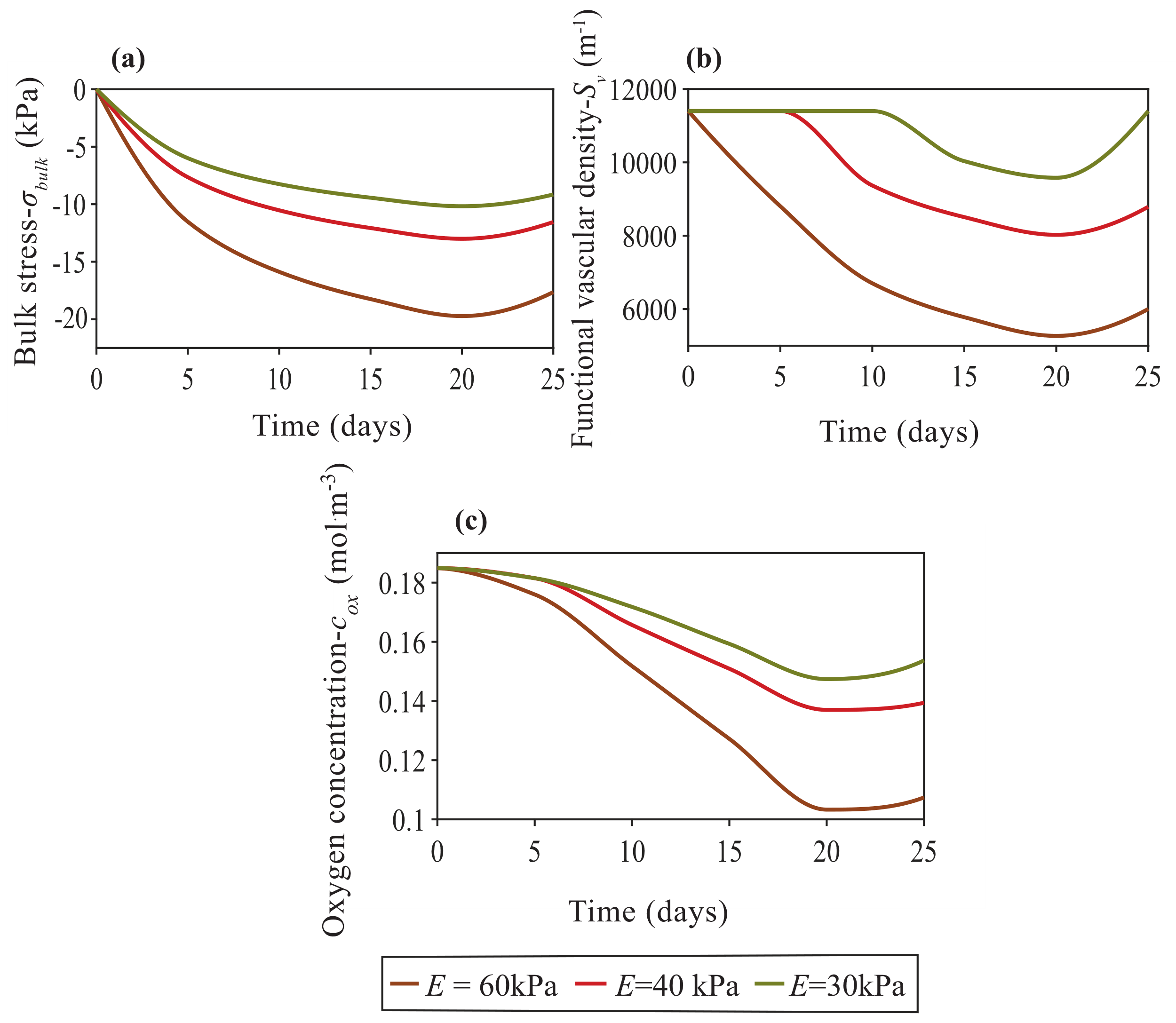
**

**Fig. S8.** Impact of different values of the tumor tissue elastic modulus (*E*) on (a) bulk stress (kPa), (b) functional vascular density (m^-1^) and (c) oxygen concentration (mol·m^-3^).


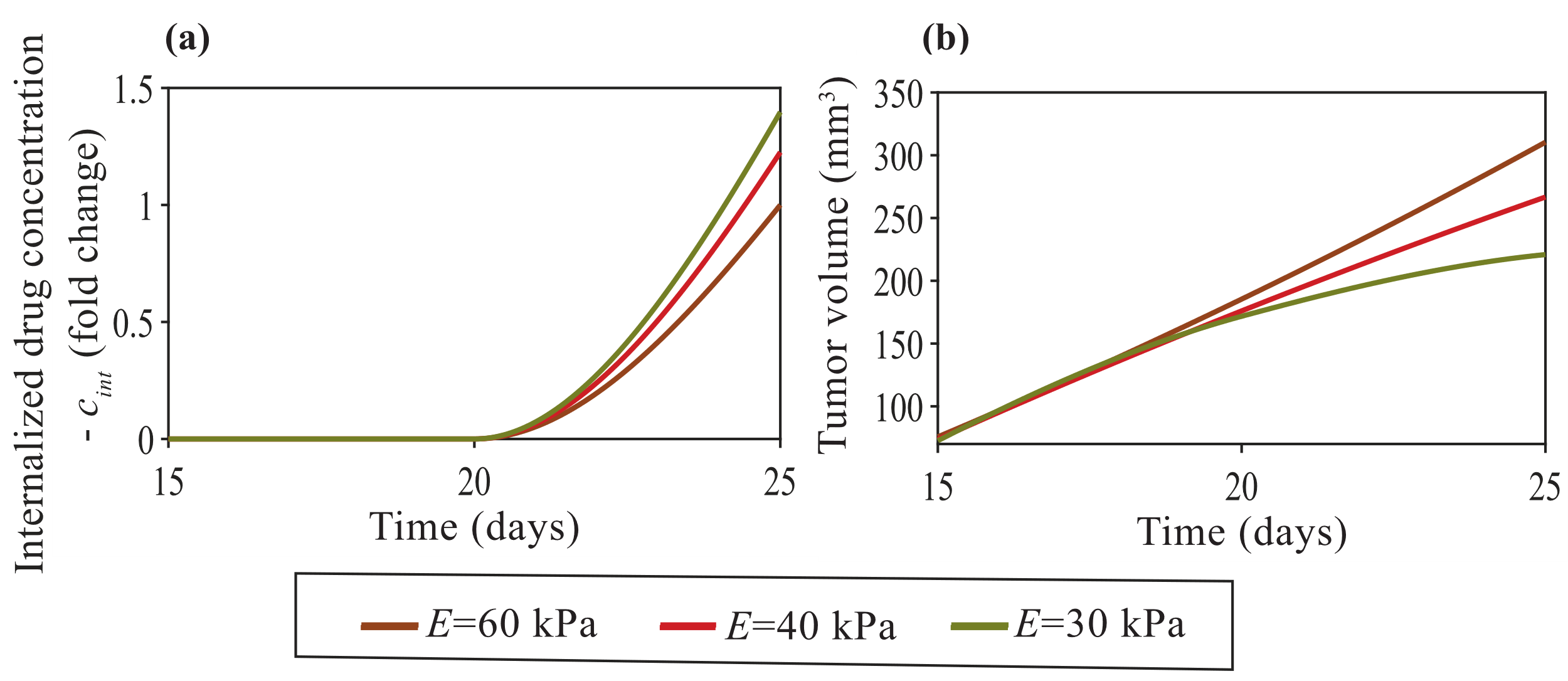


**Fig. S9.** Impact of different values of the tumor tissue elastic modulus (*E*) on (a) internalized drug concentration (fold change) and (b) tumor volume (mm^3^).

2.2 Supplementary tables

**Table S1.** Release rates of drug from temperature-sensitive liposomes (TSLs) at different temperatures (Tagami, May et al. 2012, Tehrani, Moradi Kashkooli et al. 2024). The release rate constants *k_rel_* (s^-1^) were interpolated using the exponential fitting equation: $k_{rel}=4\times10 ^{-73}e ^{0.5204T_{t}}$

| **Temperature (°C)** | 37 | 38 | 39 | 40 | 41 | 42 |
| --- | --- | --- | --- | --- | --- | --- |
| ***k*_rel_ (s^-1^)** | 0.0042 | 0.0054 | 0.015 | 0.028 | 0.042 | 0.054 |

**Table S2.** Parameter values utilized in this model.

| **Description** | **Parameter** | **Value [Units]** | **Reference(s)** |
| --- | --- | --- | --- |
| Drug diffusion coefficient in the interstitial fluid | *D*_f_ | 1.58 x 10^-10^ [m^2^/s] for normal tissue | (Wu, Klitzman et al. 1993, Goh, Kong et al. 2001, Tehrani, Moradi Kashkooli et al. 2024) |
|  |  | 3.4 x 10^-10^ [m^2^/s] for tumor tissue |  |
| TSL diffusion coefficient in the interstitial fluid | *D*_l_ | 6.31 x 10^-13^ [m^2^/s] for normal tissue | (Pluen, Boucher et al. 2001, Stylianopoulos, Economides et al. 2015, Tehrani, Moradi Kashkooli et al. 2024) |
|  |  | 4.51 x 10^-13^ [m^2^/s] for tumor tissue |  |
| Binding (association) rate constant | *k*_on_ | 1.5 x 10^3^ 1/(M⋅s) | (Moradi Kashkooli, Soltani et al. 2020, Tehrani, Moradi Kashkooli et al. 2024) |
| Unbinding (dissociation) rate constant | *k*_off_ | 8 x 10^-3^ [1/s] | (Moradi Kashkooli, Soltani et al. 2020, Tehrani, Moradi Kashkooli et al. 2024) |
| Internalization rate constant | *k*_int_ | 5 x 10^-5^ [1/s] | (Mok, Stylianopoulos et al. 2009, Schmidt and Wittrup 2009, Moradi Kashkooli, Soltani et al. 2020, Tehrani, Moradi Kashkooli et al. 2024) |
| Release rate of chemotherapeutic drug from temperature-sensitive liposomes (TSLs) | *k*_rel_ | 0.05 [1/s] | (Tagami, May et al. 2012, Tehrani, Moradi Kashkooli et al. 2024). |
| Volume fraction of tumor accessible to drugs | *φ* | 0.05 [-] | (Eikenberry 2009) |
| Concentration of cell-surface receptors | *c*_rec_ | 1 x 10^-5^ [M] | (Stylianopoulos, Economides et al. 2015, Tehrani, Moradi Kashkooli et al. 2024) |
| Half-life of TSL in plasma | *k*_d_ | 1440 [min] | (Chauhan, Stylianopoulos et al. 2012, Stylianopoulos, Economides et al. 2015, Tehrani, Moradi Kashkooli et al. 2024) |
| Chemotherapy molecules contained in  the thermosensitive liposomal nanocarrier | *α* | 10^4^ [-] | (Dawidczyk, Kim et al. 2014) |
| Radius of the diffusing nanoparticle | *r*_s_ | 50x10^-9^ [m] | (Pluen, Boucher et al. 2001, Dawidczyk, Kim et al. 2014) |
| Non-stem-like cancer cell survival constant | *ω* | 0.007 [m^3^/mol] | (Eikenberry 2009) |
| Stem-like cancer cell survival constant | *ω_SCCs_* | 0.0035 [m^3^/mol] | (Liu, Yuan et al. 2006, Eikenberry 2009) |
| Treatment-induced cancer cell survival constant | *ω_ICCs_* | 0.0035 [m^3^/mol] | ---- |
| Vessel wall thickness | *L*_vw_ | 5×10^−6^ [m] | (Stylianopoulos, Martin et al. 2013) |
| Water viscosity at 310K | *η* | 7×10^−4^ [Pa∙s] | (Stylianopoulos, Martin et al. 2013) |
| Absolute temperature | *T*_emp_ | 310.15 [K] | ---- |
| Fraction of vessel wall surface area occupied by pores | *γ* | 1×10^−4^ [-] | (Chauhan, Stylianopoulos et al. 2012) |
| Tissue density | *ρ*_t0_ | 1044 [kg/m^3^] | (Huang, Holt et al. 2004, Sheu, Solovchuk et al. 2011, Zhan, Gedroyc et al. 2019, Souri, Kiani Shahvandi et al. 2023, Tehrani, Moradi Kashkooli et al. 2024) |
| Specific heat | *c*_t0_ | 3710 [J/(kg∙K)] | (Huang, Holt et al. 2004, Sheu, Solovchuk et al. 2011, Zhan, Gedroyc et al. 2019, Namakshenas and Mojra 2023) |
| Thermal conductivity of tissue | *k*_t0_ | 0.59 [W/(m∙K)] | (Huang, Holt et al. 2004, Sheu, Solovchuk et al. 2011, Zhan, Gedroyc et al. 2019, Namakshenas and Mojra 2023, Tehrani, Moradi Kashkooli et al. 2024) |
| Local acoustic absorption coefficient of tissue | *α*_t_ | 8.55 [Np/m ∙MHz] | (Huang, Holt et al. 2004, Rezaeian, Sedaghatkish et al. 2019, Namakshenas and Mojra 2023) |
| Acoustic frequency | *f* | 1 [MHz] | (Moradi Kashkooli, Souri et al. 2023, Namakshenas and Mojra 2023) |
| Speed of ultrasound | *c*_0_ | 1568 [m/s] | (Huang, Holt et al. 2004, Sheu, Solovchuk et al. 2011, Zhan, Gedroyc et al. 2019, Souri, Kiani Shahvandi et al. 2023, Tehrani, Moradi Kashkooli et al. 2024) |
| The heat generated by metabolism | *Q*_m_ | 0 W/m^3^ | (Rezaeian, Sedaghatkish et al. 2019, Moradi Kashkooli, Souri et al. 2023) |
| The exposure duration of the focused ultrasound (FUS) | *t*_exp_ | 15 [min] | (Moradi Kashkooli, Jakhmola et al. 2023, Moradi Kashkooli, Hornsby et al. 2024, Moradi Kashkooli, Bhandari et al. 2025) |
| Coefficient for *K*_t_ | *α*_1_ | -73/60 [-] | (Deen 1987) |
| Coefficient for *K*_t_ | *α*_2_ | 77.293/50.400 [-] | (Deen 1987) |
| Coefficient for *K*_t_ | *α*_3_ | -22.5083 [-] | (Deen 1987) |
| Coefficient for *K*_t_ | *α*_4_ | -5.617 [-] | (Deen 1987) |
| Coefficient for *K*_t_ | *α*_5_ | -0.3363 [-] | (Deen 1987) |
| Coefficient for *K*_t_ | *α*_6_ | -1.216 [-] | (Deen 1987) |
| Coefficient for *K*_t_ | *α*_7_ | 1.647 [-] | (Deen 1987) |
| Coefficient for *K*_s_ | *b*_1_ | 7/60 [-] | (Deen 1987) |
| Coefficient for *K*_s_ | *b*_2_ | -2.227/50.400 [-] | (Deen 1987) |
| Coefficient for *K*_s_ | *b*_3_ | 4.0180 [-] | (Deen 1987) |
| Coefficient for *K*_s_ | *b*_4_ | -3.9788 [-] | (Deen 1987) |
| Coefficient for *K*_s_ | *b*_5_ | -1.9215 [-] | (Deen 1987) |
| Coefficient for *K*_s_ | *b*_6_ | 4.392 [-] | (Deen 1987) |
| Coefficient for *K*_s_ | *b*_7_ | 5.006 [-] | (Deen 1987) |

**Table S3.** Parameter values applied within the model.

| **Description** | **Parameter** | **Value [Units]** | **Reference(s)** |
| --- | --- | --- | --- |
| Hydraulic conductivity | *k*_th_ | 6.5×10^−10^ [m^2^∙Pa^−1^∙day^−1^] for the host tissue,  6.5×10^−10^ [m^2^∙Pa^−1^∙day^−1^] for the tumor | (Netti, Berk et al. 2000, Papageorgis, Polydorou et al. 2017) |
| Elastic or Young’s modulus | E | 21 [kPa] for the host tissue,  60 [kPa] for the tumor | Netti, Berk et al. 2000, Samani, Zubovits et al. 2007, Eder, Raith et al. 2014, Mpekris, Panagi et al. 2024, Panagi, Mpekris et al. 2024) |
| Shear modulus | *G,μ* | 8.75 [kPa] for the host tissue,  20.69 [kPa] for the tumor | (Netti, Berk et al. 2000, Samani, Zubovits et al. 2007, Eder, Raith et al. 2014, Mpekris, Panagi et al. 2024, Panagi, Mpekris et al. 2024) |
| Bulk modulus | *k* | 11.67 [kPa] for the host tissue,  200 [kPa] for the tumor | (Netti, Berk et al. 2000, Samani, Zubovits et al. 2007, Eder, Raith et al. 2014, Mpekris, Panagi et al. 2024, Panagi, Mpekris et al. 2024) |
| Fractional tumor cell killing by NK cells | *c* | range : 3.23×10^−7^ -3.23×10^−6^ [cell^−1^∙day^–1^] | (de Pillis, Radunskaya et al. 2005) |
| Cell diffusion coefficient | *D_cell_* | 1.5x10^-11^ [m^2^/s] | (Tracqui 1995, Voutouri, Kirkpatrick et al. 2019) |
| Stem-like-cancer cell growth multiplier | *α_SCCs_* | range : 1-2 [-] | (Burroughs, Oliveira et al. 2011) |
| Treatment-induced cancer cell growth multiplier | *α_ICCs_* | range : 1-2 [-] | **---** |
| Rate of transition from non-stem-like cancer cells to stem-like cancer cells | *p_TC_* | 0.55 [day^–1^] | (Goldman, Majumder et al. 2015) |
| Rate of transition from stem-like cancer cells to non-stem-like cancer cells | *p_CT_* | 1 [day^–1^] | (Goldman, Majumder et al. 2015) |
| Rate of transition from stem-like cancer cells to treatment-induced cancer cells | *p_CI_* | 0.58 [day^–1^] | (Goldman, Majumder et al. 2015) |
| Rate of transition from treatment-induced cancer cells to stem-like cancer cells | *p_IC_* | 0.96 [day^–1^] | (Goldman, Majumder et al. 2015) |
| Rate of transition from non-stem-like cancer cells to treatment- induced cancer cells | *p_TI_* | 0.21 [day^–1^] | (Goldman, Majumder et al. 2015) |
| Rate of transition from treatment-induced cancer cells to non-stem-like cancer cells | *p_IT_* | 1 [day^–1^] | (Goldman, Majumder et al. 2015) |
| Tumoricidal effect of M1 TAMs in cancer cells | *λ_M1_* | 3 [d^–1^] | (Mahlbacher, Curtis et al. 2018) |
| Growth rate parameter | *k_1_* | 0.61 [day^−1^] | **---** |
| Growth rate parameter | *k_2_* | 0.0083 [mol∙m^−3^] | (Casciari, Sotirchos et al. 1992) |
| Constant source of NK cells | *σ_nk_* | 1.3×10^4^ [cell∙day^–1^] | (de Pillis, Radunskaya et al. 2005) |
| Death rate of NK cells | *f_NK_* | range0.0412 - 0.0814 [day^–1^] | (de Pillis, Radunskaya et al. 2005) |
| Recruitment rate of NK cells | *g_NK_* | 0.025 [day^–1^] | (de Pillis, Radunskaya et al. 2005) |
| Steepness coefficient of NK cell recruitment curve | *h* | 2.02×10^7^ [cell^2^] | (de Pillis, Radunskaya et al. 2005) |
| Inactivation rate of NK cells | *p_im_* | 1×10^−7^ [cell^−1^∙day^–1^] | (de Pillis, Radunskaya et al. 2005) |
| Inhibition term of NK cells and CD8^+^ T-cells from Treg cells | *λ_reg_* | 100 [cell^−1^∙day^–1^] | (Fouchet and Regoes 2008) |
| Death rate of CD8^+^ T-cells | *m_T8_* | range : 0.02 - 0.04  [day^-1^] | (de Pillis, Radunskaya et al. 2005) |
| Death rate of regulatory T-cells | *m_reg_* | 0.02 [day^–1^] | (Fouchet and Regoes 2008) |
| Recruitment rate of CD8^+^ T-cells | *j_T8_* | 0.0375 [day^–1^] | (de Pillis, Radunskaya et al. 2005) |
| Recruitment rate of regulatory T-cells | *g_reg_* | 0.0375 [day^–1^] | (Fouchet and Regoes 2008) |
| Steepness coefficient of CD8^+^ T-cells recruitment curve | *k_im_* | 2.02×10^7^ [cell^2^] | (de Pillis, Radunskaya et al. 2005) |
| Inactivation rate of CD8^+^ T-cells | *q* | 3.42×10^−10^ [cell^−1^∙day^–1^] | (de Pillis, Radunskaya et al. 2005) |
| Stimulation rate of CD8^+^ T-cells | *r* | 1.1×10^−7^ [cell^−1^∙day^–1^] | (de Pillis, Radunskaya et al. 2005) |
| Source term of CD4^+^ T- cells | *s_CD4_* | 150 [day^–1^] | (Perelson, Kirschner et al. 1993) |
| Natural death rate of CD4^+^ T-cells | *μ_Cd4_* | 0.02 [day^–1^] | (Perelson, Kirschner et al. 1993) |
| Growth rate of CD4^+^ T-cells | *re_Cd4_* | 0.03 [day^–1^] | (Perelson, Kirschner et al. 1993) |
| Stimulation rate of CD8^+^ T cells by CD4^+^ T-cells | *r_Cd4_* | 1x10^-15^ [cells^-1^.day^-1^] | (de Pillis, Radunskaya et al. 2005) |
| Source term of CD8^+^ T- cells | *σ_T8_* | 150 [day^–1^] | **----** |
| Fractional tumor cell killing by CD8^+^ T-cells | *d_im_* | range: 1.43 – 7.15 [day^–1^] | (de Pillis, Radunskaya et al. 2005) |
| Exponent of fractional cell kill by CD8^+^ T-cells | *λ_im_* | 1.36 [-] | (de Pillis, Radunskaya et al. 2005) |
| Steepness coefficient of the tumor-CD8^+^ T-cells competition term | *s* | 2.73 [-] | (de Pillis, Radunskaya et al. 2005) |
| Death rate of regulatory M1 TAMs | *m_m1_* | 0.02 [day^–1^] | **----** |
| Death rate of regulatory M2 TAMs | *m_m2_* | 0.02 [day^–1^] | **----** |
| Initial oxygen concentration | *C_iox_* | 0.2 [mol∙m^−3^] | (Mpekris, Baish et al. 2017) |
| Oxygen diffusion coefficient | *D_ox_* | 1.55×10^−4^ [m^2^∙day^−1^] | (Kim, Stolarska et al. 2011) |
| Oxygen uptake | *A_ox_* | 2200 [mol∙m^−3^∙day^−1^] | (Casciari, Sotirchos et al. 1992, Kim, Stolarska et al. 2011) |
| Oxygen uptake | *k_ox_* | 0.00464 [mol∙m^−3^] | (Casciari, Sotirchos et al. 1992, Kim, Stolarska et al. 2011) |
| Endothelial cell diffusion coefficient | *D_EC_* | 1x10^-15^ [m^2^/s] | (Plank, Sleeman et al. 2004) |
| Chemotactic endothelial cell | *x_n_* | 2x10^-15^ [m^5^/kg-s] | (Schugart, Friedman et al. 2008) |
| Reference value of endothelial cell | *e_0_* | 1x10^-3^ [g/cm^3^] | (Schugart, Friedman et al. 2008) |
| Positive parameter | *λ_2_* | 1x10^-5^ [cm^3^/g-s] | (Schugart, Friedman et al. 2008) |
| Positive parameter | *λ_4_* | 1x10^-1^ [cm^3^/g-s] | (Schugart, Friedman et al. 2008) |
| Positive parameter | *s1* | 1x10^3^ [cm^3^/ g] | (Plank, Sleeman et al. 2004) |
| Positive parameter | *s2* | 1x10^3^ [cm^3^/g] | (Plank, Sleeman et al. 2004) |
| VEGF diffusion coefficient | *D_VEGF_* | 3.1x10^-11^ [m^2^/s] | (Schugart, Friedman et al. 2008) |
| Reference VEGF concentration | $C_{vegf}^{0}$ | 1x10^-3^ [g/cm^3^] | (Schugart, Friedman et al. 2008) |
| Positive parameter | *λ_10_* | 6.8x10^-3^ [1/s] | (Schugart, Friedman et al. 2008) |
| Positive parameter | *λ_11_* | 4 [cm^3^/g-s] | (Schugart, Friedman et al. 2008) |
| Positive parameter | *λ_13_* | 4x10^-5^ [1/s] | (Schugart, Friedman et al. 2008) |
| Reference α_1_ concentration | $\alpha_{1}^{0}$ | 1x10^-3^ [g/cm^3^] | (Plank, Sleeman et al. 2004) |
| Reference α_2_ concentration | $\alpha_{2}^{0}$ | 1x10^-3^ [g/cm^3^] | (Plank, Sleeman et al. 2004) |
| Positive parameter | *b_1_* | 2280 [1/h] | (Plank, Sleeman et al. 2004) |
| Positive parameter | *b_2_* | 18240 [1/h] | (Plank, Sleeman et al. 2004) |
| Positive parameter | *μ_1_* | 456 [1/h] | (Plank, Sleeman et al. 2004) |
| Positive parameter | *μ_2_* | 456 [1/h] | (Plank, Sleeman et al. 2004) |

: linear increase from minimum to maximum value depending on oxygen levels

: linear decrease from maximum to minimum value depending on oxygen levels

**Table S4.** The initial values of the variables used in the mathematical model at time t=0 day.

| **Description** | **Parameter** | **Initial Value [Units]** | **Reference(s)** |
| --- | --- | --- | --- |
| Tumor cell populations | | | |
| Non-stem-like cancer cells | *CCs* | 0.96 [-] | (Mpekris, Voutouri et al. 2020) |
| Stem-like-cancer cells | *SCCs* | 0.02 [-] | (Mpekris, Voutouri et al. 2020) |
| Treatment-induced-cancer cells | *ICCs* | 0.02 [-] | (Mpekris, Voutouri et al. 2020) |
| Immune cell populations | | | |
| Natural killer cells | *NK* | 0 [-] | (Mpekris, Voutouri et al. 2022) |
| CD8^+^ T-cells | *CD8T_cells* | 0 [-] | (Hadjigeorgiou and Stylianopoulos 2024) |
| CD4^+^ T-cells | *CD4T_cells* | 0 [-] | (Mpekris, Voutouri et al. 2022) |
| Regulatory T-cells | *Treg* | 0.01 [-] | (Mpekris, Voutouri et al. 2022) |
| Tumor associated macrophages (TAMs) | | | |
| TAMs M1 | *M1* | 0.01 [-] | (Mpekris, Voutouri et al. 2022) |
| TAMs M2 | *M2* | 0.01 [-] | (Mpekris, Voutouri et al. 2022) |
| Tumor vasculature components | | | |
| Angiopoietin 1 | *Ang1* | 0 [-] | (Mpekris, Voutouri et al. 2020) |
| Angiopoietin 2 | *Ang2* | 0 [-] | (Mpekris, Voutouri et al. 2020) |
| Endothelial cells | *ECs* | 0.5 [-] | (Mpekris, Voutouri et al. 2020) |
| Vascular endothelial growth factor | *VEGF* | 0 [-] | (Mpekris, Voutouri et al. 2020) |
| Thermosensitive liposomal nanotherapy | | | |
| TSL concentration | *c*_l_ | 0 [mol/m^3^] | (Mpekris, Baish et al. 2017) |
| Free chemotherapeutic agent concentration | *c*_f_ | 0 [mol/m^3^] | (Mpekris, Baish et al. 2017) |
| Bound drug concentration | *c*_b_ | 0 [mol/m^3^] | (Mpekris, Baish et al. 2017) |
| Internalized chemotherapeutic agent concentration | *c*_int_ | 0 [mol/m^3^] | (Mpekris, Baish et al. 2017) |
| Focused ultrasound propagation induced hyperthermia | | | |
| Acoustic pressure | *p*_1_ | 0 [Pa] | (Moradi Kashkooli, Souri et al. 2023) |
| Temperature | *T* | 310.15 [K] | (Rezaeian, Sedaghatkish et al. 2019) |
| Other variables | | | |
| Oxygen concentration | *c_ox_* | 0.2 [ mol/m^3^] | (Mpekris, Baish et al. 2017) |
| Interstitial fluid pressure | *p* | 0 [Pa] | (Hadjigeorgiou and Stylianopoulos 2024) |
| Growth stretch ratio | *λ_g_* | 1 [-] | (Hadjigeorgiou and Stylianopoulos 2024) |
| Solid mechanics, displacement vector | ***u*** | 0 [m] | (Hadjigeorgiou and Stylianopoulos 2024) |

**Table S5.** Mesh and time step independence assessment at the last computational day (Day 33). Baseline and refined discretizations are compared using tumor volume and intratumoral drug concentration *c_int_*​. Mesh refinement increases the resolution from 6,015 to 17,955 tetrahedral elements (DOF: 51,628 → 139,361), while time step refinement reduces the time step size from *t*=0.25 d to *t*=0.125 d. Percent changes are computed as %Δ=100 ∣*M_test_*−*M_baseline_*∣/∣*M_test_*∣.

| **Verification test** | **Case** | **Elements** | **DOF** | **Time step (days)** | **Tumor volume (mm^3^)** | | ***c_int_ (mol/mm^3^)*** | **% change (tumor volume)** | **% change (*c_int_*)** |
| --- | --- | --- | --- | --- | --- | --- | --- | --- | --- |
| Mesh refinement | Baseline | 6,015 | 51,628 | 0.25 | | 532.78 | 5.301•10^−8^ | **--** | **--** |
|  | Refined | 17,955 | 139,361 | 0.25 | | 534.06 | 5.295•10^−8^ | 0.24 % | 0.11 % |
| Time step refinement | Baseline | 6,015 | 51,628 | 0.25 | | 532.78 | 5.301•10^−8^ | **--** | **--** |
|  | Refined | 6,015 | 51,628 | 0.125 | | 533.78 | 5.299 •10^−8^ | 0.19 % | 0.04 % |

| **Verification test** | **Case (*t*)** | ***T_peak_*​ (°C )** | ***c_f_ (mol/mm^3^)*** | **% change (*T_peak_*)** | **% change (*c_f_*)** |
| --- | --- | --- | --- | --- | --- |
| Time step refinement | Baseline (9 s) | 41.2 | 5.8220 •10^−11^ | **--** | **--** |
|  | Reduced (4 s) | 41.25 | 5.8209 •10^−11^ | 0.12 % | 0.02 % |

**Table S6.** Time-step refinement study for the sonication treatment window (Day 21, 30-min sonication). Baseline and refined temporal discretizations are compared using the peak temperature during sonication, *T_peak_*​ (°C), and the released drug, *c_f_* mol/m^3^ at the end of sonication. Time-step refinement reduces the time step size from *t*=9 s to *t*=4 s while keeping all other model settings identical. Percent changes are computed as %Δ=100 ∣*M_test_*−*M_baseline_*∣/∣*M_test_*∣.

**Table S7.** The value of the parameter k*_1_*, which is employed in the process of fitting the mathematical model to the experimental data for each cancer cell line.

| **Experimental study** | $k_{1}$ |
| --- | --- |
| JC adenocarcinoma cancer cells (Dromi, Frenkel et al. 2007) | 0.51 day^−1^ |
| CWR22 prostate adenocarcinoma cancer cells (Hagtvet, Evjen et al. 2011) | 0.38 day^−1^ |

**Table S8.** Tumor volume at treatment initiation and normalized growth-stage ratio for each proliferation rate *k_1_​* and timing shift relative to the reference protocol (day 0). Ratios are computed as *V_treat_* (Δt) / V*_treat_* (0) for each *k_1_*.

| ***k_1_* (day^-1^)** | ***V_treat_* ​(-4) mm³ (ratio)** | ***V_treat_* ​(-2) mm³ (ratio)** | ***V_treat_* ​(0) mm³ (ratio)** | ***V_treat_* ​(+2) mm³ (ratio)** | ***V_treat_* ​(+4) mm³ (ratio)** |
| --- | --- | --- | --- | --- | --- |
| 0.36 | 33.00 (0.62) | 41.84 (0.79) | 52.95 (1.00) | 66.92 (1.26) | 84.52 (1.60) |
| 0.44 | 61.98 (0.55) | 83.80 (0.74) | 112.88 (1.00) | 151.75 (1.34) | 204.13 (1.81) |
| 0.51 | 108.63 (0.49) | 155.24 (0.70) | 221.21 (1.00) | 314.38 (1.42) | 446.23 (2.02) |
| 0.65 | 337.78 (0.39) | 541.73 (0.63) | 857.07 (1.00) | 1351.4 (1.58) | 2115.1 (2.47) |

**Table S9.** Release rates of drug from TSLs at different temperatures (Needham and Dewhirst 2001, Gasselhuber, Dreher et al. 2012, Tagami, May et al. 2012, Rezaeian, Sedaghatkish et al. 2019, Souri, Soltani et al. 2021, Moradi Kashkooli, Souri et al. 2023, Tehrani, Moradi Kashkooli et al. 2024). The release rate constants *k*_rel_ (s^-1^) were interpolated and the corresponding fitted curves and fitting equations are presented in **Fig. S6**.

| **Temperature *T*_t_ (°C)** | 37 | 38 | 39 | 40 | 41 | 42 |
| --- | --- | --- | --- | --- | --- | --- |
| **Ultra-fast release rates *k*_rel_ (s^-1^)** | 0.0003 | 0.0047 | 0.142 | 0.221 | 0.3 | 0.3 |
| **Fast release rates *k*_rel_ (s^-1^)** | 0.00417 | 0.00545 | 0.0149 | 0.0282 | 0.0425 | 0.05409 |
| **Intermediate release rates *k*_rel_ (s^-1^)** | 0.002502 | 0.00327 | 0.00894 | 0.01692 | 0.0255 | 0.032454 |
| **Slow release rates *k*_rel_ (s^-1^)** | 0.000417 | 0.000545 | 0.00149 | 0.00282 | 0.00425 | 0.005409 |

**Table S10.** Nanocarrier sizes and associated parameters analyzed in this study.

| ***r*_s_ [nm]** | ***α* [-]** | ***D*_l_** **[m^2^/s]** |
| --- | --- | --- |
| 10 | 20  (Stylianopoulos, Economides et al. 2015) | 7.58 ×10^−12^  (Pluen, Boucher et al. 2001) |
| 50 | 10^4^  (Dawidczyk, Kim et al. 2014) | 6.31 ×10^−13^  (Pluen, Boucher et al. 2001) |
| 65 | 15000  (Dawidczyk, Kim et al. 2014) | 4.06 ×10^−13^  (Pluen, Boucher et al. 2001) |

**3. References**

Ambrosi, D. and F. Mollica (2002). "On the mechanics of a growing tumor." International Journal of Engineering Science **40**(12): 1297-1316.

Barsoum, I. B., C. A. Smallwood, D. R. Siemens and C. H. Graham (2014). "A mechanism of hypoxia-mediated escape from adaptive immunity in cancer cells." Cancer Res **74**(3): 665-674.

Billy, F., B. Ribba, O. Saut, H. Morre-Trouilhet, T. Colin, D. Bresch, J. P. Boissel, E. Grenier and J. P. Flandrois (2009). "A pharmacologically based multiscale mathematical model of angiogenesis and its use in investigating the efficacy of a new cancer treatment strategy." J Theor Biol **260**(4): 545-562.

Burroughs, N. J., B. M. P. M. Oliveira, A. A. Pinto and M. Ferreira (2011). "Immune response dynamics." Mathematical and Computer Modelling **53**(7-8): 1410-1419.

Byrne, H. and L. Preziosi (2003). "Modelling solid tumour growth using the theory of mixtures." Mathematical medicine and biology : a journal of the IMA **20**(4): 341-366.

Casciari, J. J., S. V. Sotirchos and R. M. Sutherland (1992). "Mathematical modelling of microenvironment and growth in EMT6/Ro multicellular tumour spheroids." Cell Prolif **25**(1): 1-22.

Casciari, J. J., S. V. Sotirchos and R. M. Sutherland (1992). "Variations in tumor cell growth rates and metabolism with oxygen concentration, glucose concentration, and extracellular pH." J Cell Physiol **151**(2): 386-394.

Chauhan, V. P., T. Stylianopoulos, Y. Boucher and R. K. Jain (2011). "Delivery of molecular and nanomedicine to tumors: Transport barriers and strategies." Annual Reviews Chemical and Biomolecular Engineering **2**: 281-298.

Chauhan, V. P., T. Stylianopoulos, J. D. Martin, Z. Popovic, O. Chen, W. S. Kamoun, M. G. Bawendi, D. Fukumura and R. K. Jain (2012). "Normalization of tumour blood vessels improves the delivery of nanomedicines in a size-dependent manner." Nature Nanotechnology **7**: 383-388.

Ciarletta, P. (2013). "Buckling instability in growing tumor spheroids." Phys Rev Lett **110**: 158102.

Conley, S. J., E. Gheordunescu, P. Kakarala, B. Newman, H. Korkaya, A. N. Heath, S. G. Clouthier and M. S. Wicha (2012). "Antiangiogenic agents increase breast cancer stem cells via the generation of tumor hypoxia." Proc Natl Acad Sci U S A **109**(8): 2784-2789.

Culshaw, R. V. and S. Ruan (2000). "A delay-differential equation model of HIV infection of CD4+ T-cells." Mathematical biosciences **165**(1): 27-39.

Dawidczyk, C. M., C. Kim, J. H. Park, L. M. Russell, K. H. Lee, M. G. Pomper and P. C. Searson (2014). "State-of-the-art in design rules for drug delivery platforms: lessons learned from FDA-approved nanomedicines." J Control Release **187**: 133-144.

De Palma, M. and R. K. Jain (2017). "CD4(+) T Cell Activation and Vascular Normalization: Two Sides of the Same Coin?" Immunity **46**(5): 773-775.

de Pillis, L. G. (2013). "Mathematical modeling of the regulatory T cell effects on renal cell carcinoma treatment."

de Pillis, L. G., A. E. Radunskaya and C. L. Wiseman (2005). "A validated mathematical model of cell-mediated immune response to tumor growth." Cancer Research **65**(17): 7950-7958.

Deen, W. M. (1987). "Hindered Transport of Large molecules in Liquid-Filled Pores." AIChE J **33**(9): 1409-1425.

Dromi, S., V. Frenkel, A. Luk, B. Traughber, M. Angstadt, M. Bur, J. Poff, J. Xie, S. K. Libutti, K. C. P. Li and B. J. Wood (2007). "Pulsed-High Intensity Focused Ultrasound and Low Temperature–Sensitive Liposomes for Enhanced Targeted Drug Delivery and Antitumor Effect." Clinical Cancer Research **13**(9): 2722-2727.

Eder, M., S. Raith, J. Jalali, A. Volf, M. Settles, H. G. Machens and L. Kovacs (2014). "Comparison of different material models to simulate 3-d breast deformations using finite element analysis." Ann Biomed Eng **42**(4): 843-857.

Eikenberry, S. (2009). "A tumor cord model for doxorubicin delivery and dose optimization in solid tumors." Theor Biol Med Model **6**: 16.

Fouchet, D. and R. Regoes (2008). "A population dynamics analysis of the interaction between adaptive regulatory T cells and antigen presenting cells." PLoS One **3**(5): e2306.

Gasselhuber, A., M. R. Dreher, F. Rattay, B. J. Wood and D. Haemmerich (2012). "Comparison of Conventional Chemotherapy, Stealth Liposomes and Temperature-Sensitive Liposomes in a Mathematical Model." PLOS ONE **7**(10): e47453.

Gevertz, J. L. and S. Torquato (2006). "Modeling the effects of vasculature evolution on early brain tumor growth." J Theor Biol **243**(4): 517-531.

Goh, Y.-M. F., H. L. Kong and C.-H. Wang (2001). "Simulation of the Delivery of Doxorubicin to Hepatoma." Pharmaceutical Research **18**(6): 761-770.

Goldman, A., B. Majumder, A. Dhawan, S. Ravi, D. Goldman, M. Kohandel, P. K. Majumder and S. Sengupta (2015). "Temporally sequenced anticancer drugs overcome adaptive resistance by targeting a vulnerable chemotherapy-induced phenotypic transition." Nat Commun **6**: 6139.

Hadjigeorgiou, A. G. and T. Stylianopoulos (2024). "Hybrid model of tumor growth, angiogenesis and immune response yields strategies to improve antiangiogenic therapy." npj Biological Physics and Mechanics **1**(1): 4.

Hagtvet, E., T. J. Evjen, D. R. Olsen, S. L. Fossheim and E. A. Nilssen (2011). "Ultrasound enhanced antitumor activity of liposomal doxorubicin in mice." Journal of Drug Targeting **19**(8): 701-708.

Håkansson, B., P. Carlsson and A. Tjellström (1986). "The mechanical point impedance of the human head, with and without skin penetration." The Journal of the Acoustical Society of America **80**(4): 1065-1075.

Hermann, P. C., S. L. Huber, T. Herrler, A. Aicher, J. W. Ellwart, M. Guba, C. J. Bruns and C. Heeschen (2007). "Distinct populations of cancer stem cells determine tumor growth and metastatic activity in human pancreatic cancer." Cell Stem Cell **1**(3): 313-323.

Holzapfel, G. A., T. C. Gasser and R. W. Ogden (2000). "A new constitutive framework for arterial wall mechanics and a comparative study of material models." J. Elasticity **61**: 1-48.

Huang, J., R. G. Holt, R. O. Cleveland and R. A. Roy (2004). "Experimental validation of a tractable numerical model for focused ultrasound heating in flow-through tissue phantoms." The Journal of the Acoustical Society of America **116**(4): 2451-2458.

Huang, Y., M. Snuderl and R. K. Jain (2011). "Polarization of tumor-associated macrophages: a novel strategy for vascular normalization and antitumor immunity." Cancer Cell **19**(1): 1-2.

Huang, Y., T. Stylianopoulos, D. G. Duda, D. Fukumura and R. K. Jain (2013). "Benefits of vascular normalization are dose and time dependent--letter." Cancer Res **73**(23): 7144-7146.

Kerr, D. J., A. M. Kerr, R. I. Freshney and S. B. Kaye (1986). "Comparative intracellular uptake of adriamycin and 4'-deoxydoxorubicin by non-small cell lung tumor cells in culture and its relationship to cell survival." Biochem Pharmacol **35**(16): 2817-2823.

Kim, Y., M. A. Stolarska and H. G. Othmer (2011). "The role of the microenvironment in tumor growth and invasion." Progress in biophysics and molecular biology **106**(2): 353-379.

Linde, N., W. Lederle, S. Depner, N. van Rooijen, C. M. Gutschalk and M. M. Mueller (2012). "Vascular endothelial growth factor-induced skin carcinogenesis depends on recruitment and alternative activation of macrophages." J Pathol **227**(1): 17-28.

Liu, G., X. Yuan, Z. Zeng, P. Tunici, H. Ng, I. R. Abdulkadir, L. Lu, D. Irvin, K. L. Black and J. S. Yu (2006). "Analysis of gene expression and chemoresistance of CD133+ cancer stem cells in glioblastoma." Mol Cancer **5**: 67.

MacLaurin, J., J. Chapman, G. W. Jones and T. Roose (2012). "The buckling of capillaries in solid tumours." Proc. R. Soc. A **468**: 4123-4145.

Mahlbacher, G., L. T. Curtis, J. Lowengrub and H. B. Frieboes (2018). "Mathematical modeling of tumor-associated macrophage interactions with the cancer microenvironment." J Immunother Cancer **6**(1): 10.

Mascheroni, P., M. Carfagna, A. Grillo, D. Boso and B. A. Schrefler (2018). "An avascular tumor growth model based on porous media mechanics and evolving natural states." Mathematics and Mechanics of Solids **23**(4): 686-712.

Milberg, O., C. Gong, M. Jafarnejad, I. H. Bartelink, B. Wang, P. Vicini, R. Narwal, L. Roskos and A. S. Popel (2019). "A QSP Model for Predicting Clinical Responses to Monotherapy, Combination and Sequential Therapy Following CTLA-4, PD-1, and PD-L1 Checkpoint Blockade." Sci Rep **9**(1): 11286.

Mok, W., T. Stylianopoulos, Y. Boucher and R. K. Jain (2009). "Mathematical modeling of herpes simplex virus distribution in solid tumors: implications for cancer gene therapy." Clin Cancer Res **15**(7): 2352-2360.

Moradi Kashkooli, F., A. Bhandari, B. Gu, M. C. Kolios, M. Kohandel and W. Zhan (2025). "Multiphysics modelling enhanced by imaging and artificial intelligence for personalised cancer nanomedicine: Foundations for clinical digital twins." Journal of Controlled Release **386**: 114138.

Moradi Kashkooli, F., T. K. Hornsby, M. C. Kolios and J. Tavakkoli (2024). "Ultrasound-mediated nano-sized drug delivery systems for cancer treatment: Multi-scale and multi-physics computational modeling." WIREs Nanomedicine and Nanobiotechnology **16**(1): e1913.

Moradi Kashkooli, F., A. Jakhmola, T. K. Hornsby, J. Tavakkoli and M. C. Kolios (2023). "Ultrasound-mediated nano drug delivery for treating cancer: Fundamental physics to future directions." Journal of Controlled Release **355**: 552-578.

Moradi Kashkooli, F., M. Soltani and M.-H. Hamedi (2020). "Drug delivery to solid tumors with heterogeneous microvascular networks: Novel insights from image-based numerical modeling." European Journal of Pharmaceutical Sciences **151**: 105399.

Moradi Kashkooli, F., M. Souri, J. Tavakkoli and M. C. Kolios (2023). "A spatiotemporal computational model of focused ultrasound heat-induced nano-sized drug delivery system in solid tumors." Drug Delivery **30**(1): 2219871.

Mow, V. C., S. C. Kuei, W. M. Lai and C. G. Armstrong (1980). "Biphasic creep and stress relaxation of articular cartilage in compression? Theory and experiments." Journal of Biomechanical Engineering **102**(1): 73-84.

Mpekris, F., S. Angeli, A. P. Pirentis and T. Stylianopoulos (2015). "Stress-mediated progression of solid tumors: effect of mechanical stress on tissue oxygenation, cancer cell proliferation, and drug delivery." Biomech Model Mechanobiol **14**(6): 1391-1402.

Mpekris, F., J. W. Baish, T. Stylianopoulos and R. K. Jain (2017). "Role of vascular normalization in benefit from metronomic chemotherapy." Proc Natl Acad Sci U S A **114**(8): 1994-1999.

Mpekris, F., M. Panagi, A. Charalambous, C. Voutouri, C. Michael, A. Papoui and T. Stylianopoulos (2024). "A synergistic approach for modulating the tumor microenvironment to enhance nano-immunotherapy in sarcomas." Neoplasia **51**: 100990.

Mpekris, F., C. Voutouri, J. W. Baish, D. G. Duda, L. L. Munn, T. Stylianopoulos and R. K. Jain (2020). "Combining microenvironment normalization strategies to improve cancer immunotherapy." Proc Natl Acad Sci U S A **117**(7): 3728-3737.

Mpekris, F., C. Voutouri, M. Panagi, J. W. Baish, R. K. Jain and T. Stylianopoulos (2022). "Normalizing tumor microenvironment with nanomedicine and metronomic therapy to improve immunotherapy." J Control Release **345**: 190-199.

Mpekris, F., C. Voutouri, P. Papageorgis and T. Stylianopoulos (2018). "Stress alleviation strategy in cancer treatment: Insights from a mathematical model." Z Angew Math Mech: 1-12.

Namakshenas, P. and A. Mojra (2023). "Efficient drug delivery to hypoxic tumors using thermosensitive liposomes with encapsulated anti-cancer drug under high intensity pulsed ultrasound." International Journal of Mechanical Sciences **237**: 107818.

Needham, D. and M. W. Dewhirst (2001). "The development and testing of a new temperature-sensitive drug delivery system for the treatment of solid tumors." Advanced Drug Delivery Reviews **53**(3): 285-305.

Netti, P. A., D. A. Berk, M. A. Swartz, A. J. Grodzinsky and R. K. Jain (2000). "Role of extracellular matrix assembly in interstitial transport in solid tumors." Cancer Res **60**(9): 2497-2503.

Panagi, M., F. Mpekris, C. Voutouri, A. G. Hadjigeorgiou, C. Symeonidou, E. Porfyriou, C. Michael, A. Stylianou, J. D. Martin, H. Cabral, A. Constantinidou and T. Stylianopoulos (2024). "Stabilizing Tumor-Resident Mast Cells Restores T-Cell Infiltration and Sensitizes Sarcomas to PD-L1 Inhibition." Clin Cancer Res **30**(11): 2582-2597.

Papageorgis, P., C. Polydorou, F. Mpekris, C. Voutouri, E. Agathokleous, C. P. Kapnissi-Christodoulou and T. Stylianopoulos (2017). "Tranilast-induced stress alleviation in solid tumors improves the efficacy of chemo- and nanotherapeutics in a size-independent manner." Sci Rep **7**: 46140.

Perelson, A. S., D. E. Kirschner and R. De Boer (1993). "Dynamics of HIV infection of CD4+ T cells." Math Biosci **114**(1): 81-125.

Pirentis, A. P., C. Polydorou, P. Papageorgis, C. Voutouri, F. Mpekris and T. Stylianopoulos (2015). "Remodeling of extracellular matrix due to solid stress accumulation during tumor growth." Connective tissue research **56**(5): 345-354.

Plank, M. J., B. D. Sleeman and P. F. Jones (2004). "The role of the angiopoietins in tumour angiogenesis." Growth Factors **22**(1): 1-11.

Pluen, A., Y. Boucher, S. Ramanujan, T. D. McKee, T. Gohongi, E. di Tomaso, E. B. Brown, Y. Izumi, R. B. Campbell, D. A. Berk and R. K. Jain (2001). "Role of tumor-host interactions in interstitial diffusion of macromolecules: cranial vs. subcutaneous tumors." Proc Natl Acad Sci U S A **98**(8): 4628-4633.

Popel, A. S. (1989). "Theory of oxygen transport to tissue." Crit Rev Biomed Eng **17**(3): 257-321.

Rezaeian, M., A. Sedaghatkish and M. Soltani (2019). "Numerical modeling of high-intensity focused ultrasound-mediated intraperitoneal delivery of thermosensitive liposomal doxorubicin for cancer chemotherapy." Drug Delivery **26**(1): 898-917.

Rodriguez, E. K., A. Hoger and A. D. McCulloch (1994). "Stress-dependent finite growth in soft elastic tissues." J Biomech **27**(4): 455-467.

Rolny, C., M. Mazzone, S. Tugues, D. Laoui, I. Johansson, C. Coulon, M. L. Squadrito, I. Segura, X. Li, E. Knevels, S. Costa, S. Vinckier, T. Dresselaer, P. Akerud, M. De Mol, H. Salomaki, M. Phillipson, S. Wyns, E. Larsson, I. Buysschaert, J. Botling, U. Himmelreich, J. A. Van Ginderachter, M. De Palma, M. Dewerchin, L. Claesson-Welsh and P. Carmeliet (2011). "HRG inhibits tumor growth and metastasis by inducing macrophage polarization and vessel normalization through downregulation of PlGF." Cancer Cell **19**(1): 31-44.

Roose, T., P. A. Netti, L. L. Munn, Y. Boucher and R. K. Jain (2003). "Solid stress generated by spheroid growth estimated using a linear poroelasticity model." Microvasc Res **66**(3): 204-212.

Samani, A., J. Zubovits and D. Plewes (2007). "Elastic moduli of normal and pathological human breast tissues: an inversion-technique-based investigation of 169 samples." Phys Med Biol **52**(6): 1565-1576.

Schmidt, M. M. and K. D. Wittrup (2009). "A modeling analysis of the effects of molecular size and binding affinity on tumor targeting." Molecular cancer therapeutics **8**(10): 2861-2871.

Schugart, R. C., A. Friedman, R. Zhao and C. K. Sen (2008). "Wound angiogenesis as a function of tissue oxygen tension: a mathematical model." Proc Natl Acad Sci U S A **105**(7): 2628-2633.

Sheu, T., M. Solovchuk, A. Chen and T. Marc (2011). "On an acoustics-thermal-fluid coupling model for the prediction of temperature elevation in liver tumor." International Journal of Heat and Mass Transfer **54**.

Skalak, R., S. Zargaryan, R. K. Jain, P. A. Netti and A. Hoger (1996). "Compatibility and the genesis of residual stress by volumetric growth." J Math Biol **34**(8): 889-914.

Souri, M., M. Kiani Shahvandi, M. Chiani, F. Moradi Kashkooli, A. Farhangi, M. R. Mehrabi, A. Rahmim, V. M. Savage and M. Soltani (2023). "Stimuli-sensitive nano-drug delivery with programmable size changes to enhance accumulation of therapeutic agents in tumors." Drug Delivery **30**(1): 2186312.

Souri, M., M. Soltani and F. Moradi Kashkooli (2021). "Computational modeling of thermal combination therapies by magneto-ultrasonic heating to enhance drug delivery to solid tumors." Scientific Reports **11**(1): 19539.

Stockmann, C., A. Doedens, A. Weidemann, N. Zhang, N. Takeda, J. I. Greenberg, D. A. Cheresh and R. S. Johnson (2008). "Deletion of vascular endothelial growth factor in myeloid cells accelerates tumorigenesis." Nature **456**(7223): 814-818.

Stylianopoulos, T., E. A. Economides, J. W. Baish, D. Fukumura and R. K. Jain (2015). "Towards Optimal Design of Cancer Nanomedicines: Multi-stage Nanoparticles for the Treatment of Solid Tumors." Ann Biomed Eng **43**(9): 2291-2300.

Stylianopoulos, T. and R. K. Jain (2013). "Combining two strategies to improve perfusion and drug delivery in solid tumors." Proc Natl Acad Sci U S A **110**((46)): 18632-18637.

Stylianopoulos, T., J. D. Martin, M. Snuderl, F. Mpekris, S. R. Jain and R. K. Jain (2013). "Coevolution of solid stress and interstitial fluid pressure in tumors during progression: Implications for vascular collapse." Cancer research **73**(13): 3833-3841.

Stylianopoulos, T., A. Yeckel, J. J. Derby, X. J. Luo, M. S. Shephard, E. A. Sander and V. H. Barocas (2008). "Permeability calculations in three-dimensional isotropic and oriented fiber networks." Phys Fluids (1994) **20**(12): 123601.

Taber, L. A. (2008). "Theoretical study of Beloussov's hyper-restoration hypothesis for mechanical regulation of morphogenesis." Biomech Model Mechanobiol **7**(6): 427-441.

Tagami, T., J. P. May, M. J. Ernsting and S.-D. Li (2012). "A thermosensitive liposome prepared with a Cu2+ gradient demonstrates improved pharmacokinetics, drug delivery and antitumor efficacy." Journal of Controlled Release **161**(1): 142-149.

Tehrani, M. H. H., F. Moradi Kashkooli and M. Soltani (2024). "Effect of tumor heterogeneity on enhancing drug delivery to vascularized tumors using thermo-sensitive liposomes triggered by hyperthermia: A multi-scale and multi-physics computational model." Computers in Biology and Medicine **170**: 108050.

Tian, L., A. Goldstein, H. Wang, H. Ching Lo, I. Sun Kim, T. Welte, K. Sheng, L. E. Dobrolecki, X. Zhang, N. Putluri, T. L. Phung, S. A. Mani, F. Stossi, A. Sreekumar, M. A. Mancini, W. K. Decker, C. Zong, M. T. Lewis and X. H. Zhang (2017). "Mutual regulation of tumour vessel normalization and immunostimulatory reprogramming." Nature **544**(7649): 250-254.

Todaro, M., M. D'Asaro, N. Caccamo, F. Iovino, M. G. Francipane, S. Meraviglia, V. Orlando, C. La Mendola, G. Gulotta, A. Salerno, F. Dieli and G. Stassi (2009). "Efficient killing of human colon cancer stem cells by gammadelta T lymphocytes." J Immunol **182**(11): 7287-7296.

Tracqui, P. (1995). "From passive diffusion to active cellular migration in mathematical models of tumour invasion." Acta Biotheor **43**(4): 443-464.

Voutouri, C., N. D. Kirkpatrick, E. Chung, F. Mpekris, J. W. Baish, L. L. Munn, D. Fukumura, T. Stylianopoulos and R. K. Jain (2019). "Experimental and computational analyses reveal dynamics of tumor vessel cooption and optimal treatment strategies." Proc Natl Acad Sci U S A **116**(7): 2662-2671.

Voutouri, C., F. Mpekris, P. Papageorgis, A. D. Odysseos and T. Stylianopoulos (2014). "Role of constitutive behavior and tumor-host mechanical interactions in the state of stress and growth of solid tumors." PLoS One **9**(8): e104717.

Voutouri, C. and T. Stylianopoulos (2014). "Evolution of osmotic pressure in solid tumors." J Biomech **47**(14): 3441-3447.

Wang, Q., C. Liu, F. Zhu, F. Liu, P. Zhang, C. Guo, X. Wang, H. Li, C. Ma, W. Sun, Y. Zhang, W. Chen and L. Zhang (2010). "Reoxygenation of hypoxia-differentiated dentritic cells induces Th1 and Th17 cell differentiation." Mol Immunol **47**(4): 922-931.

Wu, N. Z., B. Klitzman, G. Rosner, D. Needham and M. W. Dewhirst (1993). "Measurement of Material Extravasation in Microvascular Networks Using Fluorescence Video-Microscopy." Microvascular Research **46**(2): 231-253.

Xu, G., P. V. Bayly and L. A. Taber (2009). "Residual stress in the adult mouse brain." Biomech Model Mechanobiol **8**(4): 253-262.

Xu, G., P. S. Kemp, J. A. Hwu, A. M. Beagley, P. V. Bayly and L. A. Taber (2010). "Opening angles and material properties of the early embryonic chick brain." J Biomech Eng **132**(1): 011005.

Zhan, W., W. Gedroyc and X. Y. Xu (2019). "Towards a multiphysics modelling framework for thermosensitive liposomal drug delivery to solid tumour combined with focused ultrasound hyperthermia." Biophysics Reports **5**(1): 43-59.
